# Association between parental psychiatric disorders and risk of offspring autism spectrum disorder: a Swedish and Finnish population-based cohort study

**DOI:** 10.1016/j.lanepe.2024.100902

**Published:** 2024-04-23

**Authors:** Weiyao Yin, Anna Pulakka, Abraham Reichenberg, Alexander Kolevzon, Jonas F. Ludvigsson, Kari Risnes, Marius Lahti-Pulkkinen, Martina Persson, Michael E. Silverman, Ulrika Åden, Eero Kajantie, Sven Sandin

**Affiliations:** a Department of Medical Epidemiology and Biostatistics, Karolinska Institutet, Stockholm, Sweden; b Department of Psychiatry, Icahn School of Medicine at Mount Sinai, New York, USA; c Seaver Center for Autism Research and Treatment, Icahn School of Medicine at Mount Sinai, New York, NY, USA; d Department of Environmental Medicine and Public Health, Icahn School of Medicine at Mount Sinai, New York, NY, USA; e Population Health Unit, Finnish Institute for Health and Welfare, Helsinki and Oulu, Finland; f Research Unit of Population Health, Faculty of Medicine, University of Oulu, Oulu, Finland; g Department of Pediatrics, Örebro University Hospital, Örebro, Sweden; h Department of Clinical and Molecular Medicine, NTNU, Trondheim, Norway; i Children’s Clinic, St Olav University Hospital, Trondheim, Norway; j Department of Psychology and Logopedics, Faculty of Medicine, University of Helsinki, Finland; k Department of Medicine, Clinical Epidemiological Unit, Karolinska Institutet, Stockholm, Sweden; l Department of Clinical Science and Education, Division of Pediatrics, Karolinska Institutet, Stockholm, Sweden; m Sachsska Childrens’ and Youth Hospital, Stockholm, Sweden; n Department of Women’s and Children’s Health, Karolinska Institutet, Stockholm, Sweden; o Department of Biomedical and Clinical Sciences, Linköping University, Sweden; p Clinical Medicine Research Unit, MRC Oulu, Oulu University Hospital and University of Oulu, Oulu, Finland

**Keywords:** Autism, Mental illness, Family risk, Cohort, Epidemiology

## Abstract

**Background:**

Roughly more than one in six adults worldwide suffer from psychiatric conditions. Sporadic studies have associated parental psychiatric disorders with autism spectrum disorder in offspring. Comprehensively examining the association between parental psychiatric disorders and offspring autism spectrum disorder is needed to guide health policies, and to inform etiologic studies.

**Methods:**

We included all children born in Sweden and Finland 1997–2016. Diagnoses were clinically ascertained from National Registers through 2017. We calculated adjusted hazard ratios (aHRs) and 95% confidence intervals (CIs) for autism spectrum disorder in offspring of fathers and mothers with psychiatric disorders, in both parents jointly and across co-occurring conditions.

**Findings:**

Among 2,505,842 children, 33,612 were diagnosed with autism spectrum disorder, of which 20% had a parent with psychiatric disorders. The risk of autism spectrum disorder was increased across all psychiatric disorders in fathers (Sweden: aHR = 2.02, 95% CI = 1.92–2.12; Finland: aHR = 1.63, 95% CI = 1.50–1.77), mothers (Sweden: aHR = 2.34, 95% CI = 2.24–2.43; Finland aHR = 2.12, 95% CI = 1.92–2.28), or both parents (Sweden: aHR = 3.76, 95% CI = 3.48–4.07; Finland aHR = 3.61, 95% CI = 3.20–4.07), compared to neither parents. Co-occurrence of parental psychiatric disorders further increased risk (e.g., Sweden: for one, two or ≥three different diagnostic categories compared to no diagnosis, in fathers aHR = 1.81, 2.07, 2.52; in mothers aHR = 2.05, 2.63, 3.57).

**Interpretation:**

Psychiatric disorders in both parents conveyed the highest risk of offspring autism spectrum disorder, followed by mothers and then fathers. The risk increased with number of co-occurring disorders. All parental psychiatric disorders were associated with increased the risk of autism spectrum disorder. To reliably assess the risk of autism spectrum disorder in children, a comprehensive history incorporating the full range of parental psychiatric disorders is needed beyond solely focusing on familial autism spectrum disorder.

**Funding:**

Swedish-Research-Council-2021-0214.

**Research in context**

**Evidence before this study**

Sporadic studies have associated parental psychiatric disorders with increased risk of autism spectrum disorder. We searched PubMed from the database inception to date December 31, 2022, using the search terms (“Psychiatric disorders” OR “Mental illness” OR “Psychiatric condition” OR “Psychiatric disease” OR “Psychiatric history”) AND (“Autism” OR “Autism spectrum disorders” OR “Autistic disorder”) AND (“Population-based” OR “Cohort” OR “Register”) AND “Risk”. No language restrictions were applied. Results derived from previous studies suffered from methodological limitations, insufficient statistical precision and heterogeneous samples. Crucial information is lacking on combined effect of both parents, influences of comorbid psychiatric disorders in parents, and risk estimates for a detailed breakdown of psychiatric disorders.

**Added value of this study**

Our population-based study of over 2.5 million births in Sweden and Finland, comprehensively assessed contribution from parental psychiatric disorders to the incidence of autism spectrum disorder in offspring. A general risk pattern was observed, wherein a psychiatric disorder in both parents conveyed the highest risk of autism spectrum disorder in offspring, followed by mothers only and then fathers only. The risk of autism spectrum disorder increased in cases of all parental psychiatric disorders, and increased further with each additional co-occurring psychiatric disorder. The individual-level risk estimates can provide insights into the complex risk structure underpinning the association between parental psychiatric disorders and offspring autism spectrum disorder, and can potentially be used by physicians and public health workers, combined with other risk factors, to provide risk estimates for autism spectrum disorder and in consulting to families.

**Implications of all the available evidence**

Roughly more than one in six adults in the world suffer from psychiatric conditions. In our study, 20% of children with autism spectrum disorder had at least one parent with psychiatric disorders. Our findings suggest that a comprehensive evaluation of family history that incorporates the full range of parental psychiatric disorders is necessary in assessing the risk of autism spectrum disorder, rather than solely focusing on familial autism spectrum disorder. These findings will help to identify children at high risk for autism spectrum disorder.

## Introduction

Autism spectrum disorder is a chronic neurodevelopmental disorder highly heritable,^1^ but also arises from non-heritable factors.^2^, ^3^, ^4^, ^5^ As for genetic risk, the risk-pattern is complex,^6^ and shares genetic architecture with other common psychiatric disorders.^7^ Roughly more than one in six adults in the world suffer from psychiatric conditions.^8^ Previous studies have revealed increased risk of autism spectrum disorder in offspring of parents with psychiatric disorders. Still, there is a lack of large population studies thoroughly examining maternal versus paternal psychiatric disorders as risk factors, and the combined influences of both parents. Furthermore, it is known that mental illness has a polygenic basis — many alleles at various genetic loci, mostly with small effect sizes, adding up to an overall risk.^9^^,^^10^ Several co-occurring psychiatric disorders in a parent may therefore indicate a higher genetic loading for risk across a wider range of psychiatric spectrum. Yet, this genetic architecture of psychiatric disorders has rarely been reflected in studies of autism spectrum disorder.

Regardless of the precise mechanism linking parental psychiatric disorders and offspring autism spectrum disorder, a well-informed public health debate on these matters requires a solid empirical foundation. Current information on the association between parental psychiatric conditions and offspring autism spectrum disorder is fragmented, based on inconsistent results derived from heterogeneous samples and with methodological limitations (Supplementary eTable S1).^11^, ^12^, ^13^, ^14^, ^15^, ^16^ Absence of statistical significance in under-powered studies can be often mistakenly interpreted as a null effect.^11^^,^^14^^,^^15^ Analyses based on postnatal parental diagnoses may be confounded by reverse causation.^12^, ^13^, ^14^ Lack of detailed data on diagnostic classification and specific disorders can lead to spurious results,^11^, ^12^, ^13^ and the few reports on the risk of autism spectrum disorder from specific parental psychiatric disorders have not taken into account the full range of different psychiatric disorders.^14^, ^15^, ^16^ Using Swedish and Finnish nationwide registers, we had the opportunity to comprehensively examine the association between parental psychiatric disorders and offspring autism spectrum disorder. In particular, we estimated whether the risk varies (1) by mother, father, or both parents combined, (2) as a function of comorbid psychiatric disorders, and (3) according to a detailed breakdown of psychiatric disorders.

## Methods

### Study design, setting and population

Our primary study sample included all children born in Sweden between 1997 and 2016, to parents born in Sweden, Denmark, Finland Iceland or Norway. We restricted the population to Nordic parents to minimize genetic and cultural confounding, and misclassification bias due to limited data availability. Cohort entry was set to 1997, the year International Classification of Diseases (ICD) version 10 was introduced in Sweden (1996 in Finland); see next section for more information. Biological parents were identified from the Swedish Medical Birth Register (MBR)^17^ and the Swedish Multi-generation Register.^18^ Data for vital status and emigration was derived from the Total Population Register. Individual-level data from different registers was linked via the Swedish personal identity number for each resident.^19^ The second sample, for the replicated study, of all children born to Nordic parents in Finland between 1997 and 2016 was collected from the Finnish Medical Birth Register.^20^^,^^21^ Each dataset was analysed separately, sharing computer code.

### Psychiatric disorders

Diagnoses of psychiatric disorders from specialist care were obtained from the Swedish National Patient Register (NPR) using the ICD. The register includes inpatient diagnoses since 1973 and outpatient diagnoses since approximately 2001. The ICD versions used were version 8 before 1987, version 9 from 1987 to 1996, and version 10 from 1997 onwards (see Supplementary eTable S2). It should be noted that the two versions of the ICD system used for diagnosis differ between the child and parental generations. The data quality of the Swedish NPR has been verified,^19^ and validated for diagnoses of multiple psychiatric conditions.^22^ In the Finnish data, psychiatric diagnoses were extracted from the Finnish Care Register for Health Care (CRHC).^23^ We defined a psychiatric disorder in parents as any psychiatric diagnosis registered in the NPR or CRHC prior to birth of the child, and further classified as ‘mother only’ if present in mother but not the father, ‘father only’ if present in father but not in the mother, or ‘both’ if a disorder was present in both of the parents before the birth of the child.

### Outcomes

In Sweden as well as in Finland, all infants and preschool children undergo routine medical examinations and developmental assessments (motor skills, language, cognitive and social development).^24^ Children who are suspected of having autism spectrum disorder are referred to a specialist within child psychiatry/neurology unit or habilitation service. Diagnostic procedures and validity of diagnoses in Sweden and Finland have been published previously (Supplementary eTable S2).^23^^,^^25^

### Other covariates

Data on birth year, offspring sex, maternal age, maternal tobacco smoking during pregnancy, maternal body mass index (BMI) at the first prenatal visit and gestational age (in weeks) were extracted from the MBR. Paternal age was obtained from the multi-generation register. Paternal and maternal yearly disposable income (Swedish Krona) and the highest level of educational attainment (completed school years) was obtained from the Swedish government’s database for health insurance and labour market studies (LISA database). The covariate information in Finnish data were obtained from birth or statistical authorities (Supplementary eTable S21). All variables were defined before childbirth (Table 1).

**Table 1:** Cohort characteristics by any psychiatric disorder in parents.

Characteristics	Neither parent with psychiatric disorders Number of children (%)	Paternal psychiatric disorders only Number of children (%)	Maternal psychiatric disorders only Number of children (%)	Both parents with psychiatric disorders Number of children (%)
Number of individuals	1,268,419 (85.19)	73,553 (4.94)	122,634 (8.24)	24,314 (1.63)
Offspring sex (male, %)	653,087 (51.49)	37,748 (51.32)	63,115 (51.47)	12,532 (51.54)
Follow-up years^a^	11.3 (6.4–15.9)	7.3 (3.9–12.2)	6.8 (3.7–10.9)	5.3 (3.0–8.9)
Birth year				
1997–2001	324,088 (25.55)	9224 (12.54)	10,869 (8.86)	1293 (5.32)
2002–2006	342,634 (27.01)	13,448 (18.28)	20,201 (16.47)	2641 (10.86)
2007–2011	322,112 (25.39)	21,385 (29.07)	38,140 (31.10)	7063 (29.05)
2012–2016	279,585 (22.04)	29,496 (40.10)	53,424 (43.56)	13,317 (54.77)
Offspring ASD	20,947 (1.65)	1622 (2.21)	2747 (2.24)	648 (2.67)
Autistic Disorder	9120 (43.54)	837 (51.60)	1442 (52.49)	385 (59.41)
Asperger’s syndrome	6301 (30.08)	387 (23.86)	632 (23.01)	95 (14.66)
Other ASD	5526 (26.38)	398 (24.54)	673 (24.50)	168 (25.93)
Comorbid with ID	2762 (13.19)	228 (14.06)	359 (13.07)	105 (16.20)
Age at ASD (years)^a^	11.1 (7.5–14.3)	10.0 (6.4–13.3)	8.7 (5.7–12.1)	8.2 (5.1–11.7)
Preterm birth (<37 week)	73,090 (5.76)	4597 (6.25)	8919 (7.27)	2028 (8.34)
Gestational age				
<32 week	62,580 (4.93)	3872 (5.26)	7553 (6.16)	1736 (7.14)
32–36 week	230,837 (18.20)	13,613 (18.51)	27,662 (22.56)	5627 (23.14)
37–38	10,510 (0.83)	725 (0.99)	1366 (1.11)	292 (1.20)
≥39 week	964,492 (76.04)	55,343 (75.24)	86,053 (70.17)	16,659 (68.52)
Maternal age at delivery^a^	31 (28–34)	30 (26–34)	30 (26–34)	29 (25–33)
Paternal age at delivery^a^	33 (30–37)	33 (28–37)	32 (28–37)	32 (27–37)
Maternal yearly disposable income (SEK)^a^	143,262 (109,036–191,474)	151,339 (112,689–199,887)	146,353 (107,914–195,206)	140,278 (102,196–185,914)
Paternal yearly disposable income (SEK)^a^	227,219 (166,306–300,431)	209,427 (138,371–281,908)	244,467 (176,754–313,641)	178,352 (111,485–254,303)
Maternal education				
<9 years primary school	1000 (0.08)	271 (0.37)	675 (0.55)	432 (1.78)
9 years primary school	78,199 (6.17)	10,258 (13.95)	19,768 (16.12)	7514 (30.90)
1–2 years secondary school	183,878 (14.50)	10,155 (13.81)	16,794 (13.69)	4170 (17.15)
3 years secondary school	371,257 (29.27)	25,839 (35.13)	37,299 (30.41)	7046 (28.98)
1–2 years postgraduate	181,663 (14.32)	8183 (11.13)	14,471 (11.80)	1960 (8.06)
≥3 years postgraduate	442,623 (34.90)	18,529 (25.19)	33,093 (26.99)	3158 (12.99)
PhD	9799 (0.77)	318 (0.43)	534 (0.44)	34 (0.14)
Paternal education				
<9 years primary school	2677 (0.21)	942 (1.28)	488 (0.40)	637 (2.62)
9 years primary school	106,509 (8.40)	16,157 (21.97)	14,601 (11.91)	7641 (31.43)
1–2 years secondary school	291,203 (22.96)	17,091 (23.24)	22,693 (18.50)	5675 (23.34)
3 years secondary school	362,995 (28.62)	21,360 (29.04)	45,368 (36.99)	6794 (27.94)
1–2 years postgraduate	191,815 (15.12)	7086 (9.63)	15,020 (12.25)	1680 (6.91)
≥3 years postgraduate	297,403 (23.45)	10,487 (14.26)	23,415 (19.09)	1814 (7.46)
PhD	15,817 (1.25)	430 (0.58)	1049 (0.86)	73 (0.30)

ASD: autism spectrum disorders; ID: intellectual disability; Q1: 1st quartile (25th percentile), Q3: 3rd quartile (75th percentile); SEK: Swedish Krona.

a Median (Q1–Q3).

### Statistical analyses

The primary analysis was performed using Swedish population and a replication using the corresponding Finnish population. The association between parental psychiatric disorders before childbirth and risk of autism spectrum disorder in offspring was quantified by hazard ratios (HR) and associated two-sided 95% confidence intervals (CI), from Cox proportional hazards models,^26^ together with incidence rate of autism spectrum disorder (cases per 100,000 person years). To adjust for potential correlations between siblings of the same mother we applied robust standard errors. Each offspring was followed from birth until the first diagnosis of autism spectrum disorder, emigration, death, or the 31st December 2017 in Sweden and 31st December 2016 in Finland, whichever came first.

First, we examined the risk of autism spectrum disorder in the offspring associated with any psychiatric diagnosis before childbirth in fathers only, mothers only, and in both parents, compared to offspring of neither parent with any psychiatric diagnosis before childbirth, adjusting for offspring birth year as natural cubic splines (Model 1).^27^ Next, using a top-down perspective, we examined the risk of autism spectrum disorder by splitting parental diagnoses into six major categories (neurodevelopmental disorders, emotional and behavioral disorders of childhood origin and intellectual disability (NDD); schizophrenia and non-mood psychotic disorders; mood disorders; neurotic/behavioral disorders; psychoactive substance use; other/unspecific psychiatric disorders), and thereafter into sixteen specific disorders (Supplementary eMethod S1).^28^ Individuals with multiple psychiatric diagnoses can contribute to different diagnostic categories. Then, the risk of offspring autism spectrum disorder in relation to the number of co-occurring diagnoses in different major categories was examined, by diagnoses in one, two or ≥ three categories, compared to none psychiatric diagnosis, in mothers and fathers separately. Inverse Kaplan–Meier (KM) curves were used to depict the cumulative incidence of autism spectrum disorder. After quantifying the risk associated with maternal and paternal disorders, we wanted to better understand their joint contribution by including an interaction term. The pattern of this interaction was determined to be such that the combined effect was best described either as a sum of maternal and paternal effect, or as a product.^29^ The interaction pattern may guide future research into underlying causes and mechanisms. Examples of risk patterns of parental psychiatric disorders for offspring autism spectrum disorder were illustrated in Supplementary eFig. S1.

All statistical tests were performed on the two-sided 5% level of significance. We did not adjust the *p*-values for multiplicity of statistical tests. However, only a few statistical tests are needed to address study primary aims (psychiatric history in fathers only, mothers only and in both parents, versus parents without psychiatric history). The proportional hazards assumption of the Cox regression was visually examined by Schoenfeld residuals.^30^

#### Supplementary analyses

(1) Parental age, education, income and pregnancy-related risk factors (gestational age, maternal smoking and BMI) may influence the associations, not only as confounders but also as modifiers and/or mediators.^31^^,^^32^ Given that adjustment in regression analyses may introduce unknown biases, we did not include these factors in the *main* model (Model 1). Still, to explore whether these covariates impact the association, without resolving their exact potential roles, we adjusted for them in supplementary models. In Model 2, we adjusted for maternal and paternal age, education, and income levels. In Model 3, we further adjusted for pregnancy-related risk factors. (2) To examine the interaction between parental diagnosis, we quantified the joint effect by examining excess risk in addition to the sum of individual effects (HR_both_-HR_father_only_-HR_mother_only_+1),^29^ and by comparing HRs of the combined effect to the product of the parental hazards (HR_both_/(HR_father_only_∗HR_mother_only_)).^29^ (3) To test the robustness of the results, we restricted the analysis to (i) parents with disorders in only one major diagnostic category throughout, (ii) psychiatric diagnosis registered prior to one year before conception, (iii) psychiatric diagnoses registered at least twice in the Patient Register with an interval of more than 30 days, (iv) more severe autism spectrum disorder, i.e., Autistic Disorder (ICD-10 F84.0), (v) the family’s first born, (vi) offspring born 2007–2016, i.e., in the last decade, (vii) offspring born 1997–2012, i.e., at least 5 years old at the end of the follow-up), (viii) singletons, (ix) offspring without malformations; (x) to test if the main driver of the risk of autism spectrum disorder in offspring is parental autism spectrum disorder or it is similar to other diagnosis, we excluded offspring of parents with autism spectrum disorder, and examined offspring risk of autism spectrum disorder to any non-autism spectrum disorder psychiatric disorders in parents. (4) For pathways of risk, our earlier study shows that parents with psychiatric disorders are at higher risk of preterm/early-term delivery with increased risk of autism spectrum disorder.^2^^,^^33^ The role of preterm/early-term birth in the association between parental psychiatric history and offspring autism spectrum disorder, as a modifier or mediator, has not been appropriately examined using up-to-date statistical methods.^13^ We estimated the effect modification by including interaction terms and the mediated risk using Natural Effects Models.^34^ (5) We estimated the risk of autism spectrum disorder separately by offspring sex. (6) We replicated the analyses of any psychiatric diagnosis and the six major categories in the Finnish sample, and presented the results side-by-side as well as combined.^35^ Statistical analyses were performed using SAS software version 9.4.

### Role of the funding source

The funder had no role in the study design, data collection, data analysis, data interpretation, or drafting of the report.

## Results

Of the 1,499,701 children born in Sweden, we excluded 32 with implausible death/emigration date and 10,749 (0.72%) with missing data on covariates. The analytic cohort comprised 1,488,920 children, contributing 15,895,843 person-years during an average follow-up of 11 years (1st percentile = 1.2 years, 99th percentile = 20.8 years) (Supplementary eFig. S2). In total, 73,553 (4.94%) of fathers only, 122,634 (8.24%) of mothers only and 24,314 (1.63%) of both parents had a psychiatric diagnosis before childbirth. Among parents *without* a diagnosed psychiatric condition, 20,947 children had autism spectrum disorder (1.65%; 148 cases per 100,000 person-years). In comparison, 1622 children had autism spectrum disorder (2.21%; 262 cases per 100,000 person-years) if fathers only were affected with psychiatric disorders, 2747 children had autism spectrum disorder (2.24%; 289 cases per 100,000 person-years) if mothers only were affected, and 648 children had autism spectrum disorder (2.67%; 411 cases per 100,000 person-years) if both parents were affected.

Compared to parents that have never been diagnosed with a psychiatric disorder before childbirth, parents with psychiatric disorders before childbirth were younger, had lower level of education and income, and their offspring were more likely born preterm or early term, and to be diagnosed with autism spectrum disorder at an earlier age (Table 1).

### Parental psychiatric history and offspring autism spectrum disorder

#### Any psychiatric disorder

**Fig. 1:**
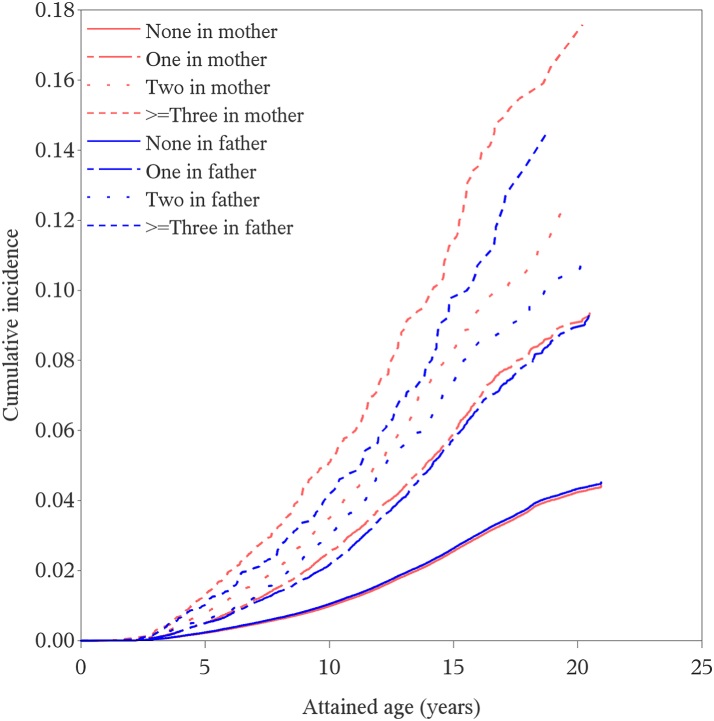
**Inverse Kaplan–Meier curves for offspring autism spectrum disorder by co-occurring psychiatric disorders in parents.** Note: Inverse Kaplan–Meier curves for cumulative incidence of autism spectrum disorder in the offspring, comparing mothers and fathers with any psychiatric disorder and without psychiatric disorder, by the co-occurring number of psychiatric disorders in different six major categories (Neurodevelopmental disorders, emotional and behavioral disorders of childhood origin and intellectual disability; Schizophrenia and other non-mood psychotic disorders; Mood disorders; Neurotic/behavioral disorders; Psychoactive substance use; Other/unspecific psychiatric disorders). Kaplan–Meier curves based on individuals number at risk (number diagnosed) at 0, 5, 10, 15, 20, and 21 years-of-age for offspring of *mothers* (red) with no psychiatric diagnosis 1,341,972 (0), 1,088,418 (2573), 745,519 (9466), 392,475 (18,274), 69,513 (22,507), and 0 (22,569); *mothers* with diagnoses in one major category 100,364 (0), 66,314 (387), 31,813 (1329), 11,672 (2023), 1811 (2267), and 0 (2271); *mothers* with diagnoses in two major categories 33,800 (0), 19,122 (189), 7216 (506), 2301 (717), 345 (777), and 0 (777); *mothers* with diagnoses in ≥ three major categories 12,784 (0), 6222 (104), 1993 (244), 633 (321), 89 (346), and 0 (347); offspring of *fathers* (blue) with no psychiatric diagnosis 1,391,053 (0), 1,117,877 (2851), 756,162 (10,324), 394,680 (19,334), 69,740 (23,633), and 0 (23,694); *fathers* with diagnoses in one major category 70,967 (0), 46,987 (269), 24,144 (837), 9987 (1429), 1603 (1634), and 0 (1638); *fathers* with diagnoses in two major categories 18,552 (0), 11,021 (78), 4913 (255), 1966 (400), 343 (441), and 0 (443); *fathers* with diagnoses in ≥ three major categories 8348 (0), 4106 (54), 1324 (129), 468 (174), 63 (189), and 0 (189).

There was an increased risk of autism spectrum disorder in offspring of parents with psychiatric disorders including in fathers only (HR = 2.02, 95% CI = 1.92–2.12), in mothers only (HR = 2.34, 95% CI = 2.24–2.43), and in both parents (HR = 3.76, 95% CI = 3.48–4.07), compared to un-diagnosed parents (Table 2). The risk was higher for offspring with maternal disorders only than for offspring with paternal only, and higher still when both parents were diagnosed than in case of maternal disorders only (Supplementary eTable S3). The presence of comorbid psychiatric disorders in a parent further increased the risk of autism spectrum disorder. For fathers with psychiatric disorders in only one major diagnostic category, the HR was 1.81 (95% CI = 1.72–1.90), with disorders in two diagnostic categories the HR was 2.07 (95% CI = 1.88–2.27), and disorders in ≥ three categories the HR was 2.52 (95% CI = 2.18–2.92). Results were aligned in mothers with psychiatric disorders in one (HR = 2.05, 95% CI = 1.96–2.14), two (HR = 2.63, 95% CI = 2.45–2.83) and ≥ three diagnostic categories (HR = 3.57, 95% CI = 3.21–3.98) (Fig. 1; Supplementary eFig. S3 for adjusted KM curves, and Supplementary eTable S5 for HRs of autism spectrum disorder).

**Table 2:** Parental psychiatric disorders before childbirth and risk of autism spectrum disorder in offspring.

Analysis group	Autism spectrum disorder (rate)	Total person-years	Model 1 HR (95% CI)	Model 2 HR (95% CI)
Any psychiatric disorder				
Neither parent	20,947 (147.8)	14,168,262	Reference	Reference
Father only	1622 (262.3)	618,392	2.02 (1.92–2.12)	1.59 (1.51–1.67)
Mother only	2747 (288.7)	951,641	2.34 (2.24–2.43)	1.95 (1.87–2.04)
Both parents	648 (411.3)	157,548	3.76 (3.48–4.07)	2.34 (2.16–2.54)
Neurodevelopmental disorders, emotional and behavioral disorders of childhood origin and intellectual disability				
Father only	223 (341.5)	65,294	2.99 (2.62–3.41)	2.03 (1.78–2.32)
Mother only	368 (442.7)	83,131	3.72 (3.36–4.13)	2.59 (2.33–2.87)
Both (different)	178 (501.7)	35,479	5.22 (4.50–6.06)	2.93 (2.52–3.40)
Both (same)	30 (433.1)	6927	6.00 (4.19–8.59)	2.94 (2.06–4.22)
Autism spectrum disorder				
Father only	17 (503.0)	3380	6.26 (3.89–10.08)	3.80 (2.36–6.12)
Mother only	22 (488.6)	4503	6.19 (4.07–9.40)	3.81 (2.51–5.80)
Both (different)	18 (471.1)	3821	7.20 (4.53–11.43)	3.60 (2.27–5.73)
Both (same)	4 (1344.4)	298	20.88 (7.84–55.62)	10.29 (3.86–27.42)
Intellectual disability				
Father only	29 (479.8)	6044	3.76 (2.62–5.42)	2.33 (1.62–3.35)
Mother only	39 (538.1)	7247	4.34 (3.17–5.94)	2.75 (2.01–3.77)
Both (different)	34 (780.6)	4355	7.53 (5.38–10.54)	3.73 (2.66–5.23)
Both (same)	3 (1077.7)	278	11.63 (3.76–35.98)	5.63 (1.82–17.39)
ADHD				
Father only	66 (270.2)	24,430	3.37 (2.64–4.29)	2.01 (1.57–2.56)
Mother only	82 (404.1)	20,293	5.69 (4.57–7.07)	3.46 (2.77–4.30)
Both (different)	64 (333.5)	19,191	4.74 (3.70–6.06)	2.44 (1.90–3.13)
Both (same)	15 (445.7)	3365	8.13 (4.89–13.50)	3.79 (2.28–6.30)
Schizophrenia and other non-mood psychotic disorders				
Father only	53 (237.1)	22,354	1.67 (1.28–2.19)	1.33 (1.02–1.75)
Mother only	77 (300.5)	25,628	2.12 (1.70–2.65)	1.80 (1.44–2.25)
Both (different)	61 (531.7)	11,473	4.41 (3.43–5.67)	2.69 (2.09–3.46)
Both (same)	8 (838.7)	954	6.05 (3.03–12.10)	3.42 (1.71–6.83)
Mood disorders				
Father only	331 (253.5)	130,555	2.07 (1.86–2.31)	1.63 (1.46–1.82)
Mother only	846 (290.7)	291,032	2.58 (2.40–2.76)	2.17 (2.02-2.33)
Both (different)	248 (408.2)	60,755	3.97 (3.50–4.51)	2.50 (2.20–2.84)
Both (same)	97 (479.3)	20,238	5.11 (4.18–6.24)	3.22 (2.63–3.93)
Depression				
Father only	300 (262.6)	114,222	2.19 (1.96–2.46)	1.71 (1.52–1.92)
Mother only	794 (295.5)	268,709	2.64 (2.46–2.84)	2.21 (2.06–2.38)
Both (different)	228 (392.5)	58,087	3.87 (3.40–4.42)	2.43 (2.13–2.77)
Both (same)	83 (484.7)	17,123	5.22 (4.21–6.48)	3.30 (2.65–4.09)
Bipolar				
Father only	27 (187.6)	14,389	1.57 (1.08–2.29)	1.29 (0.88–1.88)
Mother only	74 (287.0)	25,782	2.82 (2.24–3.55)	2.42 (1.93–3.04)
Both (different)	54 (483.1)	11,179	5.33 (4.08–6.96)	3.35 (2.56–4.38)
Both (same)	1 (266.9)	375	3.75 (0.53–26.64)	2.35 (0.33–16.69)
Neurotic/behavioral disorders				
Father only	819 (268.3)	305,280	2.11 (1.97–2.26)	1.69 (1.57–1.81)
Mother only	1926 (302.1)	637,581	2.47 (2.36–2.59)	2.11 (2.01–2.22)
Both (different)	305 (424.6)	71,829	3.91 (3.49–4.38)	2.45 (2.18–2.75)
Both (same)	252 (460.6)	54,717	4.45 (3.93–5.04)	2.82 (2.49–3.21)
Anxiety				
Father only	217 (220.3)	98,486	2.01 (1.75–2.29)	1.58 (1.38–1.81)
Mother only	668 (305.2)	218,862	2.92 (2.70–3.16)	2.42 (2.23–2.62)
Both (different)	231 (413.7)	55,837	4.29 (3.76–4.89)	2.67 (2.34–3.05)
Both (same)	53 (371.7)	14,257	4.47 (3.41–5.86)	2.60 (1.99–3.41)
Obsessive compulsive disorder				
Father only	18 (179.6)	10,023	1.62 (1.02-2.58)	1.43 (0.90–2.27)
Mother only	78 (329.8)	23,654	3.21 (2.57–4.01)	2.83 (2.26–3.53)
Both (different)	39 (557.5)	6996	6.26 (4.57–8.57)	4.09 (2.98–5.60)
Both (same)	1 (685.3)	146	7.77 (1.16–52.23)	5.05 (0.71–35.79)
Stress-related				
Father only	245 (271.1)	90,384	2.14 (1.89–2.43)	1.65 (1.45–1.87)
Mother only	625 (318.7)	196,136	2.62 (2.42–2.84)	2.17 (2.00–2.35)
Both (different)	223 (448.8)	49,688	4.21 (3.68–4.80)	2.60 (2.27–2.97)
Both (same)	38 (424.4)	8955	4.03 (2.93–5.53)	2.56 (1.86–3.53)
Somatoform				
Father only	116 (269.4)	43,061	1.93 (1.61–2.32)	1.72 (1.43–2.06)
Mother only	186 (291.1)	63,898	2.14 (1.86–2.48)	1.86 (1.61–2.15)
Both (different)	66 (542.8)	12,159	4.61 (3.62–5.87)	3.09 (2.42–3.93)
Both (same)	1 (283.1)	353	2.28 (0.32–16.19)	1.78 (0.25–12.61)
Eating disorders				
Father only	29 (229.3)	12,649	1.97 (1.37–2.84)	1.66 (1.15–2.39)
Mother only	301 (261.2)	115,224	2.14 (1.91–2.40)	1.93 (1.72–2.16)
Both (different)	62 (404.4)	15,330	3.98 (3.10–5.11)	2.59 (2.02–3.33)
Both (same)	1 (398.2)	251	4.00 (0.56–28.42)	3.27 (0.46–23.18)
Sleeping disorders				
Father only	28 (242.0)	11,570	2.04 (1.41–2.95)	1.68 (1.16–2.44)
Mother only	41 (275.1)	14,904	2.59 (1.90–3.52)	2.20 (1.62–2.98)
Both (different)	25 (390.4)	6403	4.54 (3.07–6.73)	2.59 (1.74–3.83)
Both (same)	0 (0.0)	172	–	–
Personality disorders				
Father only	134 (362.1)	37,008	2.65 (2.23–3.14)	1.81 (1.53–2.15)
Mother only	358 (507.6)	70,523	4.06 (3.66–4.51)	3.08 (2.77–3.42)
Both (different)	156 (515.0)	30,288	4.57 (3.91–5.35)	2.71 (2.31–3.18)
Both (same)	35 (993.0)	3525	8.53 (6.13–11.88)	4.71 (3.38–6.57)
Substance use				
Father only	676 (264.4)	255,674	1.99 (1.85–2.15)	1.46 (1.35–1.58)
Mother only	579 (281.8)	205,465	2.27 (2.09–2.46)	1.69 (1.55–1.83)
Both (different)	255 (420.0)	60,717	3.86 (3.41–4.37)	2.38 (2.09–2.69)
Both (same)	138 (350.3)	39,391	2.95 (2.49–3.49)	1.62 (1.37–1.92)
Alcohol use				
Father only	500 (256.6)	194,851	1.93 (1.77–2.11)	1.46 (1.34–1.60)
Mother only	395 (254.9)	154,941	2.07 (1.88–2.29)	1.58 (1.43–1.75)
Both (different)	244 (415.8)	58,687	3.72 (3.28–4.22)	2.26 (1.98–2.57)
Both (same)	57 (380.4)	14,984	3.35 (2.58–4.34)	1.90 (1.46–2.47)
Opioid use				
Father only	28 (195.0)	14,359	1.44 (1.00–2.09)	0.97 (0.67–1.40)
Mother only	28 (358.6)	7809	2.81 (1.94–4.07)	1.91 (1.32–2.77)
Both (different)	35 (311.6)	11,232	2.73 (1.96–3.80)	1.49 (1.07–2.08)
Both (same)	10 (333.0)	3003	2.63 (1.42–4.89)	1.41 (0.76–2.62)
Cannabis use				
Father only	56 (241.7)	23,171	1.77 (1.37–2.31)	1.16 (0.90–1.51)
Mother only	25 (285.0)	8771	2.21 (1.49–3.26)	1.39 (0.94–2.06)
Both (different)	52 (353.5)	14,709	3.14 (2.39–4.12)	1.71 (1.30–2.24)
Both (same)	6 (324.7)	1848	2.59 (1.16–5.76)	1.33 (0.60–2.96)
Multiple/unspecific drug use				
Father only	124 (285.1)	43,486	2.19 (1.84–2.61)	1.40 (1.17–1.67)
Mother only	92 (308.9)	29,780	2.45 (1.99–3.00)	1.64 (1.34–2.02)
Both (different)	106 (359.6)	29,479	3.18 (2.62–3.84)	1.74 (1.44–2.11)
Both (same)	29 (343.9)	8433	3.06 (2.12–4.40)	1.53 (1.06–2.20)
Other				
Father only	16 (218.3)	7329	1.63 (1.00–2.66)	1.26 (0.77–2.05)
Mother only	34 (342.3)	9931	2.95 (2.10–4.12)	2.23 (1.59–3.12)
Both (different)	22 (455.9)	4825	4.36 (2.87–6.62)	2.52 (1.66–3.83)
Both (same)	0 (0.0)	142	–	–

ADHD: attention deficit hyperactivity disorder; HR: Hazard ratio; CI: confidence interval; Both (different): one parent had the specified disorder and the other parent had any other psychiatric disorder; Both (same): both parents had the same specific disorder.

Note: HRs with 95% CIs were calculated using Cox regression models. Model 1: Adjusted for birth year by cubic natural splines with 5 knots; Model 2: Additionally adjusted for maternal and paternal education (<9 years primary school, 9 years primary school, 1–2 years secondary school, 3 years secondary school, 1–2 years postgraduate education, ≥3 years postgraduate education, PhD), income (modeled by ranks as natural cubic splines with five degrees of freedom) and age (as natural cubic splines with five degrees of freedom), all defined at delivery. Incidence rate of autism spectrum disorder per 100,000 person years. The reference group of all subgroups was offspring of parents without any psychiatric history.

After accounting for influences by parental age and socioeconomic status, the HRs decreased in magnitude, particularly for risk associated with psychiatric disorders in both parents; fathers only (HR = 1.59, 95% CI = 1.51–1.67), mothers only (HR = 1.95, 95% CI = 1.87–2.04), and both parents (HR = 2.34, 95% CI = 2.16–2.54) (Table 2, Model 2). Results were essentially unchanged after further adjusting for pregnancy-related risk factors (Supplementary eTables S6/S7; Model 3). No evidence for non-proportional hazards (Supplementary eFig. S4).

#### Major psychiatric categories

**Fig. 2:**
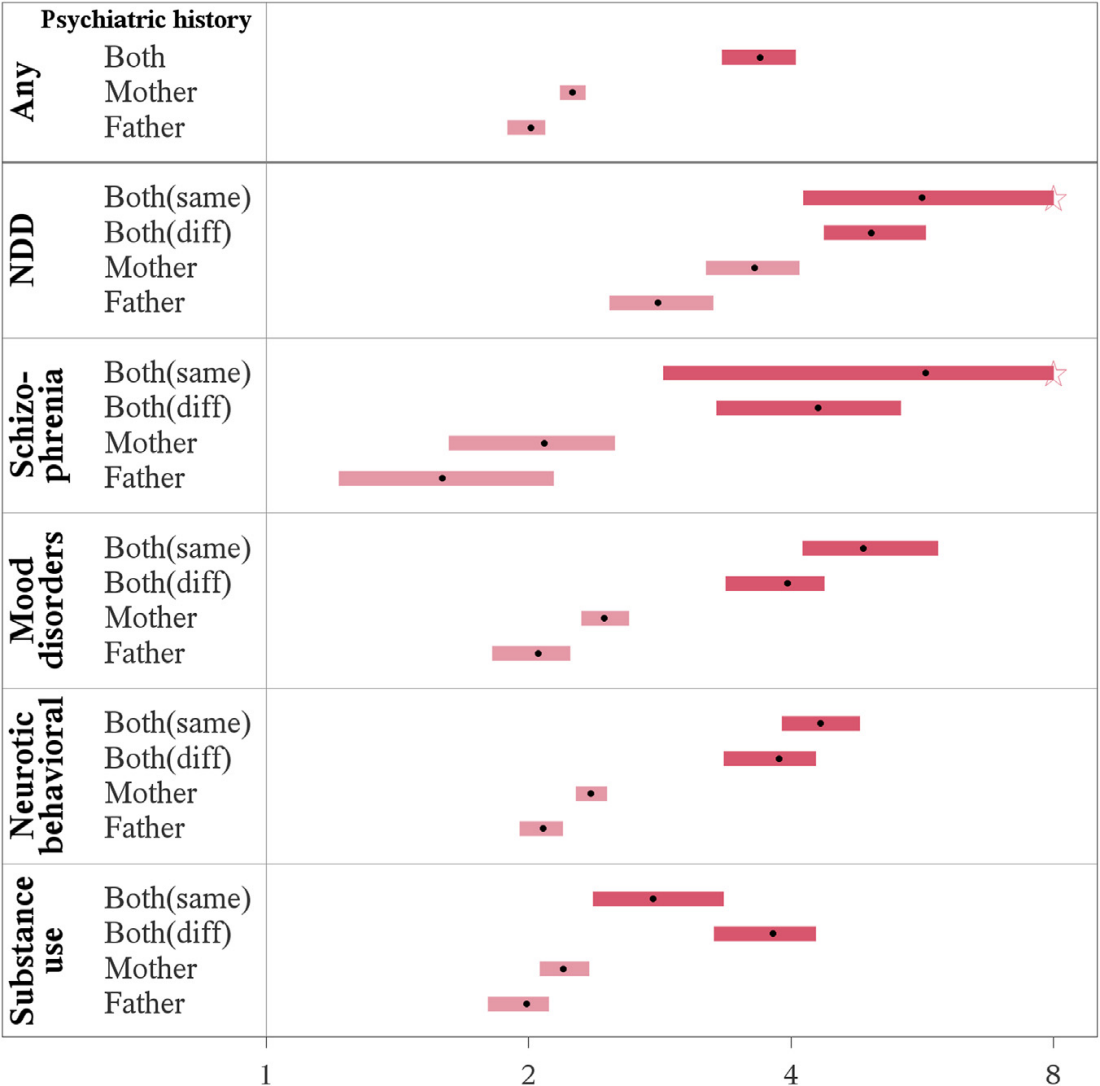
**Autism spectrum disorder in offspring according to psychiatric history in parents, by any psychiatric disorder and by six major categories.** Abbreviations: Father: Father only; Mother: Mothers only; Both (diff): one parent had a psychiatric disorder in one major diagnostic category while the other parent had a condition in another major category; Both (same): both parents had psychiatric disorders in the same major category; NDD: neurodevelopmental disorders, emotional and behavioral disorders of childhood origin and intellectual disability; Schizophrenia: Schizophrenia and other non-mood psychotic disorders; HR: Hazard ratio; CI: confidence interval. Note: The dots in the figure represent HRs and boxes represent 95% CIs. The effect of one parent was present in light pink and of both parents in dark pink. X-axis: HRs. The upper CI was truncated at HR = 8 and marked by a pentagram. HRs with 95% CIs were calculated using Cox regression models, adjusted for birth year by cubic natural splines with 5 knots.

The offspring had a statistically significantly increased risk of autism spectrum disorder for all major categories of parental psychiatric disorders (Table 2, Fig. 2). For NDD category, mood disorders, neurotic/behavior disorders and psychoactive substance use disorders, maternal psychiatric diagnosis was associated with a higher risk of autism spectrum disorder than paternal diagnosis, and a diagnosis in both parents associated with the highest risk (Fig. 2). In contrast, paternal and maternal schizophrenia and other non-mood psychotic disorders increased the offspring risk equally, with the highest risks in case of disorders in both parents (Supplementary eTable S3). When one parent had a psychiatric condition in one major diagnostic category while the other parent had a condition in another major category, the increased risk was comparable to when both parents had conditions in the same category (Table 2; Fig. 2).

#### Specific psychiatric disorders

**Fig. 3:**
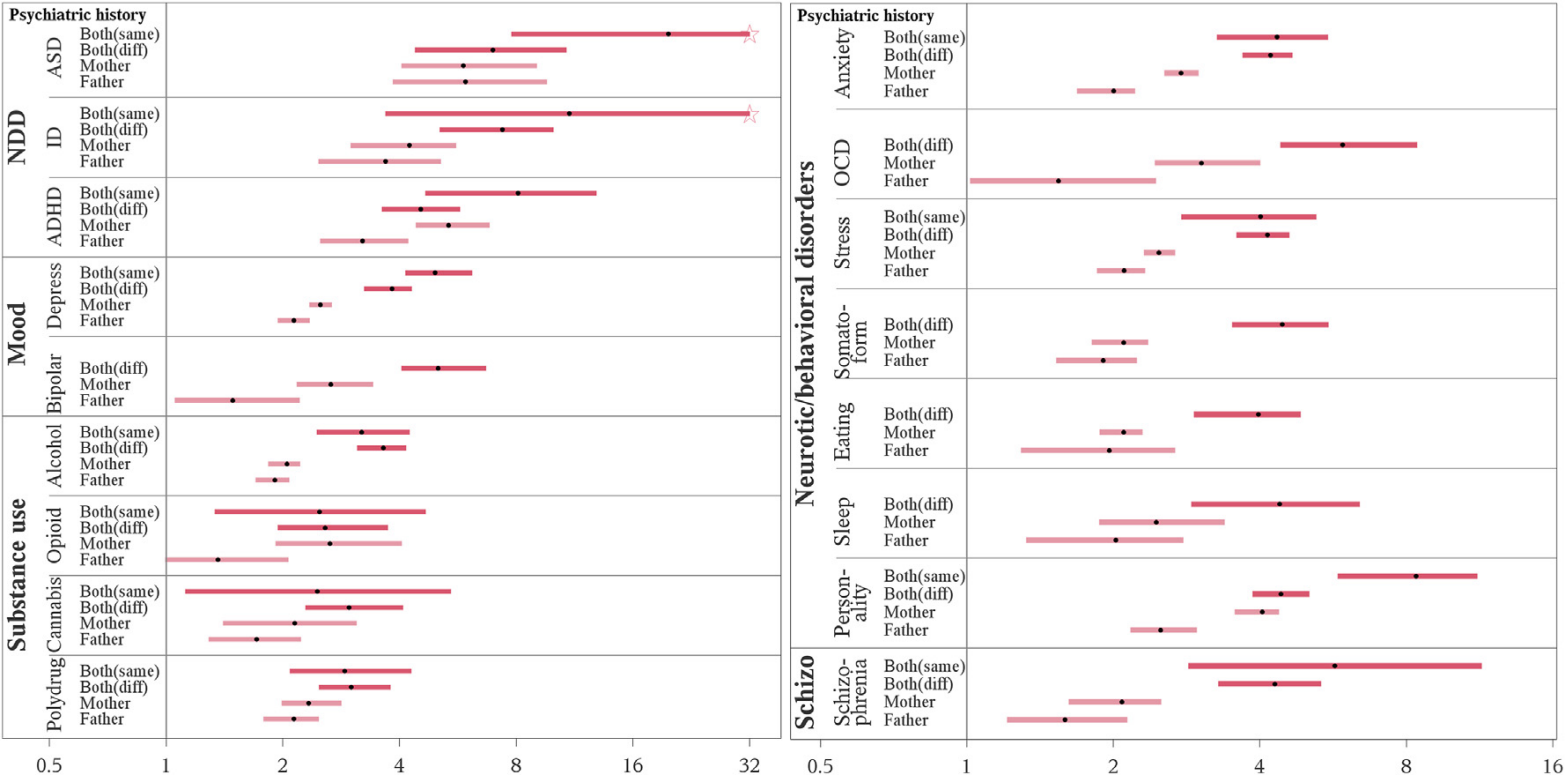
**Autism spectrum disorder in offspring according to psychiatric history in parents, by specific psychiatric disorders.** Abbreviations: Father: Father only; Mother: Mothers only; Both (diff): one parent had the specified disorder and the other parent had any other psychiatric disorder; Both (same): both parents had the same specific disorder; ASD: autism spectrum disorder; NDD: neurodevelopmental disorders, emotional and behavioral disorders of childhood origin and intellectual disability; ID: intellectual disability; ADHD: attention deficit hyperactivity disorder; Mood: mood disorders; OCD: Obsessive Compulsive Disorder; Schizo/Schizophrenia: Schizophrenia and other non-mood psychotic disorders; HR: Hazard ratio; CI: confidence interval. Note: The dots in the figure represent HRs and boxes represent 95% CIs. The effect of one parent was present in light pink and of both parents in dark pink. X-axis: HRs. The upper CI was truncated at HR = 32 and marked by a pentagram. The risk of autism spectrum disorder in the offspring of both parents with bipolar, OCD, somatoform, eating or sleeping disorders was not present in the Figure 3, due to small sample size (<2 cases). HRs with 95% CIs were calculated using Cox regression models, adjusted for birth year by cubic natural splines with 5 knots.

The risk of offspring autism spectrum disorder was, consistently and, statistically significantly increased across all specific psychiatric disorders in fathers only and mothers only (Table 2, Fig. 3). The highest point estimate was observed for disorders under the NDD category, particularly parental autism spectrum disorder; HRs 6.26 (95% CI = 3.89–10.08) for paternal autism spectrum disorder, 6.19 (95% CI = 4.07–9.40) for maternal autism spectrum disorder, 7.20 (95% CI = 4.53–11.43) when one parent had autism spectrum disorder and the other parent had another psychiatric disorder, and 20.88 (95% CI = 7.84–55.62) when both parents had autism spectrum disorder. The was a pattern of higher risk for offspring associated with maternal disorders compared to paternal disorders, and highest risk when both parents had psychiatric disorders, observed for depression, bipolar, anxiety, obsessive-compulsive disorders (OCD), stress-related disorders, and personality disorders (Fig. 3).

### Supplementary analyses

#### Interaction between parental diagnoses

For any psychiatric disorder, mood disorders and neurotic/behavioral disorders, the joint effect of both parents was larger than the expected sum of risks from each parent. However, these effects were smaller than if parental risks were multiplied (Supplementary eTable S4). For substance use disorders, the joint effect of both parents was lower than when parental risks were either summed or multiplied. For NDD category, the joint effect of both parents was lower than when parental risks were multiplied.

#### Sensitivity analyses

The estimated risk of autism spectrum disorder was robust when restricting analyses to parents with psychiatric conditions in only one diagnostic category (Supplementary eTable S8); parents with psychiatric disorders diagnosed prior to one year before conception (Supplementary eTable S9); parents with psychiatric disorders that occurred at least twice lasting more than 30 days (Supplementary eTable S10); autistic disorders as the outcome (Supplementary eTable S11); first-born offspring (Supplementary eTable S12); offspring born 2007–2016 (Supplementary eTable S13); offspring born 1997–2012 (Supplementary eTable S14); singletons (Supplementary eTable S15); offspring without malformations (Supplementary eTable S16); offspring of parents with non-autism spectrum disorder psychiatric disorders (Supplementary eTable S17).

#### Potential pathways of risk

The interaction between parental psychiatric history and preterm delivery was not statistically significant (*p* = 0.70) nor was the interaction between parental psychiatric history and gestational age categories (*p* = 0.27). The HRs of autism spectrum disorder were similar among preterm, early-term and full-term born (Supplementary eTable S18). The mediation analyses showed weak, but statistically significant support for mediation by preterm or early-term birth, and for paternal as well as maternal diagnosis but of small effect size (up to 2% of the overall risk) (Supplementary eTable S19). The risk was similar in male and female offspring (*p* = 0.16 for the interaction between parental psychiatric disorders and offspring sex) (Supplementary eTable S20).

#### Replication cohort in Finland

A total of 1,016,922 children born in Finland were included in the analysis (Supplementary eFig. S2 and eTable S21), contributing 10,289,609 person-years during an average (1st percentile = 0.2 years, 99th percentile = 19.8 years) follow-up of 10 years (1st percentile = 0.2 years, 99th percentile = 19.8 years). Among parents *without* psychiatric disorders, 5954 children had autism spectrum disorder (0.70%; 67 cases per 100,000 person-years). In comparison, 644 children had autism spectrum disorder (0.95%; 106 cases per 100,000 person-years) if a psychiatric history in fathers only, 767 children had autism spectrum disorder (0.96%; 130 cases per 100,000 person-years) if a psychiatric history in mothers only, and 283 children had autism spectrum disorder (1.45%; 216 cases per 100,000 person-years) if both parents were affected.

In comparison to undiagnosed parents, the risk of autism spectrum disorder was increased in offspring of parents with psychiatric disorders, including in fathers only (HR = 1.63, 95% CI = 1.50–1.77), mothers only (HR = 2.12, 95% CI = 1.96–2.28), and both parents (HR = 3.61, 95% CI = 3.20–4.07) (Supplementary eTable S22). The combined risk estimates for autism spectrum disorder in Swedish and Finnish populations are also presented in Supplementary eTable S22. Additional psychiatric disorders in a parent further increased the risk of autism spectrum disorder; for fathers, one diagnostic category (HR = 1.52, 95% CI = 1.40–1.65), two categories (HR = 1.88, 95% CI = 1.64–2.16), and ≥ three categories (HR = 2.34, 95% CI = 1.91–2.87). Results were aligned with additional psychiatric diagnoses in mothers; HRs for one, two, and ≥ three diagnostic categories respectively: 1.95 (95% CI = 1.80–2.11), 2.50 (95% CI = 2.21–2.82) and 2.78 (95% CI = 2.31–3.35) (Supplementary eFig. S5 and eTable S23). The risk of autism spectrum disorder was increased for all major categories of parental psychiatric disorders (Supplementary eTable S22). Maternal disorders were associated with a higher risk of autism spectrum disorder than paternal disorders, and a disorder in both parents was associated with the highest risk, for any psychiatric disorder, mood disorders and neurotic/behavior disorders; with non-overlapping CIs.

## Discussion

To our knowledge, this is the most comprehensive study to date examining the association between parental psychiatric disorders and the risk of autism spectrum disorder in offspring. Our findings show that 20% of children with autism spectrum disorder had at least one parent with psychiatric disorders. The risk of autism spectrum disorder was higher in offspring of parents with psychiatric disorders, particularly when the mother had a disorder. The highest risk was observed when both parents had a disorder. The results further indicated that the risk of autism spectrum disorder in offspring increased with each additional co-occurring psychiatric disorder, in both mothers and fathers. It is important to note that offspring autism spectrum disorder was not only associated with parental autism spectrum disorder, but with all parental psychiatric disorders.

Psychiatric disorders are among the most heritable conditions and their genetic architecture is considered to be polygenic.^9^^,^^10^ For example, autism spectrum disorder shares common susceptibility variants with Attention-Deficit/Hyperactivity Disorder (ADHD), schizophrenia, depression and bipolar disorders.^7^ OCD, anxiety and substance use disorders may also be linked to autism spectrum disorder through genetic overlap with schizophrenia, depression and bipolar disorders.^7^^,^^36^ The results of genetic research demonstrate wide genetic overlap between psychiatric disorders. This is reflected in our findings of a strong associations between offspring autism spectrum disorder and parental psychiatric history across the psychiatric spectrum, with the greatest risk when both parents were affected and when multiple parental psychiatric conditions were present across diagnostic categories.

The findings were separately validated in the Finnish population. As also shown in other publications,^37^ the prevalence of autism spectrum disorder was somewhat lower in Finland compared with Sweden. Nevertheless, the relative risk and risk pattern of autism spectrum disorder associated with parental psychiatric disorders are consistent across the two countries.

Limited research has explored how maternal and paternal psychiatric disorders, separately, link to offspring autism spectrum disorder (Supplementary eTable S1).^11^, ^12^, ^13^, ^14^, ^15^, ^16^ A general association has been suggested for any psychiatric disorders in parents and an increased risk of autism spectrum disorder, but detailed analyses of specific disorders are often lacking and results are inconsistent between disorders. Importantly, it largely remains unknown where both parents had a mental illness and whether several different psychiatric disorders occurred in a parent. There were also methodological limitations and insufficient statistical precision. The present study addresses these shortcomings and provides robust evidence to previously inconclusive results. There was a general pattern of higher risk of autism spectrum disorder from maternal exposure than paternal exposure to psychopathology, and the highest risk occurred when both parents were affected. This pattern is particularly evident in parental mood disorders and neurological/behavioral disorders, with consistent results in Sweden and Finland. Mechanisms may include overlapping genetic risk, intrauterine factors, maternal behavior, nonrandom mating, and gene–environment interactions, all of which warrant future studies.

The pattern of risk in the overall population may inform theories about underlying causes and mechanisms. For example, a pattern where the combined effect of maternal and paternal psychiatric illness is similar to the sum of the parental risks would be compatible with inheritance of common genetic variants from the parents. A pattern where the combined effect is similar to the product of the risk from the two parents would be compatible with genetic epistasis. In general, the magnitude of the combined effect of parental diagnosis was between the sum and the multiplication of parental risks. However, for substance use disorders, the joint effect was lower than either the sum or multiplication of parental risks.

For pathways of risk, environmental/social factors may play a role in the association between parental psychiatric disorders and offspring autism spectrum disorder in Sweden and Finland. Also, there was support for a small mediating effect through preterm/early-term birth. This provides some support for the earlier hypothesis that increased liability for psychiatric disorders may contribute to abnormal brain development in infants subject to preterm stress.^38^ Although genetic risk factors play a major role in the development of autism spectrum disorder, understanding the underlying etiology of autism spectrum disorder requires consideration of contributions also from environmental (non-genetic) risk factors as well as the complex interactions between environment and genetics.^5^^,^^39^ For example, experiencing a psychiatric condition (either directly, or via a partner’s mental health) during pregnancy can increase psychological stress, and prenatal exposure to elevated maternal stress has been associated with increased risk of autism spectrum disorder, and variability in autism-like traits in the general population.^40^ The lower risk of autism spectrum disorder from paternal exposure, compared with maternal exposure, may also indicate a risk influenced by the interaction between genetic susceptibility and environmental exposures. Future studies could explore potential environmental factors that either contribute directly to the development of autism spectrum disorder, or interact with underlying genetic predispositions.

Our study was able to handle biases in many important ways. Results from this study were derived from a birth cohort with nationwide coverage, and virtually complete follow up, in a publicly financed and utilized health system minimizes selection biases. Using prospectively clinically ascertained psychiatric diagnoses we avoid recall biases common in retrospective studies. The rich database allowed us to adjust for biases from potential confounding factors, and to apply Cox regression models, a survival-analysis technique which adjust for differences in length of follow-up; yet another potential source for biases. By restricting the analyses to diagnoses before the child's birth, we further avoid any reverse causation that could occur if diagnoses after the child's autism diagnosis had been included. The results were robustly replicated in a Finnish sample, strengthening the reliability and generalizability of the results. The large sample size and rich database provided analytical power to precisely examine how the risk of autism spectrum disorder in offspring varies and relates to cases of psychiatric disorders in mothers and fathers alone, in both parents together, as well as across multiple specific psychiatric disorders. As a consequence of our large sample size, we provide estimates of psychiatric diagnoses prior to childbirth for mother, father and both parents, which can be compared with the wider CI’s in earlier studies.^11^, ^12^, ^13^, ^14^^,^^16^ The individual-level risk estimates can provide insights into the complex risk structure underpinning the association between parental psychiatric disorders and offspring autism spectrum disorder, and can potentially be used by physicians and public health workers, combined with other risk factors, to provide risk estimates for autism spectrum disorder and in consulting to families.

The study has several limitations. Despite the large sample size, statistical precision was limited in specific parental psychiatric disorders, particularly with respect to a diagnosis in both parents. We did not have information allowing us to distinguish between children raised by non-biological parents. However, only a small proportion (<1%) of children in Sweden are raised by non-biological parents (https://www.socialstyrelsen.se/globalassets/sharepoint-dokument/artikelkatalog/statistik/2022-9-8096.pdf).^41^ Hence, we believe they have little impact on our overall results. We did not adjust to other parental conditions potentially confounding the association between parental mental illness and offspring autism risk. Data on psychiatric disorders diagnosed from primary care visits were not available, and we were more likely to capture cases at the more severe end of the spectrum of disorders. As with any register study, we are only able to study individuals who seek and receive care, which may limit the generalizability of the findings to those already in the care system. Therefore, we can only speculate about the true relationship between psychiatric conditions in parents and offspring autism. The study only included parents born in the Nordic countries, so the results may not be generalizable to populations in other regions. We did not have data on medical or other types of treatment which may impact severity of the condition. However, antidepressant treatment during pregnancy,^42^ and during the conception period,^43^ do not appear to be causally associated with an increased risk of autism spectrum disorder in two large population-based studies. Future studies should address the potential impact of other psychotropic medications on offspring risks. Finally, there is a lack of information regarding potential environmental exposures during pregnancy, such as prenatal stress, diet, pollution, and medication.

In our study, 20% of children with autism spectrum disorder had at least one parent with psychiatric disorders. Psychiatric disorders in both parents conveyed the highest risk of offspring autism spectrum disorder, followed by mothers only and then fathers only. The risk increased with number of co-occurring disorders. All parental psychiatric disorders were associated with increased risk of autism spectrum disorder. Finding suggests that a comprehensive evaluation of family history that incorporates the full range of parental psychiatric disorders is necessary in assessing the risk of autism spectrum disorder, rather than solely focusing on familial autism spectrum disorder. These findings will help to identify children at high risk for autism spectrum disorder.

## Contributors

SS is the PI of this study. WY, AR and SS conceived the study. WY and AP conducted the analysis. WY, AP, AR, AK, JFL, KR, MLP, MP, MES, UÅ, EK and SS provided substantial scientific input in interpreting the results. WY wrote the draft of the manuscript. WY, AP, AR, AK, JFL, KR, MLP, MP, MES, UÅ, EK and SS made critical revision to the draft. The corresponding author attests that all listed authors meet authorship criteria and that no others meeting the criteria have been omitted.

## Data sharing statement

Data cannot be shared publicly because of restrictions by law. Access to the Swedish data can be requested from the Swedish registers (socialstyrelsen@socialstyrelsen.se). Access to the Finnish data can be requested from Finnish Social and Health Data Permit Authority Findata (info@findata.fi).

## Declaration of interests

Dr. Yin reports funding from KI Research Foundation Grant 2022–02021.

Dr. Kolevzon reports support from American Psychiatric Publishing, Phelan-McDermid Syndrome Foundation – Spain and Phelan-McDermid Syndrome Foundation – Brazil, consulting fees from RitrovaTherapeutics, CureSHANK, Aelis Farma, Acadia, Alkermes, Jaguar Therapeutics, GW Pharmaceuticals, Neuren Pharmaceuticals, Clinilabs Drug Development Corporation, Scioto Biosciences, Biogen, PYC Therapeutics, stock or stock options from Ovid Therapeutics, and payment for expert testimony from Anthone v Franciscan Health System (Deposition) FAVROS LAW 701 Fifth Avenue, Suite 4750, Seattle, WA 98104, Ting v. Christina Ring, MD (Deposition) CARDONE & RUTBERG, PC, 3773 Cherry Creek North Drive, Suite 680W, Denver, Colorado, 80,209, Palmquist v. Hain Celestial Group (Trial only), COVINGTON & BURLING LLP, One CityCenter, 850 Tenth Street, NW, Washington, DC 20001–4956, Anderson v. The Johns Hopkins System (Deposition), GOODELL, DEVRIES, LEECH, & DANN, LLP, One South Street, 20th Floor, Baltimore, MD, and Acetaminophen – ASD-ADHD Product Liability Litigation (Deposition), BUTLER SNOW LLP, 810 7th Avenue, Suite 1105, New York, NY, 10,019. Dr. Kolevzon also reports participation in Vagal nerve stimulation in eating and weight disorders.

Dr. Ludvigsson has coordinated an unrelated study on behalf of the Swedish IBD quality register (SWIBREG). That study received funding from Janssen corporation. Dr Ludvigsson has also received financial support from MSD developing a paper reviewing national healthcare registers in China. Dr Ludvigsson has an ongoing research collaboration on celiac disease with Takeda.

Dr. Kajantie reports support from Academy of Finland, European Commission, Sigrid Jusélius Foundation, Foundation for Pediatric Research, Signe and Ane Gyllenberg Foundation, Yrjö Jahnsson Foundation, Novo Nordisk Foundation, Jalmari and Rauha Ahokas Foundation and Finnish Medical Foundation.

Dr. Sandin reports funding from Swedish Research council Grant #2021–0214 and payment for acting as opponent at PhD defense in University of Oslo, Norway. Dr Sandin is a member of DSMB for Vagus Nerve Stimulation for Treatment of Eating Disorders at Mount Sinai Hospital, New York STUDY-21-01790.

All authors confirm the independence of researchers from funders.

**Transparency statement**

The guarantor of this study affirms that this manuscript is an honest, accurate, and transparent account of the study being reported; that no important aspects of the study have been omitted; and that any discrepancies from the study as planned (and, if relevant, registered) have been explained.

**Ethical approval**

The study was approved by the national Swedish ethics review board, Sweden (2017/1875–31/1; 2018/1864–32; 2019–06314) and the Institutional review board of The Finnish Institute for Health and Welfare (THL/1984/6.02.01/2018 and THL/4309/6.02.01/2022). No individual level consent was required, and all data used were anonymized.

**Patient and public involvement statement**

Patients or the public were not involved in the design, conduct, or reporting, or dissemination of our research.

**Availability of data and material**

Data cannot be shared publicly because of restrictions by law. Access to the Swedish data can be requested from the Swedish registers (https://www.socialstyrelsen.se/en/statistics-and-data/registers/; socialstyrelsen@socialstyrelsen.se). Access to the Finnish data can be requested from Finnish Social and Health Data Permit Authority Findata (info@findata.fi).

## Appendix ASupplementary data

Supplementary eMethod S1, eFigs. S1–S5, and eTables S1–S23

SAP
