# Supplementary material for: Association between parental psychiatric disorders and risk of offspring autism spectrum disorder: a Swedish and Finnish population-based cohort study
Source: Lancet Reg Health Eur. 2024 Apr 23;40:100902. doi: 10.1016/j.lanepe.2024.100902 (PMC11059471; doi:10.1016/j.lanepe.2024.100902)
Supplement: Supplementary eMethod S1, eFigs. S1–S5, and eTables S1–S23 [file mmc1.pdf]

Supplementary Methods, Tables and Figures

List of contents

eMethods

eMethod 1 Categories of parental psychiatric disorders 2

eTables

eTable 1 Previous population-based studies on parental psychiatric disorders and offspring ASD 3
eTable 2 ICD codes for psychiatric disorders 6
eTable 3 Psychiatric disorders in parents and risk of offspring ASD, pairwise comparisons 8
eTable 4 Interaction between paternal and maternal psychiatric disorders 11
eTable 5 Number of co-occurring psychiatric disorders in mothers and fathers and risk of ASD in the offspring 12
eTable 6 Offspring of mothers with no missing data on smoking and BMI during pregnancy 13
eTable 7 Parental psychiatric disorders and risk of offspring ASD, adjusted for pregnancy-related risk factors 14
eTable 8 Parental psychiatric disorders and risk of offspring ASD among parents with only one psychiatric category 17
eTable 9 Parental psychiatric disorders first diagnosed prior to one year before conception and risk of ASD 18
eTable 10 Parental psychiatric disorders occurred at least twice with an interval of more than 30 days 19
eTable 11 Parental psychiatric disorders and risk of autistic disorder in offspring 20
eTable 12 Parental psychiatric disorders and risk of ASD among first born offspring 21
eTable 13 Parental psychiatric disorders and risk of ASD among offspring born 2007-2016 22
eTable 14 Parental psychiatric disorders and risk of ASD among offspring born 1997-2012 23
eTable 15 Parental psychiatric disorders and risk of ASD among singletons 24
eTable 16 Parental psychiatric disorders and risk of ASD among offspring without malformation 25
eTable 17 Any non-ASD psychiatric disorders in parents and risk of ASD in offspring 26
eTable 18 Parental psychiatric disorders and risk of offspring ASD by gestational age categories 27
eTable 19 Mediation analysis of the association between parental psychiatric disorders and offspring ASD by preterm and early term birth 28
eTable 20 Parental psychiatric disorders and offspring ASD risk by offspring sex 29
eTable 21 Cohort characteristics by any psychiatric disorder in parents in Finland 30
eTable 22 Parental psychiatric disorders before childbirth and risk of offspring ASD in Finland, and across sites 31
eTable 23 Number of co-occurring psychiatric disorders in mothers and fathers and risk of ASD in the offspring in Finland 32

eFigures

eFigure 1 Examples of risk patterns of parental psychiatric disorders on offspring ASD risk 33
eFigure 2 Flow diagram illustrating the identification of the study cohort 34
eFigure 3 Adjusted Inverse Kaplan-Meier curves for ASD by co-occurring psychiatric disorders in parents 35
eFigure 4 Scaled Schoenfeld residual plot to assess proportional hazards assumption 36
eFigure 5 Inverse Kaplan-Meier curves for ASD by co-occurring psychiatric disorders in parents in Finland 37

eMethod 1 Categories of parental psychiatric disorders

We used a top-down perspective from broad/major categories to narrow/specific diagnosis to categorize analysis groups. First, all psychiatric disorders were included as an overall measure, 'any psychiatric disorder'. Next, disorders were divided into six major diagnostic categories (neurodevelopmental disorders, emotional and behavioral disorders of childhood origin and intellectual disability (NDD); schizophrenia and non-mood psychotic disorders; mood disorders; neurotic/behavioral disorders; psychoactive substance use; other/unspecific psychiatric disorders). Finally, disorders were dived into sixteen specific disorders under different categories (Autism Spectrum Disorders (ASD), intellectual disability (ID) and attention deficit hyperactivity disorder (ADHD) under NDD; depression and bipolar under mood disorders; anxiety, obsessive-compulsive disorder (OCD), stress-related disorders, somatoform, eating disorders, sleeping disorders and personality disorders under neurotic/behavioral disorders; alcohol use, opioid type use, cannabis use and multiple drug /unspecific drug use under psychoactive substance use). In our algorithms, one individual can contribute to several psychiatric categories and specific disorders. Only few couples are observed with exactly the same psychiatric disorder. Thus, in examining the effect of a psychiatric disorder in both parents, we created categories of maternal (or paternal) exposure to a certain disorder and paternal (or maternal) exposure simultaneously to any other psychiatric disorder, i.e., a specific disorder in fathers only, a specific disorder in mothers only, a specific disorder in one parent and any other psychiatric disorder in the other parent, and the same specific disorder in both parents.

eTable 1 Previous population-based studies on parental psychiatric disorders and offspring ASD

eTable 1 (A) A brief summary of what has been done before

Study	Population	ASD last diagnosis	ASD cases	Psychiatric history in both parents	Interaction between parental diagnosis	Diagnostic categories	Specific disorders	Co-occurring disorders	Diagnosis before childbirth
1	Denmark	2001	818	N	N	N	N	N	Y
2	Sweden	2003	1,227	Y#	N	Y	Y	N	N*
3	Finland	2007	4,713	Y#	N	Y	N	N	N*
4	Sweden	NA	7,236	N	N	N	N	N	N*
5	Taiwan	2017	24,279	N	N	Y	Y	N	Y§
6	Taiwan	2015	8,933	N	N	Y	Y	N	Y

Abbreviations. ASD: Autism Spectrum Disorders; Y: what has been done before (Yes);

N: what has not been done yet (No).

Note: Studies included in this table estimated risk of offspring ASD for any psychiatric disorder in mothers and fathers separately. In addition, there are two studies that did not separate mothers and fathers [7,8]. # Only for any psychiatric diagnosis; * In sensitivity analysis separated any parental diagnosis before and after childbirth, but unable to restrict to parental diagnosis before childbirth for main results due to insufficient statistical power. § Some parental psychiatric disorders were restricted to before childbirth but not all (including schizophrenia, bipolar, depression, anxiety, adjustment disorders and substance use).

eTable 1 (continued)

eTable 1 (B) A detailed summary of what has been done in previous studies

Study	Participates	Psychiatric disorders included	Specific disorders	Assocation with parental psychiatric disorders	Both	Co-occurrence	Timing
Lauritsen MB et al. [1]	Danish register. Children born 1984-1998	Schizophrenia and other paranoid psychoses, mood disorders, and nervous conditions and personality disorders.	No category or specific disorder.	Associated with any maternal diagnosis, not paternal.	No	No	Prior to the child's birth
Julie L Daniels et al [2]	Swedish register. Children born 1977-2003. Matched case control.	Schizophrenia, other non-affective psychoses, affective disorders, neurotic and personality disorders and other nonpsychotic disorders, alcohol and drug addiction and abuse and autism.	By category. Specific disorder incl depression and ASD.	Any in one and both parents. Maternal depression, neurotic, personality and other nonpsychotic disorder; null for paternal.	Any	No	Any time, including parental diagnosis after offspring ASD. In secondary analysis checked any parental diagnosis before childbirth
Elina Jokiranta [3]	Finish registers Children born 1987-2005.	Schizophrenia spectrum; affective disorders; neurotic and personality disorders and other nonpsychotic disorders; alcohol and drug addiction/abuse	By category. No specific disorder.	All categories in mothers. Most categories except for alcohol/drug use in fathers. Both not statistically higher than one parent	Any	No	Mixed timing in main results. In sensitivity analysis checked any parental diagnosis before birth.
McCoy BM et al. [4]	Sweden register. Children born1992-2001.	Maternal and paternal schizophrenia, bipolar disorder, or another non-organic psychosis.	No category or specific disorders.	Any psychiatric disorder in mothers and fathers.	No	No.	Mixed timing in main results. In sensitivity analysis checked any parental diagnosis before birth
Yi-Ling Chien [5]	Taiwan register. Children born 2004-2017. Matched case-controls	For diagnoses before childbirth, including schizophrenia, bipolar, depression, anxiety, adjustment disorders, substance. *	Listed specific disorders.	Associated with all listed maternal disorders and paternal schizophrenia	No.	No	Before birth in analysis for schizophrenia, bipolar, depression, anxiety, adjustment disorders, substance.
Yu T[6]	Taiwan registers. Children born 2004-2008.	Substance-related disorders, schizophrenic and psychotic disorders, mood disorders, anxiety disorders, and personality disorders; bipolar disorders, depressive disorders, OCD, anxiety states & phobic disorders	By categories (not included all neurotic/behavioral disorders). Listed specific disorders as listed.	Associated with paternal schizophrenic & psychotic, anxiety disorders. Maternal schizophrenic & psychotic, mood, bipolar, depression, anxiety, personality disorders. Authors concluded that risk estimates seemed larger for mothers, but with overlapping CIs.	No	No	Before birth

Abbreviations. ASD: Autism Spectrum Disorders; ID: intellectual disability; ADHD: attention deficit hyperactivity disorder; OCD: Obsessive Compulsive Disorder. * In Chien's study, parental diagnosis of OCD, ASD and ADHD after childbirth and child ASD were included, however lacking offspring risk for parental diagnosis before childbirth.

eTable 1 (continued)

Comparisons of previous studies and the present study, to our best knowledge

Only a few studies have separately examined maternal and paternal associations for several psychiatric disorders**.** In 2008, a Swedish register-based study [2] found that neurotic/personality disorder and other nonpsychotic disorders and depression of the mother, but not the father, were associated with increased ASD risk in offspring. Null associations were reported for schizophrenia, affective disorders, alcohol use disorders and ASD. Later, a Finish register study [3] reported associations for major diagnostic categories in parents but no specific disorders were examined. The two studies, however mixed parental disorders before and after delivery in primary analysis. Two Taiwan population-based studies examined the association parental psychiatric history and offspring ASD using the national health database [5,6]. One of the two studies [5] suggested broad associations with that maternal psychiatric disorders and an association with paternal schizophrenia. Another study [6] suggested broad associations with mothers and fathers, but null association for substance use disorders. The two studies examined several specific disorders but not the full range of psychiatric disorder (e.g., NDD and some neurotic/behavior disorders like stress-related disorders, sleeping disorders, eating disorders, etc.). The studies claim that the effect of maternal disorders was greater than that of paternal disorders, however, with overlapping CIs and without other statistical tests. In addition, large remains unknown where both parents had a mental illness and where several different psychiatric disorders occurred in a parent.

References:

1. Lauritsen MB, Pedersen CB, Mortensen PB. Effects of familial risk factors and place of birth on the risk of autism: a nationwide register-based study. J Child Psychol Psychiatry. Sep 2005;46(9):963-71. doi:10.1111/j.1469-7610.2004.00391.x

2. Daniels JL, Forssen U, Hultman CM, et al. Parental psychiatric disorders associated with autism spectrum disorders in the offspring. Pediatrics. May 2008;121(5):e1357-62. doi:10.1542/peds.2007-2296

3. Jokiranta E, Brown AS, Heinimaa M, Cheslack-Postava K, Suominen A, Sourander A. Parental psychiatric disorders and autism spectrum disorders. Psychiatry Res. May 30 2013;207(3):203-11. doi:10.1016/j.psychres.2013.01.005

4. McCoy BM, Rickert ME, Class QA, Larsson H, Lichtenstein P, D'Onofrio BM. Mediators of the association between parental severe mental illness and offspring neurodevelopmental problems. Ann Epidemiol. Sep 2014;24(9):629-34, 634.e1. doi:10.1016/j.annepidem.2014.05.010

5. Chien YL, Wu CS, Chang YC, Cheong ML, Yao TC, Tsai HJ. Associations between parental psychiatric disorders and autism spectrum disorder in the offspring. Autism Res. Oct 17 2022;doi:10.1002/aur.2835

6. Yu T, Chang KC, Kuo PL. Paternal and maternal psychiatric disorders associated with offspring autism spectrum disorders: A case-control study. J Psychiatr Res. Jul 2022;151:469-475. doi:10.1016/j.jpsychires.2022.05.009

7. Larsson HJ, Eaton WW, Madsen KM, et al. Risk factors for autism: perinatal factors, parental psychiatric history, and socioeconomic status. Am J Epidemiol. May 15 2005;161(10):916-25; discussion 926-8. doi:10.1093/aje/kwi123

8. Wang HE, Cheng CM, Bai YM, et al. Familial coaggregation of major psychiatric disorders in first-degree relatives of individuals with autism spectrum disorder: a nationwide population-based study. Psychol Med. Jun 2022;52(8):1437-1447. doi:10.1017/s0033291720003207

eTable 2 ICD codes for psychiatric disorders

eTable 2A ICD codes (Swedish version) for psychiatric disorders

Disorder	ICD-10	ICD-9	ICD-8
Overall mental illness	F10-F99	291, 295, 296, 297, 298, 299, 300, 301, 302, 303, 304, 305, 306, 307, 308, 309, 311, 312, 313, 314, 315, 316, 317, 318, 319	291, 295, 296, 297, 298, 299, 300, 301, 302, 303, 304, 305, 306, 307, 308, 310, 311, 312, 313, 314, 315
**Psychoactive substance use**	F10-F19	291, 303, 304, 305	291, 303, 304
Alcohol use	F10	291, 303, 305A	291, 303
Opioid type use	F11	304A	304.0, 304.1
Cannabis use	F12	304D	304.5
Multiple drug /unspecific drug use	F19	304H, 304W, 304X	304.8, 304.9
**Schizophrenia and other non-mood psychotic disorder**	F20–29	295, 297, 298C-X	295, 297, 298.2, 298.3, 298.9
**Mood disorders**	F30-39	296, 298A, 298B, 300E, 311, 301B	296, 298.0, 298.1, 300.4, 301.1
Depression	F32, F33, F341, F348, F349	296B, 300E, 311, 301B	296.0, 298.0, 300.4, 301.1
Severe depression	F32.2, F32.3, F33.2, F33.3	-	-
Bipolar	F30, F31, F340	296C, 296D, 296E	296.1, 296.2, 296.3, 296.8
**Neurotic/behavioral disorders**	F40-F48, F50-59, F60-F69	300–302 (excl 300E, 301B), 306, 307, 308, 309	300–302 (excl. 300.4, 301.1), 305, 306, 307
Anxiety	F40 F41	300A, 300C	300.0, 300.2
OCD	F42	300D	300.3
Stress-related disorder	F43	308, 309	
Somatoform disorder	F45	306	305, 306 (excl 306.5, 306.4)
Eating disorder	F50	307B, 307F	306.5
Sleeping disorder	F51	307E	306.4
Personality disorder	F6	301A, 301C-301DEFGHJWX	301.0, 301.2-301.9
**NDD**	F70-F79, F80-F89, F90-98	299, 312-315, 317-319	308, 310-315
ID	F70, F71, F72, F73, F78, F79	317, 318, 319	310, 311, 312, 313, 314, 315
ASD	F840, F841, F843, F845, F848, F849	299A	
AD	F840	299A	
Asperger’s syndrome	F845		
Other ASD	F841, F848, F849		
ADHD	F90	314	
**Other/unspecific psychiatric disorders**	F99	316	299

eTable 2B ICD codes (Finnish version) for psychiatric disorders

Disorder	ICD-10	ICD-9	ICD-8
Overall mental illness	F10-F99	291, 295, 296, 297, 298, 299, 300, 301, 302, 303, 304, 305, 307, 309, 312, 313, 314, 315, 316, 317, 318, 319	291, 295, 296, 297, 298, 299, 300, 301, 302, 303, 304, 305, 306, 307, 308, 310, 311, 312, 313, 314, 315
**Psychoactive substance use**	F10-F19	291, 303, 304, 305	291, 303, 304
**Schizophrenia and other non-mood psychotic disorder**	F20–29	295, 297, 2989	295, 297, 2982, 2983, 2989,
**Mood disorders**	F30-39	296, 2988, 3004, 3011	296, 2980, 2981, 3004, 3011
**Neurotic/behavioral disorders**	F40-F48, F50-59, F60-F69	300–302 (excl 3004,3011), 307, 309	300–302 (excl. 3004,3011), 305, 306, 307
**NDD**	F70-F79, F80-F89, F90-98	299, 312-315, 317-319	308, 310-315
ASD	F840, F841, F843, F845, F848, F849	2990	
**Other/unspecific psychiatric disorders**	F99	316	299

Abbreviations. ICD: International Classification of Diseases. NDD: Neurodevelopmental disorders, emotional and behavioral disorders of childhood origin and intellectual disability; ASD: autism spectrum disorders; ID: intellectual disability; AD: Autistic Disorder; ADHD: attention deficit hyperactivity disorder; OCD: Obsessive Compulsive Disorder. Main or secondary diagnoses were registered in the *Swedish National Patient Register.*

eTable 3 Psychiatric disorders in parents and risk of offspring ASD, pairwise comparisons

**Analysis group**	**Model 1 HR (95% CI)**
**Any psychiatric disorder**	
Paternal only vs maternal only	0.86 (0.81-0.92)
Paternal only vs both	0.54 (0.49-0.59)
Maternal only vs both	0.62 (0.57-0.68)
**NDD**	
Paternal only vs maternal only	0.80 (0.68-0.95)
Paternal only vs both (different)	0.57 (0.47-0.70)
Paternal only vs both the same category	0.50 (0.34-0.73)
Maternal only vs both (different)	0.71 (0.60-0.85)
Maternal only vs both the same category	0.62 (0.43-0.90)
**ASD**	
Paternal only vs maternal only	1.01 (0.54-1.91)
Paternal only vs both (different)	0.87 (0.45-1.69)
Paternal only vs both the same disorder	0.30 (0.10-0.89)
Maternal only vs both (different)	0.86 (0.46-1.60)
Maternal only vs both the same disorder	0.30 (0.10-0.86)
**ID**	
Paternal only vs maternal only	0.87 (0.54-1.40)
Paternal only vs both (different)	0.50 (0.30-0.82)
Paternal only vs both the same disorder	0.32 (0.10-1.06)
Maternal only vs both (different)	0.58 (0.36-0.91)
Maternal only vs both the same disorder	0.37 (0.12-1.20)
**ADHD**	
Paternal only vs maternal only	0.59 (0.43-0.82)
Paternal only vs both (different)	0.71 (0.50-1.00)
Paternal only vs both the same disorder	0.41 (0.24-0.73)
Maternal only vs both (different)	1.20 (0.87-1.67)
Maternal only vs both the same disorder	0.70 (0.40-1.21)
**Schizophrenia and other non-mood psychotic disorder**	
Paternal only vs maternal only	0.79 (0.56-1.12)
Paternal only vs both (different)	0.38 (0.26-0.55)
Paternal only vs both the same category	0.28 (0.13-0.58)
Maternal only vs both (different)	0.48 (0.34-0.67)
Maternal only vs both the same category	0.35 (0.17-0.73)
**Mood disorders**	
Paternal only vs maternal only	0.81 (0.71-0.91)
Paternal only vs both (different)	0.52 (0.44-0.62)
Paternal only vs both the same category	0.41 (0.32-0.51)
Maternal only vs both (different)	0.65 (0.56-0.75)
Maternal only vs both the same category	0.50 (0.41-0.62)
**Depression**	
Paternal only vs maternal only	0.83 (0.73-0.95)
Paternal only vs both (different)	0.57 (0.48-0.67)
Paternal only vs both the same disorder	0.42 (0.33-0.54)
Maternal only vs both (different)	0.68 (0.59-0.79)
Maternal only vs both the same disorder	0.51 (0.40-0.63)
**Bipolar**	
Paternal only vs maternal only	0.56 (0.36-0.86)
Paternal only vs both (different)	0.29 (0.19-0.47)
Paternal only vs both the same disorder	0.42 (0.06-3.08)
Maternal only vs both (different)	0.53 (0.37-0.75)
Maternal only vs both the same disorder	0.75 (0.10-5.41)

Abbreviations. ASD: Autism Spectrum Disorders; NDD: Neurodevelopmental disorders, emotional and behavioral disorders of childhood origin and intellectual disorders; ID: intellectual disability; ADHD: attention deficit hyperactivity disorder; OCD: Obsessive Compulsive Disorder; HR: Hazard Ratios; CI: confidence interval; Both (different): one parent had the specified disorder and the other parent had any other psychiatric disorder.

Note: HRs with 95% CIs were calculated using Cox regression models. Model 1: Adjusted for birth year by cubic natural splines with 5 knots.

eTable 3 (*continued*)

Analysis group	Model 1<br>HR (95% CI)
**Neurotic/behavioral disorders**	
Paternal only vs maternal only	0.85 (0.79-0.92)
Paternal only vs both (different)	0.54 (0.47-0.62)
Paternal only vs both the same category	0.47 (0.41-0.55)
Maternal only vs both (different)	0.63 (0.56-0.71)
Maternal only vs both the same category	0.56 (0.49-0.63)
**Anxiety**	
Paternal only vs maternal only	0.69 (0.59-0.80)
Paternal only vs both (different)	0.47 (0.39-0.56)
Paternal only vs both the same disorder	0.45 (0.33-0.61)
Maternal only vs both (different)	0.68 (0.59-0.79)
Maternal only vs both the same disorder	0.65 (0.49-0.86)
**OCD**	
Paternal only vs maternal only	0.51 (0.30-0.85)
Paternal only vs both (different)	0.26 (0.15-0.45)
Paternal only vs both the same disorder	0.21 (0.03-1.49)
Maternal only vs both (different)	0.51 (0.35-0.75)
Maternal only vs both the same disorder	0.41 (0.06-2.81)
**Stress-related**	
Paternal only vs maternal only	0.82 (0.70-0.95)
Paternal only vs both (different)	0.51 (0.43-0.61)
Paternal only vs both the same disorder	0.53 (0.38-0.75)
Maternal only vs both (different)	0.62 (0.54-0.73)
Maternal only vs both the same disorder	0.65 (0.47-0.90)
**Somatoform**	
Paternal only vs maternal only	0.90 (0.72-1.14)
Paternal only vs both (different)	0.42 (0.31-0.57)
Paternal only vs both the same disorder	0.85 (0.12-6.07)
Maternal only vs both (different)	0.46 (0.35-0.62)
Maternal only vs both the same disorder	0.94 (0.13-6.71)
**Eating disorders**	
Paternal only vs maternal only	0.92 (0.63-1.35)
Paternal only vs both (different)	0.50 (0.32-0.77)
Paternal only vs both the same disorder	0.49 (0.07-3.62)
Maternal only vs both (different)	0.54 (0.41-0.71)
Maternal only vs both the same disorder	0.53 (0.08-3.81)
**Sleeping disorders**	
Paternal only vs maternal only	0.79 (0.49-1.27)
Paternal only vs both (different)	0.45 (0.26-0.77)
Paternal only vs both the same disorder	-
Maternal only vs both (different)	0.57 (0.35-0.94)
Maternal only vs both the same disorder	-
**Personality disorders**	
Paternal only vs maternal only	0.65 (0.53-0.79)
Paternal only vs both (different)	0.58 (0.46-0.73)
Paternal only vs both the same disorder	0.31 (0.21-0.45)
Maternal only vs both (different)	0.89 (0.74-1.07)
Maternal only vs both the same disorder	0.48 (0.34-0.67)

Abbreviations. ASD: Autism Spectrum Disorders; NDD: Neurodevelopmental disorders, emotional and behavioral disorders of childhood origin and intellectual disorders; ID: intellectual disability; ADHD: attention deficit hyperactivity disorder; OCD: Obsessive Compulsive Disorder; HR: Hazard Ratios; CI: confidence interval; Both (different): one parent had the specified disorder and the other parent had any other psychiatric disorder.

Note: HRs with 95% CIs were calculated using Cox regression models. Model 1: Adjusted for birth year by cubic natural splines with 5 knots.

eTable 3 (*continued*)

Analysis group	Model 1 HR (95% CI)
**Substance use**	
Paternal only vs maternal only	0.88 (0.79-0.98)
Paternal only vs both (different)	0.52 (0.45-0.60)
Paternal only vs both the same category	0.68 (0.56-0.81)
Maternal only vs both (different)	0.59 (0.51-0.68)
Maternal only vs both the same category	0.77 (0.64-0.93)
**Alcohol use**	
Paternal only vs maternal only	0.93 (0.82-1.06)
Paternal only vs both (different)	0.52 (0.45-0.60)
Paternal only vs both the same disorder	0.58 (0.44-0.76)
Maternal only vs both (different)	0.56 (0.47-0.65)
Maternal only vs both the same disorder	0.62 (0.47-0.82)
**Opioid use**	
Paternal only vs maternal only	0.51 (0.30-0.87)
Paternal only vs both (different)	0.53 (0.32-0.87)
Paternal only vs both the same disorder	0.55 (0.27-1.13)
Maternal only vs both (different)	1.03 (0.63-1.69)
Maternal only vs both the same disorder	1.07 (0.52-2.20)
**Cannabis use**	
Paternal only vs maternal only	0.80 (0.50-1.29)
Paternal only vs both (different)	0.57 (0.39-0.83)
Paternal only vs both the same disorder	0.69 (0.30-1.59)
Maternal only vs both (different)	0.70 (0.44-1.13)
Maternal only vs both the same disorder	0.85 (0.35-2.08)
**Multiple/unspecific drug use**	
Paternal only vs maternal only	0.90 (0.68-1.17)
Paternal only vs both (different)	0.69 (0.53-0.89)
Paternal only vs both the same disorder	0.72 (0.48-1.07)
Maternal only vs both (different)	0.77 (0.58-1.02)
Maternal only vs both the same disorder	0.80 (0.53-1.21)
**Other**	
Paternal only vs maternal only	0.55 (0.31-1.00)
Paternal only vs both (different)	0.37 (0.20-0.71)
Paternal only vs both the same category	-
Maternal only vs both (different)	0.68 (0.40-1.16)
Maternal only vs both the same category	-

Abbreviations. ASD: Autism Spectrum Disorders; NDD: Neurodevelopmental disorders, emotional and behavioral disorders of childhood origin and intellectual disorders; ID: intellectual disability; ADHD: attention deficit hyperactivity disorder; OCD: Obsessive Compulsive Disorder; HR: Hazard Ratios; CI: confidence interval; Both (different): one parent had the specified disorder and the other parent had any other psychiatric disorder.

Note: HRs with 95% CIs were calculated using Cox regression models. Model 1: Adjusted for birth year by cubic natural splines with 5 knots.

eTable 4 Interaction between paternal and maternal psychiatric disorders

**Interaction analysis**	Maternal disorders (HR, 95% CI)		Maternal disorders within strata of paternal disorders
Paternal disorders	No	Yes	
No	1.0	2.34 (2.24, 2.43)	2.34 (2.24, 2.43)
Yes	2.02 (1.92, 2.12)	3.76 (3.48, 4.07)	1.86 (1.70, 2.04)
Paternal disorders within strata of maternal disorders	2.02 (1.92, 2.12)	1.61 (1.48, 1.76)	-
**Excess relative risk due to interaction**	**RERI (95% CI)**	***p* value**	
Any disorder	0.41 (0.09, 0.73)	0.012	
NDD	-0.41 (-2.29, 1.47)	0.67	
Schizophrenia and other non-mood psychotic disorders	2.24 (-1.53, 6.02)	0.244	
Mood disorders	1.00 (0.06, 1.93)	0.037	
Neurotic/behavioral disorders	0.65 (0.10, 1.20)	0.020	
Substance use	-0.52 (-1.01, -0.03)	0.039	
**Contrast between the hazard ratio of combined effect from both parents to that from where the parental hazards were multiplied**	**Interaction contrast (95% CI)**	***p* value**	
Any disorder	0.80 (0.72, 0.88)	<.0001	
NDD	0.49 (0.33-0.71)	0.0002	
Schizophrenia and other non-mood psychotic disorders	1.26 (0.60-2.67)	0.544	
Mood disorders	0.89 (0.71-1.12)	0.315	
Neurotic/behavioral disorders	0.81 (0.70-0.94)	0.005	
Substance use	0.61 (0.50-0.74)	<.0001	

Abbreviations. NDD: Neurodevelopmental disorders, emotional and behavioral disorders of childhood origin and intellectual disorders; HR: Hazard Ratios; CI: confidence interval; RERI: The relative excess risk due to interaction.

Note: Present measures of interaction on both additive and multiplicative scales [1,2]. Measures of interaction on additive scale: $RERI=HR_{11}-HR_{10}-HR_{01}+1$. The estimated variance of RERI was calculated using the multivariate delta method [3] with SAS codes from Li R et al [4]. Measures of interaction on multiplicative scale: $Interaction_{contrast}=HR_{11}/(HR_{10}*HR_{01})$.

References:

1. Knol MJ, VanderWeele TJ, Groenwold RH, Klungel OH, Rovers MM, Grobbee DE. Estimating measures of interaction on an additive scale for preventive exposures. *Eur J Epidemiol*. Jun 2011;26(6):433-8. doi:10.1007/s10654-011-9554-9

2. Knol MJ, VanderWeele TJ. Recommendations for presenting analyses of effect modification and interaction. *Int J Epidemiol*. Apr 2012;41(2):514-20. doi:10.1093/ije/dyr218

3. Bishop YM, Fienberg SE, Holland PW. *Discrete multivariate analysis: theory and practice*. Springer Science & Business Media; 2007.

4. Li R, Chambless L. Test for additive interaction in proportional hazards models. *Ann Epidemiol*. Mar 2007;17(3):227-36. doi:10.1016/j.annepidem.2006.10.009

eTable 5 Number of co-occurring psychiatric disorders in mothers and fathers and risk of ASD in the offspring

Number of co-occurring psychiatric disorders	**ASD (rate)**	**Number of subjects**	**Total person-years**	**Model 1 HR (95% CI)**	**Model 2 HR (95% CI)**
Paternal disorders					
None	23,694 (156.7)	1,391,053	15,119,903	Reference	Reference
One category [1]	1,638 (278.3)	70,967	588,563	1.81 (1.72-1.90)	1.45 (1.38-1.53)
Two categories	443 (323.1)	18,552	137,101	2.07 (1.88-2.27)	1.49 (1.35-1.64)
≥Three categories	189 (375.9)	8,348	50,275	2.52 (2.18-2.92)	1.66 (1.44-1.92)
Maternal disorders					
None	22,569 (152.6)	1,341,972	14,786,654	Reference	Reference
One category	2,271 (282.1)	100,364	805,157	2.05 (1.96-2.14)	1.75 (1.67-1.82)
Two categories	777 (340.6)	33,800	228,143	2.63 (2.45-2.83)	2.08 (1.93-2.24)
≥Three categories	347 (457.2)	12,784	75,889	3.57 (3.21-3.98)	2.55 (2.29-2.84)

Abbreviations. ASD: Autism Spectrum Disorders; HR: Hazard Ratios; CI: confidence interval.

Note: HRs with 95% CIs were calculated using Cox regression models. Model 1: Adjusted for birth year by cubic natural splines with 5 knots and any psychiatric history in the opposite parent; Model 2: Additionally adjusted for maternal and paternal education (<9 years primary school, 9 years primary school, 1-2 years secondary school, 3 years secondary school, 1-2 years postgraduate education, ≥ 3 years postgraduate education, PhD), income (modeled by ranks as natural cubic splines with five degrees of freedom) and age (as natural cubic splines with five degrees of freedom), all defined at delivery. Incidence rate of ASD per 100,000 person years. The co-occurring number of psychiatric diagnoses in different major categories (Neurodevelopmental disorders, emotional and behavioral disorders of childhood origin and intellectual disability; Schizophrenia and other non-mood psychotic disorders; Mood disorders; Neurotic/behavioral disorders; Psychoactive substance use; Other/unspecific psychiatric disorders).

eTable 6 Offspring of mothers with no missing data on smoking and BMI during pregnancy

Characteristics	Neither parent<br>Number of children (%)	Fathers only<br>Number of children (%)	Mothers only<br>Number of children (%)	Both parents<br>Number of children (%)
Number of individuals	1,130,404 (84.97%)	66,731 (5.02%)	111,078 (8.35%)	22,091 (1.66%)
Offspring sex (male, %)	581,662 (51.46%)	34,218 (51.28%)	57,077 (51.38%)	11,388 (51.55%)
Follow-up years*	10.8 (6.2-15.7)	7.2 (3.8-11.7)	6.6 (3.6-10.6)	5.3 (2.9- 8.6)
Birth year				
1997-2001	273,704 (24.21%)	7,664 (11.48%)	8,916 (8.03%)	1,047 (4.74%)
2002-2006	296,889 (26.26%)	11,598 (17.38%)	17,388 (15.65%)	2,209 (10.00%)
2007-2011	297,664 (26.33%)	19,856 (29.76%)	34,972 (31.48%)	6,441 (29.16%)
2012-2016	262,147 (23.19%)	27,613 (41.38%)	49,802 (44.84%)	12,394 (56.10%)
Offspring ASD	17,873 (1.58%)	1,417 (2.12%)	2,345 (2.11%)	547 (2.48%)
AD	7,907 (44.24%)	736 (51.94%)	1,246 (53.13%)	328 (59.96%)
Asperger’s syndrome	5,251 (29.38%)	336 (23.71%)	537 (22.90%)	81 (14.81%)
Other ASD	4,715 (26.38%)	345 (24.35%)	562 (23.97%)	138 (25.23%)
Comorbid with ID	2,393 (13.39%)	196 (13.83%)	309 (13.18%)	88 (16.09%)
Age at ASD*	11.0 (7.3-14.2)	9.9 (6.3-13.1)	8.6 (5.6-12.0)	7.9 (5.0-11.5)
Preterm birth (<37 week)	62,152 (5.50%)	3,984 (5.97%)	7,619 (6.86%)	1,745 (7.90%)
Gestational age				
<32 week	8,234 (0.73%)	572 (0.86%)	1,003 (0.90%)	234 (1.06%)
32-36 week	53,918 (4.77%)	3,412 (5.11%)	6,616 (5.96%)	1,511 (6.84%)
37-38	204,322 (18.08%)	12,240 (18.34%)	24,936 (22.45%)	5,078 (22.99%)
≥39 week	863,930 (76.43%)	50,507 (75.69%)	78,523 (70.69%)	15,268 (69.11%)
Maternal age at delivery*	31 (28-34)	30 (26-34)	30 (26-34)	29 (25-33)
Paternal age at delivery*	33 (29-37)	32 (28-37)	32 (28-37)	32 (27-37)
Maternal yearly income (SEK)*	144,079 (109,546-192,549)	152,571 (113,205-200,928)	146,954 (108,269-196,076)	141,105 (102,801-186,656)
Paternal yearly income (SEK)*	229,149 (167,923-302,113)	212,013 (140,821-283,605)	246,249 (179,221-315,192)	181,167 (113,138-256,621)
Maternal education				
<9 years primary school	886 (0.08%)	224 (0.34%)	610 (0.55%)	382 (1.73%)
9 years primary school	68,277 (6.04%)	9,134 (13.69%)	17,586 (15.83%)	6,733 (30.48%)
1-2 years secondary school	160,570 (14.20%)	8,960 (13.43%)	14,899 (13.41%)	3,750 (16.98%)
3 years secondary school	334,664 (29.61%)	23,765 (35.61%)	34,207 (30.80%)	6,479 (29.33%)
1-2 years postgraduate education	160,051 (14.16%)	7,395 (11.08%)	13,074 (11.77%)	1,794 (8.12%)
≥ 3 years postgraduate education	397,319 (35.15%)	16,966 (25.42%)	30,211 (27.20%)	2,919 (13.21%)
PhD	8,637 (0.76%)	287 (0.43%)	491 (0.44%)	34 (0.15%)
Paternal education				
<9 years primary school	2,337 (0.21%)	816 (1.22%)	417 (0.38%)	559 (2.53%)
9 years primary school	94,353 (8.35%)	14,538 (21.79%)	13,066 (11.76%)	6,881 (31.15%)
1-2 years secondary school	256,813 (22.72%)	15,218 (22.80%)	20,278 (18.26%)	5,077 (22.98%)
3 years secondary school	328,929 (29.10%)	19,768 (29.62%)	41,574 (37.43%)	6,315 (28.59%)
1-2 years postgraduate education	169,710 (15.01%)	6,426 (9.63%)	13,573 (12.22%)	1,522 (6.89%)
≥ 3 years postgraduate education	264,448 (23.39%)	9,574 (14.35%)	21,221 (19.10%)	1,671 (7.56%)
PhD	13,814 (1.22%)	391 (0.59%)	949 (0.85%)	66 (0.30%)
Maternal smoking (yes, %)	58,315 (5.16%)	7,888 (11.82%)	12,174 (10.96%)	5,482 (24.82%)
Maternal BMI				
<18.5	21,315 (1.89%)	1,658 (2.48%)	3,088 (2.78%)	791 (3.58%)
18.5-24.9	706,128 (62.47%)	37,571 (56.30%)	64,575 (58.13%)	11,803 (53.43%)
25-29.9	280,284 (24.80%)	17,085 (25.60%)	27,736 (24.97%)	5,640 (25.53%)
>=30	122,677 (10.85%)	10,417 (15.61%)	15,679 (14.12%)	3,857 (17.46%)

Abbreviations. ASD: autism spectrum disorders; AD: Autistic Disorder; ID: intellectual disability; Q1: 1st quartile (25th percentile), Q3: 3rd quartile (75th percentile); SEK: Swedish Krona. * Median (Q1-Q3).

eTable 7 Parental psychiatric disorders before delivery and risk of offspring ASD, adjusted for pregnancy-related risk factors

Analysis group	ASD (rate)	Number of subjects	Total person-years	Model 1 HR (95% CI)	Model 2 HR (95% CI)	Model 3 HR (95% CI)
**Any psychiatric disorder**						
Neither parent	17,873 (144.4)	1,130,404	12,377,255	Reference	Reference	Reference
Father only	1,417 (259.3)	66,731	546,536	2.06 (1.95-2.17)	1.62 (1.53-1.71)	1.57 (1.49-1.66)
Mother only	2,345 (278.7)	111,078	841,400	2.32 (2.22-2.43)	1.94 (1.86-2.03)	1.89 (1.81-1.98)
Both parents	547 (392.1)	22,091	139,489	3.72 (3.41-4.05)	2.31 (2.11-2.52)	2.21 (2.02-2.42)
**NDD**						
Father only	188 (322.3)	8,936	58,336	2.91 (2.52-3.36)	1.97 (1.70-2.28)	1.86 (1.60-2.15)
Mother only	312 (425.0)	10,624	73,411	3.68 (3.29-4.12)	2.54 (2.27-2.85)	2.41 (2.15-2.70)
Both (different)	146 (460.8)	5,872	31,682	4.97 (4.22-5.85)	2.76 (2.33-3.25)	2.59 (2.19-3.06)
Both (same)	25 (395.7)	1,520	6,317	5.71 (3.85-8.45)	2.71 (1.83-4.02)	2.58 (1.74-3.83)
**ASD**						
Father only	13 (423.5)	664	3,070	5.45 (3.16-9.39)	3.25 (1.89-5.61)	3.06 (1.77-5.28)
Mother only	21 (518.3)	890	4,052	6.67 (4.34-10.24)	4.05 (2.63-6.22)	4.07 (2.65-6.25)
Both (different)	15 (436.2)	866	3,439	6.86 (4.13-11.40)	3.38 (2.03-5.62)	3.44 (2.07-5.73)
Both (same)	4 (1454.0)	70	275	24.05 (9.02-64.14)	11.23 (4.21-29.97)	11.16 (4.18-29.80)
**ID**						
Father only	20 (386.0)	660	5,182	3.14 (2.02-4.86)	1.93 (1.24-2.99)	1.69 (1.09-2.62)
Mother only	28 (449.9)	828	6,224	3.74 (2.58-5.42)	2.32 (1.60-3.37)	2.17 (1.50-3.15)
Both (different)	27 (695.3)	668	3,883	6.88 (4.72-10.04)	3.36 (2.30-4.91)	3.03 (2.07-4.44)
Both (same)	2 (812.0)	47	246	8.83 (2.21-35.29)	4.20 (1.05-16.82)	4.05 (1.01-16.22)
**ADHD**						
Father only	57 (254.6)	4,792	22,389	3.25 (2.51-4.22)	1.92 (1.48-2.50)	1.89 (1.46-2.46)
Mother only	73 (395.9)	4,385	18,440	5.74 (4.55-7.23)	3.45 (2.74-4.36)	3.33 (2.64-4.21)
Both (different)	55 (313.8)	4,077	17,525	4.53 (3.47-5.92)	2.32 (1.77-3.03)	2.28 (1.75-2.99)
Both (same)	13 (412.1)	875	3,155	7.72 (4.49-13.27)	3.51 (2.03-6.07)	3.36 (1.94-5.80)
**Schizophrenia and other non-mood psychotic disorders**						
Father only	41 (213.5)	1,958	19,208	1.55 (1.14-2.10)	1.23 (0.90-1.67)	1.19 (0.87-1.61)
Mother only	63 (288.6)	2,207	21,828	2.10 (1.64-2.68)	1.79 (1.40-2.29)	1.70 (1.32-2.17)
Both (different)	46 (476.7)	1,339	9,649	4.12 (3.09-5.51)	2.51 (1.88-3.36)	2.37 (1.77-3.17)
Both (same)	4 (548.3)	73	730	4.04 (1.51-10.75)	2.22 (0.83-5.93)	1.93 (0.72-5.16)
**Mood disorders**						
Father only	289 (249.1)	15,639	116,025	2.10 (1.87-2.36)	1.65 (1.47-1.86)	1.61 (1.43-1.81)
Mother only	733 (281.9)	38,919	260,009	2.57 (2.39-2.77)	2.17 (2.01-2.33)	2.08 (1.93-2.24)
Both (different)	211 (388.4)	9,220	54,326	3.91 (3.41-4.48)	2.45 (2.13-2.81)	2.34 (2.04-2.68)
Both (same)	83 (454.3)	3,350	18,269	4.95 (3.99-6.15)	3.10 (2.50-3.86)	2.96 (2.38-3.68)
**Depression**						
Father only	264 (258.3)	14,153	102,217	2.22 (1.97-2.51)	1.72 (1.53-1.95)	1.68 (1.49-1.90)
Mother only	690 (286.7)	36,313	240,671	2.64 (2.44-2.85)	2.21 (2.04-2.39)	2.12 (1.96-2.29)
Both (different)	192 (368.8)	8,937	52,064	3.76 (3.26-4.34)	2.34 (2.03-2.71)	2.25 (1.94-2.60)
Both (same)	72 (463.6)	2,872	15,53	5.09 (4.03-6.42)	3.19 (2.53-4.03)	3.05 (2.41-3.85)
**Bipolar**						
Father only	24 (187.8)	1,792	12,777	1.63 (1.09-2.43)	1.34 (0.90-2.00)	1.32 (0.88-1.97)
Mother only	68 (295.7)	4	22,995	3.00 (2.37-3.81)	2.57 (2.02-3.26)	2.40 (1.89-3.05)
Both (different)	49 (487.1)	1,945	10,060	5.56 (4.20-7.37)	3.47 (2.62-4.60)	3.26 (2.46-4.32)
Both (same)	0 (0.0)	84	348	-	-	-
**Neurotic/behavioral disorders**						
Father only	732 (269.4)	34,087	271,696	2.18 (2.02-2.35)	1.74 (1.62-1.88)	1.69 (1.57-1.83)
Mother only	1,645 (291.6)	75,824	564,057	2.46 (2.34-2.59)	2.10 (1.99-2.21)	2.04 (1.94-2.15)
Both (different)	260 (405.4)	10,175	64,133	3.86 (3.41-4.36)	2.40 (2.12-2.72)	2.29 (2.02-2.60)
Both (same)	211 (435.0)	8,195	48,511	4.37 (3.81-5.00)	2.75 (2.40-3.16)	2.62 (2.28-3.01)
**Anxiety**						
Father only	201 (224.6)	13,815	89,510	2.10 (1.82-2.41)	1.65 (1.43-1.89)	1.62 (1.41-1.86)
Mother only	582 (294.1)	32,436	197,891	2.89 (2.66-3.14)	2.39 (2.19-2.60)	2.31 (2.13-2.52)
Both (different)	193 (385.5)	9,042	50,060	4.13 (3.58-4.76)	2.55 (2.21-2.95)	2.42 (2.10-2.80)
Both (same)	47 (362.5)	2,663	12,965	4.48 (3.37-5.97)	2.58 (1.94-3.44)	2.45 (1.83-3.27)

eTable 7 (*continued*)

Analysis group	ASD (rate)	Number of subjects	Total person-years	Model 1 HR (95% CI)	Model 2 HR (95% CI)	Model 3 HR (95% CI)
**OCD**						
Father only	17 (187.6)	1,408	9,059	1.75 (1.09-2.82)	1.53 (0.95-2.47)	1.48 (0.92-2.38)
Mother only	67 (314.9)	3,594	21,276	3.16 (2.48-4.02)	2.79 (2.19-3.55)	2.77 (2.18-3.52)
Both (different)	36 (553.9)	1,261	6,499	6.33 (4.56-8.78)	4.09 (2.94-5.67)	4.02 (2.90-5.58)
Both (same)	0 (0.0)	26	127	-	-	-
**Stress-related**						
Father only	214 (267.1)	10,252	80,106	2.17 (1.90-2.48)	1.66 (1.45-1.90)	1.62 (1.42-1.86)
Mother only	532 (306.5)	23,491	173,566	2.60 (2.38-2.83)	2.15 (1.97-2.34)	2.05 (1.88-2.24)
Both (different)	183 (417.1)	7,137	43,87	4.04 (3.49-4.68)	2.48 (2.14-2.88)	2.37 (2.04-2.75)
Both (same)	29 (366.7)	1,327	7,909	3.60 (2.50-5.19)	2.28 (1.58-3.28)	2.11 (1.46-3.04)
**Somatoform**						
Father only	105 (278.8)	3,963	37,667	2.06 (1.70-2.49)	1.83 (1.51-2.22)	1.76 (1.45-2.13)
Mother only	156 (275.9)	6,355	56,549	2.09 (1.78-2.44)	1.80 (1.54-2.11)	1.74 (1.48-2.04)
Both (different)	54 (499.6)	1,552	10,808	4.39 (3.36-5.74)	2.94 (2.25-3.85)	2.73 (2.09-3.57)
Both (same)	1 (315.0)	40	317	2.59 (0.36-18.38)	2.09 (0.29-14.83)	2.13 (0.30-15.14)
**Eating disorders**						
Father only	26 (227.4)	1,649	11,436	2.01 (1.37-2.96)	1.71 (1.16-2.52)	1.62 (1.11-2.39)
Mother only	261 (260.5)	13,703	100,176	2.20 (1.95-2.49)	1.97 (1.75-2.23)	2.04 (1.81-2.31)
Both (different)	52 (381.2)	2,347	13,641	3.88 (2.96-5.10)	2.52 (1.92-3.31)	2.55 (1.94-3.35)
Both (same)	1 (449.2)	34	223	4.28 (0.60-30.35)	3.51 (0.49-24.89)	3.86 (0.54-27.40)
**Sleeping disorders**						
Father only	25 (246.0)	1,462	10,163	2.15 (1.45-3.18)	1.78 (1.20-2.63)	1.75 (1.18-2.60)
Mother only	32 (238.4)	2,208	13,423	2.31 (1.63-3.27)	1.96 (1.38-2.77)	1.87 (1.32-2.65)
Both (different)	22 (378.9)	1,180	5,807	4.57 (3.01-6.94)	2.54 (1.67-3.87)	2.49 (1.64-3.79)
Both (same)	0 (0.0)	41	164	-	-	-
**Personality disorders**						
Father only	115 (353.3)	3,525	32,546	2.65 (2.21-3.19)	1.81 (1.50-2.17)	1.72 (1.43-2.07)
Mother only	299 (485.0)	8,042	61,654	4.01 (3.57-4.49)	3.03 (2.70-3.40)	2.84 (2.53-3.19)
Both (different)	136 (511.5)	4,071	26,588	4.71 (3.98-5.58)	2.78 (2.34-3.30)	2.63 (2.22-3.13)
Both (same)	25 (859.2)	427	2,910	7.71 (5.21-11.41)	4.23 (2.86-6.27)	3.91 (2.64-5.80)
**Substance use**						
Father only	581 (258.7)	26,576	224,547	2.01 (1.85-2.18)	1.48 (1.36-1.61)	1.44 (1.32-1.56)
Mother only	492 (272.7)	23,815	180,394	2.26 (2.07-2.48)	1.69 (1.54-1.85)	1.64 (1.50-1.80)
Both (different)	219 (404.6)	8,664	54,128	3.85 (3.37-4.40)	2.36 (2.06-2.71)	2.25 (1.96-2.58)
Both (same)	118 (347.7)	4,827	33,934	3.03 (2.53-3.64)	1.66 (1.38-2.00)	1.61 (1.34-1.93)
**Alcohol use**						
Father only	430 (250.8)	20,239	171,445	1.95 (1.77-2.14)	1.48 (1.34-1.63)	1.43 (1.29-1.57)
Mother only	343 (251.1)	18,401	136,601	2.11 (1.90-2.35)	1.61 (1.45-1.80)	1.58 (1.42-1.76)
Both (different)	212 (408.8)	8,023	51,858	3.80 (3.32-4.35)	2.30 (2.00-2.64)	2.19 (1.91-2.52)
Both (same)	50 (380.7)	1,980	13,132	3.46 (2.62-4.57)	1.95 (1.48-2.58)	1.87 (1.42-2.48)
**Opioid use**						
Father only	25 (200.7)	1,441	12,458	1.53 (1.04-2.27)	1.02 (0.69-1.50)	1.01 (0.68-1.50)
Mother only	22 (319.4)	858	6,889	2.56 (1.69-3.89)	1.73 (1.14-2.63)	1.64 (1.08-2.49)
Both (different)	31 (317.5)	1,461	9,764	2.88 (2.02-4.10)	1.56 (1.09-2.22)	1.53 (1.08-2.18)
Both (same)	7 (296.5)	312	2,361	2.46 (1.17-5.16)	1.29 (0.61-2.70)	1.28 (0.61-2.68)
**Cannabis use**						
Father only	47 (237.2)	2,251	19,81	1.80 (1.35-2.40)	1.17 (0.88-1.57)	1.16 (0.87-1.54)
Mother only	18 (240.8)	932	7,475	1.94 (1.22-3.08)	1.21 (0.76-1.92)	1.18 (0.74-1.87)
Both (different)	40 (310.4)	2,014	12,886	2.86 (2.10-3.90)	1.54 (1.13-2.11)	1.53 (1.12-2.09)
Both (same)	6 (415.2)	202	1,445	3.59 (1.61-7.98)	1.79 (0.81-3.99)	1.70 (0.76-3.80)
**Multiple/unspecific drug use**						
Father only	103 (271.3)	4,634	37,962	2.15 (1.77-2.61)	1.37 (1.13-1.67)	1.35 (1.11-1.64)
Mother only	75 (289.1)	3,345	25,939	2.36 (1.88-2.96)	1.59 (1.26-1.99)	1.52 (1.21-1.91)
Both (different)	92 (355.6)	3,933	25,873	3.25 (2.65-3.99)	1.78 (1.45-2.19)	1.72 (1.40-2.11)
Both (same)	25 (345.4)	1,101	7,237	3.19 (2.15-4.72)	1.57 (1.06-2.32)	1.53 (1.03-2.27)
**Other**						
Father only	15 (237.0)	750	6,328	1.82 (1.10-3.02)	1.39 (0.84-2.31)	1.36 (0.82-2.25)
Mother only	31 (347.7)	1,311	8,915	3.09 (2.17-4.39)	2.35 (1.65-3.34)	2.23 (1.57-3.17)
Both (different)	16 (373.9)	738	4,279	3.74 (2.29-6.10)	2.21 (1.36-3.61)	1.96 (1.20-3.21)
Both (same)	0 (0.0)	31	127	-	-	-

Abbreviations. ASD: Autism Spectrum Disorders; NDD: Neurodevelopmental disorders, emotional and behavioral disorders of childhood origin and intellectual disability; ID: intellectual disability; ADHD: attention deficit hyperactivity

disorder; OCD: Obsessive Compulsive Disorder; BMI: body mass index; HR: Hazard Ratios; CI: confidence interval; Both (different): one parent had the specified disorder and the other parent had any other psychiatric disorder; Both (same): both parents had the same specific disorder.

Note: HRs with 95% CIs were calculated using Cox regression models. Model 1: Adjusted for birth year by cubic natural splines with 5 knots; Model 2: Additionally adjusted for maternal and paternal education (<9 years primary school, 9 years primary school, 1-2 years secondary school, 3 years secondary school, 1-2 years postgraduate education, ≥ 3 years postgraduate education, PhD), income (modeled by ranks as natural cubic splines with five degrees of freedom) and age (as natural cubic splines with five degrees of freedom), all defined at delivery. Model 3: Additionally adjusted for pregnancy-related risk factors, including preterm birth (yes vs no), maternal smoking (yes vs no) and maternal BMI (<18.5, 18.5-24.9, 25-29.9, ≥30 kg/m2) during pregnancy. Incidence rate of ASD per 100,000 person years. The reference group of all subgroups was offspring of parents without any psychiatric disorder.

eTable 8 Parental psychiatric disorders before delivery and risk of offspring ASD among parents with only one psychiatric category

Analysis group	ASD (rate)	Number of subjects	Total person-years	Model 1 HR (95% CI)	Model 2 HR (95% CI)
**Any psychiatric disorder**					
Neither parent	20,947 (147.8)	1,268,419	14,168,262	Reference	Reference
Father only	1,239 (254.5)	55,998	486,824	1.93 (1.82-2.04)	1.55 (1.46-1.64)
Mother only	1,931 (270.9)	87,087	712,764	2.13 (2.03-2.23)	1.81 (1.72-1.89)
Both parents	220 (340.7)	8,930	64,575	2.90 (2.54-3.32)	1.91 (1.67-2.19)
**NDD**					
Father only	111 (333.2)	4,626	33,315	2.76 (2.29-3.33)	1.91 (1.59-2.31)
Mother only	140 (384.3)	4,327	36,431	2.94 (2.49-3.47)	2.06 (1.75-2.44)
Both (different)	30 (389.7)	1,252	7,698	3.69 (2.58-5.27)	2.20 (1.54-3.15)
Both (same)	-	143	686	-	-
**Schizophrenia and other non-mood psychotic disorders**					
Father only	21 (249.5)	693	8,418	1.65 (1.08-2.54)	1.49 (0.97-2.28)
Mother only	25 (276.7)	690	9,035	1.78 (1.20-2.64)	1.66 (1.12-2.45)
Both (different)	4 (312.6)	112	1,279	2.18 (0.82-5.80)	1.60 (0.60-4.27)
Both (same)	-	-	-	-	-
**Mood disorders**					
Father only	137 (239.6)	6,696	57,167	1.84 (1.56-2.18)	1.56 (1.32-1.85)
Mother only	289 (245.6)	15,454	117,687	2.03 (1.81-2.29)	1.79 (1.59-2.01)
Both (different)	40 (283.0)	2,063	14,136	2.54 (1.86-3.46)	1.81 (1.32-2.46)
Both (same)	5 (260.6)	283	1,919	2.42 (1.01-5.82)	1.81 (0.75-4.36)
**Neurotic/behavioral disorders**					
Father only	506 (250.3)	23,563	202,196	1.92 (1.76-2.09)	1.64 (1.50-1.79)
Mother only	1,175 (276.2)	51,729	425,492	2.17 (2.04-2.30)	1.91 (1.80-2.03)
Both (different)	108 (382.5)	4,026	28,234	3.36 (2.78-4.06)	2.27 (1.88-2.75)
Both (same)	47 (354.7)	1,911	13,252	3.13 (2.35-4.16)	2.30 (1.73-3.06)
**Substance use**					
Father only	461 (251.6)	20,168	183,239	1.86 (1.69-2.04)	1.41 (1.28-1.54)
Mother only	300 (246.3)	14,599	121,790	1.91 (1.70-2.14)	1.44 (1.29-1.62)
Both (different)	84 (366.9)	3,204	22,897	3.15 (2.54-3.90)	2.06 (1.66-2.55)
Both (same)	34 (297.6)	1,236	11,425	2.19 (1.56-3.06)	1.24 (0.89-1.74)
**Other**					
Father only	3 (131.8)	239	2,276	0.95 (0.31-2.94)	0.82 (0.26-2.53)
Mother only	2 (90.8)	281	2,203	0.73 (0.18-2.93)	0.58 (0.14-2.31)
Both (different)	2 (584.2)	57	342	5.65 (1.41-22.53)	3.70 (0.93-14.76)
Both (same)	-	-	-	-	-

Abbreviations. ASD: Autism Spectrum Disorders; NDD: Neurodevelopmental disorders, emotional and behavioral disorders of childhood origin and intellectual disability; HR: Hazard Ratios; CI: confidence interval; Both (different): one parent had the specified disorder and the other parent had any other psychiatric disorder; Both (same): both parents had the same specific disorder.

Note: HRs with 95% CIs were calculated using Cox regression models. Model 1: Adjusted for birth year by cubic natural splines with 5 knots; Model 2: Additionally adjusted for maternal and paternal education (<9 years primary school, 9 years primary school, 1-2 years secondary school, 3 years secondary school, 1-2 years postgraduate education, ≥ 3 years postgraduate education, PhD), income (modeled by ranks as natural cubic splines with five degrees of freedom) and age (as natural cubic splines with five degrees of freedom), all defined at delivery. Incidence rate of ASD per 100,000 person years. The analyzed population was restricted to mothers and fathers only with one major category and undiagnosed parents. The reference group of all subgroups was offspring of parents without any psychiatric disorder.

eTable 9 Parental psychiatric disorders first diagnosed prior to one year before conception and risk of ASD

Parental psychiatric disorders	ASD (rate)	Number of subjects	Person-years	Model 1<br>HR (95% CI)	Model 2<br>HR (95% CI)
Neither parent	21,555 (149.8)	1,293,935	14,387,322	Reference	Reference
Fathers only	1,517 (270.7)	67,483	560,319	2.06 (1.95-2.17)	1.60 (1.52-1.69)
Mothers only	2,415 (290.9)	108,788	830,212	2.33 (2.24-2.43)	1.94 (1.85-2.02)
Both parents	477 (404.3)	18,714	117,99	3.69 (3.37-4.04)	2.29 (2.08-2.51)

Abbreviations. ASD: Autism Spectrum Disorders; HR: Hazard Ratios; CI: confidence interval.

Note: HRs with 95% CIs were calculated using Cox regression models. Model 1: Adjusted for birth year by cubic natural splines with 5 knots; Model 2: Additionally adjusted for maternal and paternal education (<9 years primary school, 9 years primary school, 1-2 years secondary school, 3 years secondary school, 1-2 years postgraduate education, ≥ 3 years postgraduate education, PhD), income (modeled by ranks as natural cubic splines with five degrees of freedom) and age (as natural cubic splines with five degrees of freedom), all defined at delivery. Incidence rate of ASD per 100,000 person years.

eTable 10 Parental psychiatric disorders occurred at least twice with an interval of more than 30 days

Parental psychiatric disorders	ASD (rate)	Number of subjects	Person-years	Model 1 HR (95% CI)	Model 2 HR (95% CI)
Neither parent	23,502 (155.1)	1,382,692	15,155,934	Reference	Reference
Fathers only	756 (299.1)	33,581	252,750	2.31 (2.15-2.49)	1.62 (1.50-1.74)
Mothers only	1,451 (337.5)	63,078	429,905	2.79 (2.65-2.95)	2.20 (2.08-2.32)
Both parents	255 (445.4)	9,569	57,253	4.07 (3.59-4.60)	2.26 (1.99-2.56)

Abbreviations. HR: Hazard Ratios; CI: confidence interval.

Note: HRs with 95% CIs were calculated using Cox regression models. Model 1: Adjusted for birth year by cubic natural splines with 5 knots; Model 2: Additionally adjusted for maternal and paternal education (<9 years primary school, 9 years primary school, 1-2 years secondary school, 3 years secondary school, 1-2 years postgraduate education, ≥ 3 years postgraduate education, PhD), income (modeled by ranks as natural cubic splines with five degrees of freedom) and age (as natural cubic splines with five degrees of freedom), all defined at delivery. Incidence rate of ASD per 100,000 person years.

eTable 11 Parental psychiatric disorders and risk of autistic disorder in offspring

Parental psychiatric disorders	ASD (rate)	Number of subjects	Person-years	Model 1 HR (95% CI)	Model 2 HR (95% CI)
Neither parent	10,390 (73.1)	1,268,419	14,215,648	Reference	Reference
Fathers only	928 (149.3)	73,553	621,435	2.10 (1.96-2.25)	1.63 (1.52-1.74)
Mothers only	1,592 (166.5)	122,634	956,435	2.35 (2.22-2.48)	1.94 (1.84-2.05)
Both parents	425 (268.2)	24,314	158,447	4.02 (3.64-4.43)	2.39 (2.16-2.65)

Abbreviations. HR: Hazard Ratios; CI: confidence interval.

Note: HRs with 95% CIs were calculated using Cox regression models. Model 1: Adjusted for birth year by cubic natural splines with 5 knots; Model 2: Additionally adjusted for maternal and paternal education (<9 years primary school, 9 years primary school, 1-2 years secondary school, 3 years secondary school, 1-2 years postgraduate education, ≥ 3 years postgraduate education, PhD), income (modeled by ranks as natural cubic splines with five degrees of freedom) and age (as natural cubic splines with five degrees of freedom), all defined at delivery. Incidence rate of ASD per 100,000 person years.

eTable 12 Parental psychiatric disorders and risk of ASD among first born offspring

Analysis group	ASD (rate)	Number of subjects	Total person-years	Model 1 HR (95% CI)	Model 2 HR (95% CI)
**Any psychiatric disorder**					
Neither parent	14,685 (163.9)	719,309	8,960,796	Reference	Reference
Father only	1,106 (292.9)	40,69	377,567	1.96 (1.84-2.08)	1.56 (1.46-1.66)
Mother only	1,806 (330.0)	63,767	547,319	2.27 (2.16-2.39)	1.91 (1.82-2.01)
Both parents	423 (463.3)	12,872	91,31	3.52 (3.19-3.88)	2.24 (2.02-2.47)
**NDD**					
Father only	151 (364.1)	5,755	41,468	2.73 (2.32-3.20)	1.91 (1.63-2.25)
Mother only	234 (458.7)	6,639	51,013	3.35 (2.94-3.81)	2.37 (2.08-2.70)
Both (different)	112 (545.8)	3,527	20,521	4.75 (3.94-5.72)	2.73 (2.26-3.30)
Both (same)	19 (435.1)	998	4,367	4.92 (3.14-7.73)	2.50 (1.59-3.93)
**Schizophrenia and other non-mood psychotic disorders**					
Father only	36 (251.3)	1,273	14,324	1.58 (1.14-2.19)	1.27 (0.92-1.76)
Mother only	56 (335.7)	1,458	16,679	2.12 (1.63-2.75)	1.78 (1.37-2.31)
Both (different)	44 (589.7)	872	7,461	4.14 (3.08-5.56)	2.58 (1.92-3.48)
Both (same)	5 (695.9)	67	718	4.57 (1.90-10.98)	2.70 (1.12-6.50)
**Mood disorders**					
Father only	219 (288.2)	9,17	75,99	2.02 (1.77-2.31)	1.61 (1.41-1.85)
Mother only	544 (336.9)	21,582	161,491	2.48 (2.27-2.70)	2.09 (1.92-2.28)
Both (different)	164 (478.3)	5,23	34,287	3.83 (3.28-4.47)	2.46 (2.10-2.88)
Both (same)	60 (544.2)	1,876	11,025	4.72 (3.66-6.09)	3.06 (2.37-3.95)
**Neurotic/behavioral disorders**					
Father only	550 (304.1)	20,047	180,89	2.06 (1.89-2.24)	1.67 (1.53-1.82)
Mother only	1,266 (352.3)	42,735	359,324	2.45 (2.31-2.60)	2.11 (1.99-2.23)
Both (different)	205 (484.8)	5,989	42,29	3.71 (3.23-4.26)	2.36 (2.05-2.72)
Both (same)	155 (527.4)	4,488	29,39	4.22 (3.60-4.95)	2.74 (2.33-3.22)
**Substance use**					
Father only	457 (283.9)	16,668	160,989	1.86 (1.70-2.04)	1.39 (1.27-1.53)
Mother only	393 (310.8)	14,686	126,444	2.12 (1.92-2.34)	1.61 (1.45-1.78)
Both (different)	167 (474.9)	5,013	35,165	3.63 (3.12-4.23)	2.30 (1.97-2.68)
Both (same)	95 (380.3)	3,108	24,979	2.69 (2.20-3.30)	1.51 (1.23-1.85)
**Other**					
Father only	10 (207.7)	510	4,814	1.37 (0.74-2.55)	1.07 (0.57-1.98)
Mother only	24 (414.3)	757	5,792	3.00 (2.01-4.47)	2.29 (1.54-3.43)
Both (different)	16 (537.8)	445	2,975	4.26 (2.61-6.96)	2.52 (1.55-4.12)
Both (same)	0 (0.0)	18	98	-	-

Abbreviations. ASD: Autism Spectrum Disorders; NDD: neurodevelopmental disorders, emotional and behavioral disorders of childhood origin and intellectual disability; HR: Hazard Ratios; CI: confidence interval; Both (different): one parent had the specified disorder and the other parent had any other psychiatric disorder; Both (same): both parents had the same specific disorder.

Note: HRs with 95% CIs were calculated using Cox regression models. Model 1: Adjusted for birth year by cubic natural splines with 5 knots; Model 2: Additionally adjusted for maternal and paternal education (<9 years primary school, 9 years primary school, 1-2 years secondary school, 3 years secondary school, 1-2 years postgraduate education, ≥ 3 years postgraduate education, PhD), income (modeled by ranks as natural cubic splines with five degrees of freedom) and age (as natural cubic splines with five degrees of freedom), all defined at delivery. Incidence rate of ASD per 100,000 person years. The reference group of all subgroups was offspring of parents without any psychiatric disorder.

eTable 13 Parental psychiatric disorders and risk of ASD among offspring born 2007-2016

Analysis group	ASD (rate)	Number of subjects	Total person-years	Model 1 HR (95% CI)	Model 2 HR (95% CI)
**Any psychiatric disorder**					
Neither parent	3,644 (97.4)	601,697	3,741,569	Reference	Reference
Father only	452 (162.7)	50,881	277,793	1.84 (1.67-2.03)	1.56 (1.41-1.72)
Mother only	1,046 (210.5)	91,564	496,826	2.40 (2.24-2.57)	2.11 (1.97-2.27)
Both parents	314 (310.5)	20,38	101,127	3.84 (3.42-4.31)	2.67 (2.36-3.02)
**NDD**					
Father only	94 (251.6)	7,887	37,356	3.24 (2.64-3.98)	2.47 (2.00-3.05)
Mother only	143 (332.0)	9,067	43,072	4.24 (3.59-5.01)	3.22 (2.71-3.82)
Both (different)	90 (345.5)	5,781	26,051	4.71 (3.82-5.81)	3.10 (2.50-3.86)
Both (same)	23 (378.2)	1,582	6,081	6.29 (4.17-9.49)	3.78 (2.49-5.74)
**Schizophrenia and other non-mood psychotic disorders**					
Father only	13 (181.5)	1,243	7,162	1.97 (1.14-3.39)	1.52 (0.88-2.63)
Mother only	26 (321.1)	1,399	8,097	3.47 (2.36-5.11)	2.78 (1.89-4.10)
Both (different)	22 (374.0)	1,158	5,883	4.54 (2.98-6.90)	2.82 (1.85-4.30)
Both (same)	1 (303.0)	51	330	2.89 (0.41-20.55)	1.49 (0.21-10.61)
**Mood disorders**					
Father only	125 (180.9)	13,017	69,081	2.10 (1.76-2.52)	1.71 (1.43-2.05)
Mother only	416 (225.2)	35,18	184,732	2.65 (2.39-2.93)	2.29 (2.06-2.54)
Both (different)	147 (344.1)	8,822	42,716	4.36 (3.69-5.14)	3.04 (2.56-3.61)
Both (same)	62 (386.4)	3,356	16,047	4.96 (3.86-6.38)	3.42 (2.65-4.41)
**Neurotic/behavioral disorders**					
Father only	235 (160.9)	26,786	146,096	1.83 (1.60-2.09)	1.54 (1.35-1.77)
Mother only	768 (225.3)	63,372	340,804	2.59 (2.40-2.80)	2.29 (2.11-2.48)
Both (different)	140 (301.6)	9,365	46,42	3.73 (3.15-4.41)	2.60 (2.18-3.09)
Both (same)	142 (372.4)	7,83	38,13	4.68 (3.95-5.53)	3.23 (2.71-3.84)
**Substance use**					
Father only	160 (149.6)	19,548	106,981	1.69 (1.44-1.98)	1.37 (1.17-1.61)
Mother only	203 (192.1)	19,57	105,701	2.20 (1.91-2.54)	1.81 (1.57-2.09)
Both (different)	118 (298.3)	8,001	39,551	3.69 (3.07-4.43)	2.49 (2.06-3.01)
Both (same)	55 (254.2)	4,225	21,637	3.06 (2.35-4.00)	1.87 (1.43-2.46)
**Other**					
Father only	3 (105.8)	560	2,837	1.27 (0.41-3.94)	0.99 (0.32-3.06)
Mother only	15 (256.6)	1,143	5,845	3.05 (1.84-5.07)	2.28 (1.37-3.80)
Both (different)	9 (294.6)	690	3,055	4.11 (2.13-7.90)	2.63 (1.36-5.07)
Both (same)	0 (0.0)	30	112	-	-

Abbreviations. ASD: Autism Spectrum Disorders; NDD: neurodevelopmental disorders, emotional and behavioral disorders of childhood origin and intellectual disability; ID: intellectual disability; ADHD: attention deficit hyperactivity disorder; OCD: Obsessive Compulsive Disorder; HR: Hazard Ratios; CI: confidence interval; ; Both (different): one parent had the specified disorder and the other parent had any other psychiatric disorder; Both (same): both parents had the same specific disorder.

Note: HRs with 95% CIs were calculated using Cox regression models. Model 1: Adjusted for birth year by cubic natural splines with 5 knots; Model 2: Additionally adjusted for maternal and paternal education (<9 years primary school, 9 years primary school, 1-2 years secondary school, 3 years secondary school, 1-2 years postgraduate education, ≥ 3 years postgraduate education, PhD), income (modeled by ranks as natural cubic splines with five degrees of freedom) and age (as natural cubic splines with five degrees of freedom), all defined at delivery. Incidence rate of ASD per 100,000 person years. The reference group of all subgroups was offspring of parents without any psychiatric disorder.

eTable 14 Parental psychiatric disorders and risk of ASD among offspring born 1997-2012

Parental psychiatric disorders	ASD (rate)	Number of subjects	Total person-years	Model 1 HR (95% CI)	Model 2 HR (95% CI)
Neither parent	20,721 (153.6)	1,047,837	13,489,323	Reference	Reference
Fathers only	1,576 (288.3)	49,355	546,645	2.02 (1.92-2.12)	1.58 (1.50-1.67)
Mothers only	2,663 (324.0)	78,863	821,798	2.34 (2.25-2.44)	1.96 (1.88-2.04)
Both parents	611 (489.5)	13,090	124,812	3.76 (3.47-4.08)	2.33 (2.15-2.54)

Abbreviations. ASD: Autism Spectrum Disorders; HR: Hazard Ratios; CI: confidence interval.
Note: Population: children born between 1997 and 2012. HRs with 95% CIs were calculated using Cox regression models. Model 1: Adjusted for birth year by cubic natural splines with 5 knots; Model 2: Additionally adjusted for maternal and paternal education (<9 years primary school, 9 years primary school, 1-2 years secondary school, 3 years secondary school, 1-2 years postgraduate education, ≥ 3 years postgraduate education, PhD), income (modeled by ranks as natural cubic splines with five degrees of freedom) and age (as natural cubic splines with five degrees of freedom), all defined at delivery. Incidence rate of ASD per 100,000 person years.

eTable 15 Parental psychiatric disorders and risk of ASD among singletons

Parental psychiatric disorders	ASD (rate)	Number of subjects	Total person-years	Model 1 HR (95% CI)	Model 2 HR (95% CI)
Neither parent	20,343 (148.1)	1,230,394	13,736,268	Reference	Reference
Fathers only	1,569 (261.4)	71,417	600,180	2.01 (1.91-2.12)	1.58 (1.50-1.67)
Mothers only	2,658 (287.8)	118,945	923,441	2.33 (2.23-2.43)	1.94 (1.86-2.03)
Both parents	628 (410.2)	23,620	153,107	3.75 (3.46-4.07)	2.33 (2.14-2.53)

Abbreviations. ASD: Autism Spectrum Disorders; HR: Hazard Ratios; CI: confidence interval.

Note: HRs with 95% CIs were calculated using Cox regression models. Model 1: Adjusted for birth year by cubic natural splines with 5 knots; Model 2: Additionally adjusted for maternal and paternal education (<9 years primary school, 9 years primary school, 1-2 years secondary school, 3 years secondary school, 1-2 years postgraduate education, ≥ 3 years postgraduate education, PhD), income (modeled by ranks as natural cubic splines with five degrees of freedom) and age (as natural cubic splines with five degrees of freedom), all defined at delivery. Incidence rate of ASD per 100,000 person years.

eTable 16 Parental psychiatric disorders and risk of ASD among offspring without malformation

Parental psychiatric disorders	ASD (rate)	Number of subjects	Total person-years	Model 1 HR (95% CI)	Model 2 HR (95% CI)
Neither parent	19,778 (144.7)	1,223,376	13,672,707	Reference	Reference
Fathers only	1,538 (258.1)	70,838	595,794	2.04 (1.93-2.15)	1.60 (1.52-1.69)
Mothers only	2,590 (282.3)	118,203	917,465	2.34 (2.25-2.44)	1.96 (1.88-2.04)
Both parents	616 (405.9)	23,414	151,768	3.82 (3.52-4.14)	2.37 (2.18-2.58)

Abbreviations. ASD: Autism Spectrum Disorders; HR: Hazard Ratios; CI: confidence interval.

Note: HRs with 95% CIs were calculated using Cox regression models. Model 1: Adjusted for birth year by cubic natural splines with 5 knots; Model 2: Additionally adjusted for maternal and paternal education (<9 years primary school, 9 years primary school, 1-2 years secondary school, 3 years secondary school, 1-2 years postgraduate education, ≥ 3 years postgraduate education, PhD), income (modeled by ranks as natural cubic splines with five degrees of freedom) and age (as natural cubic splines with five degrees of freedom), all defined at delivery. Incidence rate of ASD per 100,000 person years.

eTable 17 Any non-ASD psychiatric disorders in parents and risk of ASD in offspring

Parental psychiatric disorders	ASD (rate)	Number of subjects	Total person-years	Model 1 HR (95% CI)	Model 2 HR (95% CI)
Neither parent	20,947 (147.8)	1,268,419	14,168,262	Reference	Reference
Fathers only	1,605 (261.0)	72,832	615,012	2.01 (1.91-2.11)	1.58 (1.50-1.66)
Mothers only	2,725 (287.7)	121,642	947,138	2.33 (2.23-2.42)	1.95 (1.87-2.03)
Both parents	626 (408.0)	23,290	153,430	3.70 (3.41-4.01)	2.31 (2.13-2.51)

Abbreviations. ASD: Autism Spectrum Disorders; HR: Hazard Ratios; CI: confidence interval.

Note: HRs with 95% CIs were calculated using Cox regression models. Model 1: Adjusted for birth year by cubic natural splines with 5 knots; Model 2: Additionally adjusted for maternal and paternal education (<9 years primary school, 9 years primary school, 1-2 years secondary school, 3 years secondary school, 1-2 years postgraduate education, ≥ 3 years postgraduate education, PhD), income (modeled by ranks as natural cubic splines with five degrees of freedom) and age (as natural cubic splines with five degrees of freedom), all defined at delivery. Incidence rate of ASD per 100,000 person years.

eTable 18 Parental psychiatric disorders and risk of offspring ASD by gestational age categories

Gestational age	Parental psychiatric disorders	ASD (rate)	Number of subjects	Total person-years	Model 1 HR (95% CI)	Model 2 HR (95% CI)
Term birth ≥37 weeks	Neither parent	19,213 (143.9)	1,195,329	13,349,246	Reference	Reference
	Fathers only	1,472 (254.1)	68,956	579,252	2.01 (1.91-2.12)	1.59 (1.50-1.67)
	Mothers only	2,464 (279.4)	113,715	881,816	2.32 (2.23-2.42)	1.95 (1.87-2.03)
	Both parents	578 (401.1)	22,286	144,102	3.77 (3.47-4.10)	2.36 (2.16-2.57)
Preterm birth <37 weeks	Neither parent	1,734 (211.7)	73,090	819,016	Reference	Reference
	Fathers only	150 (383.2)	4,597	39,140	2.04 (1.72-2.41)	1.59 (1.34-1.88)
	Mothers only	283 (405.3)	8,919	69,825	2.27 (2.00-2.58)	1.88 (1.66-2.13)
	Both parents	70 (520.6)	2,028	13,446	3.26 (2.57-4.14)	2.07 (1.63-2.63)
Very preterm <32 week	Neither parent	421 (393.8)	10,510	106,916	Reference	Reference
	Fathers only	33 (589.3)	725	5,600	1.66 (1.17-2.37)	1.31 (0.92-1.86)
	Mothers only	58 (587.4)	1,366	9,873	1.76 (1.33-2.31)	1.43 (1.09-1.88)
	Both parents	14 (790.3)	292	1,771	2.67 (1.57-4.54)	1.78 (1.04-3.03)
Moderate-to-late preterm 32-36 week	Neither parent	1,313 (184.4)	62,580	712,100	Reference	Reference
	Fathers only	117 (348.8)	3,872	33,540	2.13 (1.77-2.58)	1.66 (1.38-2.01)
	Mothers only	225 (375.3)	7,553	59,951	2.42 (2.10-2.79)	2.01 (1.74-2.32)
	Both parents	56 (479.7)	1,736	11,674	3.44 (2.63-4.50)	2.17 (1.66-2.84)
Early term 37-38 week	Neither parent	4,098 (157.3)	230,837	2,605,083	Reference	Reference
	Fathers only	315 (267.3)	13,613	117,846	1.91 (1.70-2.14)	1.50 (1.34-1.68)
	Mothers only	690 (319.1)	27,662	216,253	2.43 (2.24-2.63)	2.04 (1.88-2.21)
	Both parents	171 (466.4)	5,627	36,667	4.02 (3.45-4.69)	2.51 (2.15-2.93)
Full term ≥39 week	Neither parent	15,115 (140.7)	964,492	10,744,163	Reference	Reference
	Fathers only	1,157 (250.8)	55,343	461,406	2.04 (1.92-2.16)	1.61 (1.52-1.71)
	Mothers only	1,774 (266.5)	86,053	665,563	2.27 (2.16-2.38)	1.90 (1.81-2.00)
	Both parents	407 (378.8)	16,659	107,435	3.65 (3.30-4.03)	2.29 (2.07-2.53)

Abbreviations. ASD: Autism Spectrum Disorders; HR: Hazard Ratios; CI: confidence interval.

Note: HRs with 95% CIs were calculated using Cox regression models including interaction term between parental psychiatric history before delivery and gestational age categories. Strata-specific effect is shown. Model 1: Adjusted for birth year by cubic natural splines with 5 knots; Model 2: Additionally adjusted for maternal and paternal education (<9 years primary school, 9 years primary school, 1-2 years secondary school, 3 years secondary school, 1-2 years postgraduate education, ≥ 3 years postgraduate education, PhD), income (modeled by ranks as natural cubic splines with five degrees of freedom) and age (as natural cubic splines with five degrees of freedom), all defined at delivery. Incidence rate of ASD per 100,000 person years. Test for interaction term parental psychiatric disorders*preterm p=0.70, parental psychiatric disorders *gestational age categories p=0.27.

eTable 19 Mediation analysis of the association between parental psychiatric disorders and offspring ASD bypreterm and early term birth

Mediation by GA	Psychiatric disorders in fathers only	Psychiatric disorders in mothers only	Psychiatric disorders in both parents
Number of subjects	73,553	122,634	24,314
ASD (%)	20,947 (1.65%)	1,622 (2.21%)	2,747 (2.24%)
**Preterm (< 37 weeks)**			
Preterm (%)	4,597 (6.25%)	8,919 (7.27%)	2,028 (8.34%)
**Total effect**			
Model 1	1.99 (1.88-2.09)	2.27 (2.18-2.37)	3.56 (3.27-3.85)
Model 2	1.65 (1.57-1.74)	1.94 (1.86-2.02)	2.43 (2.23-2.64)
**NDE**			
Model 1	1.98 (1.88-2.08)	2.26 (2.16-2.35)	3.52 (3.23-3.80)
Model 2	1.65 (1.56-1.74)	1.93 (1.84-2.01)	2.41 (2.21-2.61)
**NIE**			
Model 1	1.00 (1.00-1.00)	1.01 (1.01-1.01)	1.01 (1.01-1.01)
Model 2	1.00 (1.00-1.00)	1.01 (1.00-1.01)	1.01 (1.01-1.01)
**Percentage mediated (%)**			
Model 1	0.56 (0.38-0.75)	1.31 (1.08-1.55)	1.71 (1.37-2.06)
Model 2	0.40 (0.20-0.60)	1.22 (0.98-1.45)	1.54 (1.18-1.89)
**Early term (< 39 weeks)**			
Preterm (%)	18,210 (24.76%)	36,581 (29.83%)	7,655 (31.48%)
**Total effect**			
Model 1	1.99 (1.88-2.09)	2.27 (2.18-2.37)	3.57 (3.27-3.86)
Model 2	1.65 (1.56-1.74)	1.94 (1.86-2.02)	2.44 (2.23-2.64)
**NDE**			
Model 1	1.99 (1.88-2.09)	2.27 (2.18-2.37)	3.51 (3.22-3.80)
Model 2	1.65 (1.56-1.74)	1.94 (1.86-2.02)	2.41 (2.21-2.61)
**NIE**			
Model 1	1.00 (1.00-1.00)	1.01 (1.01-1.01)	1.01 (1.01-1.01)
Model 2	1.00 (1.00-1.00)	1.01 (1.00-1.01)	1.02 (1.01-1.02)
**Percentage mediated (%)**			
Model 1	0.46 (0.31-0.60)	2.25 (1.88-2.62)	2.21 (1.81-2.62)
Model 2	0.23 (0.08-0.37)	2.13 (1.74-2.51)	1.97 (1.55-2.39)

Abbreviations: ASD: autism spectrum disorders; NDE: Natural Direct Effect; NIE: Natural Indirect Effect.

Method

Mediation analysis of preterm vs term born were performed by approximating our Cox models with logistic regression, and fitting Natural Effects Models [1, 2]. We used the SAS software Proc Causalmed SAS/Stat 15.2. These analyses are using two logistic regression models, one outcome-model including parental psychiatric history as a predictor for offspring ASD risk and one mediation-model including parental psychiatric history as a predictor for preterm birth. We calculated percentile based 95% bootstrap confidence intervals [3].

From the logistic regression models we calculated three different odds ratios describing the mediating role of preterm birth. In short, the **NDE** for a subject is defined as the difference between the counterfactual outcomes at the two treatment levels when an intervention sets the mediator value to M = M0, which is the natural level of the mediator when there is no treatment. That is, here, outcome difference for term born offspring (or full-term offspring, when examining the mediation of early term) of parental disorders vs no parental disorders. **NIE** for a subject is defined as the difference between the counterfactual outcomes at the two mediator levels at M1 and M0 when an intervention sets the treatment to T = 1. That is, here, outcome difference for preterm minus term born (or early term minus full term) among children of parental disorder at birth. In Model 1, we adjusted for birth year in 5-year categories. In Model 2, we additionally adjusted confounding by maternal and paternal age (years), maternal and paternal education (<9 yrs, 9 yrs, 2nd level 1-2 yrs, 2nd level 3 yrs, PhD, Post 2nd level <3 yrs, Post 2nd level =>3 yrs), all defined at delivery.

References:

1 VanderWeele TJ. A unification of mediation and interaction: a four-way decomposition. Epidemiology. 2014;25(5):749-761. doi:10.1097/EDE.0000000000000121

2 VanderWeele T. Explanation in Causal Inference: Methods for Mediation and Interaction. Oxford University Press; 2015.

3 Efron B, Tibshirani RJ. An Introduction to the Bootstrap. 1st ed. Chapman and Hall/CRC; 1994

eTable 20 Parental psychiatric disorders and offspring ASD risk by offspring sex

**Parental psychiatric disorders**	**ASD (rate)**	**Number of subjects**	**Total person-years**	**Model 1 HR (95% CI)**	**Model 2 HR (95% CI)**
Male offspring					
Neither parent	14,706 (202.1)	653,087	7,277,785	Reference	Reference
Fathers only	1,131 (358.9)	37,748	315,141	2.03 (1.91-2.16)	1.60 (1.51-1.70)
Mothers only	1,977 (406.2)	63,115	486,766	2.41 (2.30-2.53)	2.02 (1.92-2.12)
Both parents	452 (558.6)	12,532	80,912	3.75 (3.41-4.12)	2.34 (2.12-2.58)
Female offspring					
Neither parent	6,241 (90.6)	615,332	6,890,477	Reference	Reference
Fathers only	491 (161.9)	35,805	303,251	2.03 (1.85-2.22)	1.59 (1.45-1.74)
Mothers only	770 (165.6)	59,519	464,875	2.18 (2.02-2.35)	1.83 (1.69-1.97)
Both parents	196 (255.8)	11,782	76,636	3.81 (3.31-4.40)	2.37 (2.05-2.74)

Abbreviations. ASD: Autism Spectrum Disorders; HR: Hazard Ratios; CI: confidence interval.

Note: HRs with 95% CIs were calculated using Cox regression models including interaction term between parental psychiatric history before delivery and offspring sex. Strata-specific effect is shown. Model 1: Adjusted for birth year by cubic natural splines with 5 knots; Model 2: Additionally adjusted for maternal and paternal education (<9 years primary school, 9 years primary school, 1-2 years secondary school, 3 years secondary school, 1-2 years postgraduate education, ≥ 3 years postgraduate education, PhD), income (modeled by ranks as natural cubic splines with five degrees of freedom) and age (as natural cubic splines with five degrees of freedom), all defined at delivery. Incidence rate of ASD per 100,000 person years. Test for interaction term parental psychiatric disorders*offspring sex p=0.16.

eTable 21 Cohort characteristics by any psychiatric disorder in parents in Finland

Characteristics	Neither parent with psychiatric disorders Number of children (%)	Paternal psychiatric disorders only Number of children (%)	Maternal psychiatric disorders only Number of children (%)	Both parents with psychiatric disorders Number of children (%)
Number of individuals	850,247 (83.61%)	67,479 (6.64%)	79,741 (7.84%)	19,455 (1.91%)
Offspring sex (male, %)	434,680 (51.12%)	34,644 (51.34%)	40,678 (51.01%)	9,868 (50.72%)
Follow-up years*	10.7 (5.8-15.5)	8.6 (4.2-13.7)	6.6 (3.2-10.9)	5.8 (2.7-10.0)
Birth year				
1997-2001	238,507 (28.05%)	13,234 (19.61%)	8,196 (10.28%)	1,566 (8.05%)
2002-2006	222,484 (26.17%)	15,827 (23.45%)	15,757 (19.76%)	3,456 (17.76%)
2007-2011	212,342 (24.97%)	18,600 (27.56%)	25,125 (31.51%)	5,969 (30.68%)
2012-2016	176,914 (20.81%)	19,818 (29.37%)	30,663 (38.45%)	8,464 (43.51%)
Offspring ASD	5,954 (0.70%)	644 (0.95%)	767 (0.96%)	283 (1.45%)
Autistic	780 (13.10%)	74 (11.49%)	86 (11.21%)	37 (13.07%)
Asperger’s syndrome	2,195 (36.87%)	216 (33.54%)	249 (32.46%)	82 (28.98%)
Other ASD	2,979 (50.03%)	354 (54.97%)	432 (56.32%)	164 (57.95%)
Age at ASD*	7.8 (4.7-11.0)	7.8 (4.6-11.0)	7.0 (4.3- 9.7)	6.4 (4.0- 9.9)
Preterm birth (<37 week)	45,722 (5.38%)	3,871 (5.74%)	5,442 (6.82%)	1,429 (7.35%)
Maternal age at delivery*	30 (26-33)	28 (24-33)	29 (24-33)	27 (23-32)
Paternal age at delivery*	32 (29-37)	32 (27-37)	32 (27-36)	31 (26-36)
Maternal education**				
Lower secondary education	76,147 (8.96%)	13,957 (20.68%)	17,827 (22.36%)	8,078 (41.52%)
Upper secondary education	344,250 (40.49%)	32,396 (48.01%)	36,220 (45.42%)	8,368 (43.01%)
Post-secondary education	2,856 (0.34%)	236 (0.35%)	250 (0.31%)	60 (0.31%)
Short-cycle tertiary education	127,985 (15.05%)	5,763 (8.54%)	5,135 (6.44%)	729 (3.75%)
Bachelor’s level	157,082 (18.47%)	9,424 (13.97%)	12,074 (15.14%)	1,488 (7.65%)
Master’s level	135,879 (15.98%)	5,468 (8.10%)	7,922 (9.93%)	703 (3.61%)
Doctoral level	6,048 (0.71%)	235 (0.35%)	313 (0.39%)	29 (0.15%)
Paternal education**				
Lower secondary education	109,168 (12.84%)	22,790 (33.77%)	14,540 (18.23%)	8,481 (43.59%)
Upper secondary education	417,811 (49.14%)	33,063 (49.00%)	43,376 (54.40%)	9,001 (46.27%)
Post-secondary education	5,149 (0.61%)	338 (0.50%)	547 (0.69%)	79 (0.41%)
Short-cycle tertiary education	81,872 (9.63%)	2,876 (4.26%)	4,174 (5.23%)	468 (2.41%)
Bachelor’s level	116,249 (13.67%)	4,713 (6.98%)	9,525 (11.94%)	859 (4.42%)
Master’s level	111,040 (13.06%)	3,423 (5.07%)	6,929 (8.69%)	531 (2.73%)
Doctoral level	8,958 (1.05%)	276 (0.41%)	650 (0.82%)	36 (0.19%)

Abbreviations. ASD: autism spectrum disorders; AD: Autistic Disorder; ID: intellectual disability; Q1: 1st quartile (25th percentile), Q3: 3rd quartile (75th percentile); *Median (Q1-Q3). ** Based on the Finnish National Classification of Education 2016, available at (https://www.stat.fi/en/luokitukset/koulutusaste/koulutusaste_1_20160101/. Information of variables were obtained from multiple Finish national registers including Medical Birth Register, Care Register for Health Care, Statistics Finland and the Digital and Population Data Services Authority.

eTable 22 Parental psychiatric disorders before childbirth and risk of offspring ASD in Finland, and across sites

	Finland					Finland + Sweden	
Analysis group	ASD (rate)	Number of subjects	Total person years	Model 1 HR (95% CI)	Model 2 HR (95% CI)	Model 1 HR (95% CI)	Model 2 HR (95% CI)
**Any psychiatric disorder**							
Neither parent	5,954 (66.5)	850,247	8,958,751	Reference	Reference	Reference	Reference
Father only	644 (105.5)	67,479	610,466	1.63 (1.50-1.77)	1.52 (1.40-1.65)	1.90 (1.82 - 1.99)	1.57 (1.50 - 1.64)
Mother only	767 (130.2)	79,741	589,246	2.12 (1.96-2.28)	1.98 (1.84-2.14)	2.29 (2.21 - 2.37)	1.96 (1.89 - 2.03)
Both parents	283 (215.8)	19,455	131,146	3.61 (3.20-4.07)	3.17 (2.80-3.58)	3.72 (3.48 - 3.97)	2.57 (2.40 - 2.75)
**NDD**							
Father only	84 (142.3)	9,558	59,047	2.46 (1.99-3.06)	2.27 (1.82-2.82)	2.83 (2.53 - 3.17)	2.09 (1.87 - 2.35)
Mother only	84 (135.9)	10,201	61,816	2.37 (1.91-2.94)	2.10 (1.68-2.61)	3.42 (3.12 - 3.76)	2.49 (2.26 - 2.74)
Both (different)	80 (282.5)	5,156	28,315	5.12 (4.10-6.39)	4.41 (3.52-5.53)	5.19 (4.59 - 5.87)	3.32 (2.93 - 3.76)
Both (same)	21 (304.3)	1,462	6,901	5.92 (3.86-9.09)	5.15 (3.34-7.94)	5.97 (4.53 - 7.86)	3.70 (2.80 - 4.87)
**Schizophrenia and other non-mood psychotic disorders**							
Father only	48 (150.9)	3,509	31,808	2.33 (1.75-3.09)	2.17 (1.64-2.89)	1.96 (1.61 - 2.38)	1.68 (1.38 - 2.05)
Mother only	55 (167.6)	3,92	32,81	2.63 (2.02-3.43)	2.38 (1.83-3.11)	2.32 (1.96 - 2.75)	2.02 (1.70 - 2.40)
Both (different)	53 (313.2)	2,387	16,924	5.16 (3.94-6.76)	4.46 (3.40-5.85)	4.74 (3.95 - 5.70)	3.40 (2.82 - 4.09)
Both (same)	2 (115.4)	222	1,733	1.85 (0.46-7.39)	1.61 (0.40-6.44)	4.78 (2.57 - 8.88)	2.94 (1.58 - 5.46)
**Mood disorders**							
Father only	153 (113.2)	17,096	135,136	1.81 (1.54-2.12)	1.67 (1.42-1.97)	1.99 (1.82 - 2.17)	1.65 (1.50 - 1.80)
Mother only	353 (147.7)	35,887	239,049	2.48 (2.23-2.77)	2.30 (2.06-2.57)	2.55 (2.40 - 2.70)	2.21 (2.08 - 2.34)
Both (different)	112 (199.9)	8,725	56,018	3.41 (2.82-4.11)	2.97 (2.46-3.59)	3.79 (3.41 - 4.20)	2.64 (2.37 - 2.93)
Both (same)	66 (271.9)	4,086	24,274	4.79 (3.76-6.11)	4.22 (3.30-5.39)	4.98 (4.26 - 5.81)	3.59 (3.07 - 4.19)
**Neurotic/behavioral disorders**							
Father only	437 (107.4)	42,066	406,864	1.64 (1.49-1.80)	1.54 (1.39-1.70)	1.93 (1.83 - 2.05)	1.64 (1.55 - 1.73)
Mother only	493 (131.4)	50,309	375,144	2.13 (1.94-2.33)	2.01 (1.83-2.21)	2.40 (2.30 - 2.50)	2.09 (2.00 - 2.18)
Both (different)	127 (214.4)	8,916	59,247	3.60 (3.02-4.30)	3.14 (2.62-3.75)	3.82 (3.47 - 4.20)	2.63 (2.39 - 2.90)
Both (same)	112 (232.2)	6,859	48,228	3.83 (3.18-4.62)	3.39 (2.81-4.10)	4.25 (3.83 - 4.72)	2.99 (2.69 - 3.32)
**Substance use**							
Father only	150 (104.5)	15,86	143,581	1.61 (1.37-1.90)	1.43 (1.21-1.69)	1.92 (1.79 - 2.05)	1.46 (1.36 - 1.56)
Mother only	89 (120.0)	9,981	74,171	1.94 (1.57-2.39)	1.72 (1.40-2.13)	2.22 (2.05 - 2.40)	1.69 (1.56 - 1.83)
Both (different)	95 (225.4)	6,202	42,156	3.74 (3.05-4.58)	3.19 (2.59-3.91)	3.83 (3.45 - 4.26)	2.57 (2.31 - 2.86)
Both (same)	38 (175.6)	2,876	21,64	2.83 (2.06-3.89)	2.34 (1.70-3.23)	2.92 (2.52 - 3.39)	1.76 (1.51 - 2.04)
**Other**							
Father only	9 (89.2)	1,268	10,093	1.40 (0.73-2.69)	1.32 (0.69-2.55)	1.54 (1.04 - 2.29)	1.28 (0.87 - 1.90)
Mother only	22 (107.1)	2,917	20,547	1.74 (1.14-2.64)	1.63 (1.07-2.47)	2.40 (1.84 - 3.11)	1.97 (1.51 - 2.56)
Both (different)	14 (181.7)	1,201	7,706	3.05 (1.81-5.16)	2.66 (1.57-4.51)	3.80 (2.74 - 5.26)	2.57 (1.85 - 3.57)
Both (same)	2 (324.2)	106	617	5.71 (1.45-22.54)	5.17 (1.31-20.40)	-*	-*

Abbreviations. ASD: Autism Spectrum Disorders; NDD: neurodevelopmental disorders, emotional and behavioral disorders of childhood origin and intellectual disability; HR: Hazard Ratios; CI: confidence interval; Both (different): one parent had the specified disorder and the other parent had any other psychiatric disorder; Both (same): both parents had the same specific disorder.

Note: HRs with 95% CIs were calculated using Cox regression models. Model 1: Adjusted for birth year by cubic natural splines with 5 knots; Model 2: Additionally adjusted for maternal and paternal education (<9 years primary school, 9 years primary school, 1-2 years secondary school, 3 years secondary school, 1-2 years postgraduate education, ≥ 3 years postgraduate education, PhD), and age (as natural cubic splines with five degrees of freedom), all defined at delivery. Incidence rate of ASD per 100,000 person years. The reference group of all subgroups was offspring of parents without any psychiatric disorder. The table presents the risk estimates for ASD in Finland, as well as meta-estimates across Finland and Sweden [1]. For subgroup of offspring born to both parents with other psychiatric disorders, the risk estimates are missing due to no ASD cases in Swedish sample.

References:

1. Rao CR. Linear Statistical Inference and Its Applications, 2nd Edition. New York: John Wiley & Sons, Inc., 1973.

eTable 23 Number of co-occurring psychiatric disorders in mothers and fathers and risk of ASD in the offspring in Finland

Number of co-occurring psychiatric disorders	**ASD (rate)**	**Number of subjects**	**Total person-years**	**Model 1 HR (95% CI)**	**Model 2 HR (95% CI)**
Paternal disorders					
None	6,721 (70.4)	929,988	9,547,997	Reference	Reference
One category [1]	624 (113.0)	62,410	552,217	1.52 (1.40-1.65)	1.44 (1.32-1.56)
Two categories	206 (147.9)	17,339	139,319	1.88 (1.64-2.16)	1.72 (1.50-1.98)
≥Three categories	97 (193.7)	7,185	50,075	2.34 (1.91-2.87)	2.12 (1.73-2.60)
Maternal disorders					
None	6,598 (69.0)	917,726	9,569,217	Reference	Reference
One category	659 (132.5)	64,681	497,332	1.95 (1.80-2.11)	1.85 (1.71-2.01)
Two categories	274 (168.4)	24,588	162,670	2.50 (2.21-2.82)	2.31 (2.04-2.61)
≥Three categories	117 (193.7)	9,927	60,390	2.78 (2.31-3.35)	2.51 (2.08-3.02)

Abbreviations. ASD: Autism Spectrum Disorders; HR: Hazard Ratios; CI: confidence interval.

Note: HRs with 95% CIs were calculated using Cox regression models. Model 1: Adjusted for birth year by cubic natural splines with 5 knots and any psychiatric history in the opposite parent; Model 2: Additionally adjusted for maternal and paternal education (<9 years primary school, 9 years primary school, 1-2 years secondary school, 3 years secondary school, 1-2 years postgraduate education, ≥ 3 years postgraduate education, PhD), and age (as natural cubic splines with five degrees of freedom), all defined at delivery. Incidence rate of ASD per 100,000 person years. The co-occurring number of psychiatric diagnoses in different major categories (Neurodevelopmental disorders, emotional and behavioral disorders of childhood origin and intellectual disability; Schizophrenia and other non-mood psychotic disorders; Mood disorders; Neurotic/behavioral disorders; Psychoactive substance use; Other/unspecific psychiatric disorders).

eFigures

eFigure 1 Examples of risk patterns of parental psychiatric disorders on offspring ASD risk

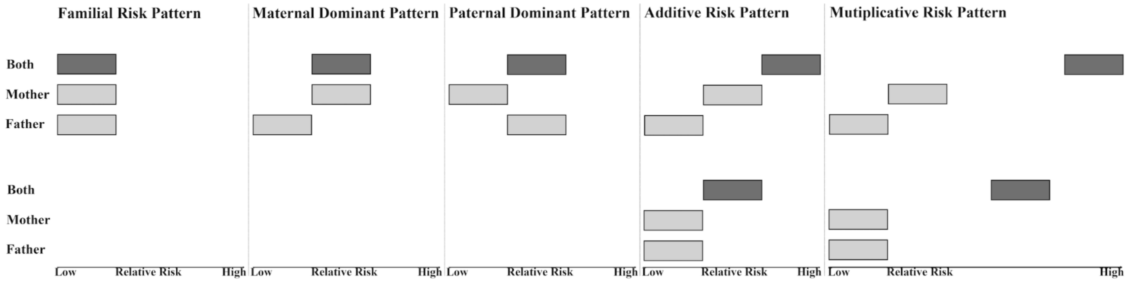

Abbreviations. ASD: Autism Spectrum Disorders

Note: Boxes are representing relative risks of ASD in the offspring of parents with psychiatric disorders, compared to the offspring of neither parents with psychiatric disorders. Light gray: paternal and maternal diagnosis, dark gray: diagnosis in both parents. X-axis: relative risks from low risk to high risk. The Familial Risk Pattern: mothers and fathers contributed equally, with a similar or higher combined effect of both parents; Maternal Dominant Pattern: Maternal effect is higher than paternal effect; Paternal Dominant Pattern: Paternal effect is higher than maternal effect; Additive Risk Pattern: the combined effect of both parents is best approximated by the sum of the parental risks; Multiplicative Risk Pattern: the combined effect of both parents is best approximated by the product of the parental risks.

eFigure 2 Flow diagram illustrating the identification of the study cohort

Swedish cohort

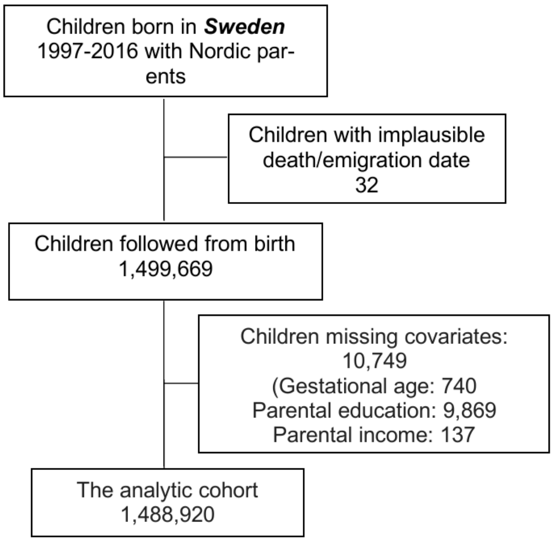

Finnish cohort

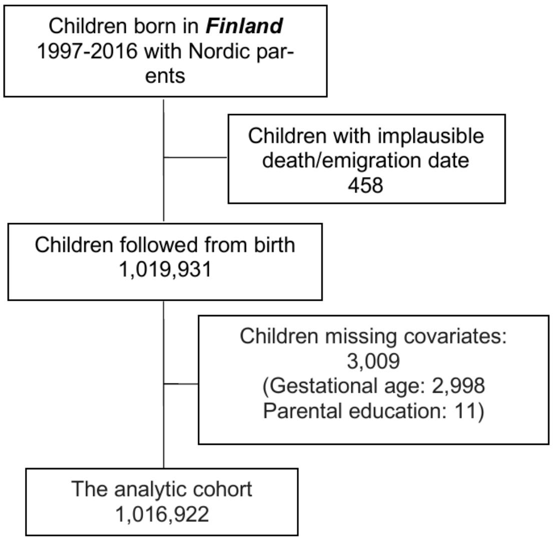

eFigure 3 Adjusted Inverse Kaplan-Meier curves for ASD by co-occurring psychiatric disorders in parents in Sweden

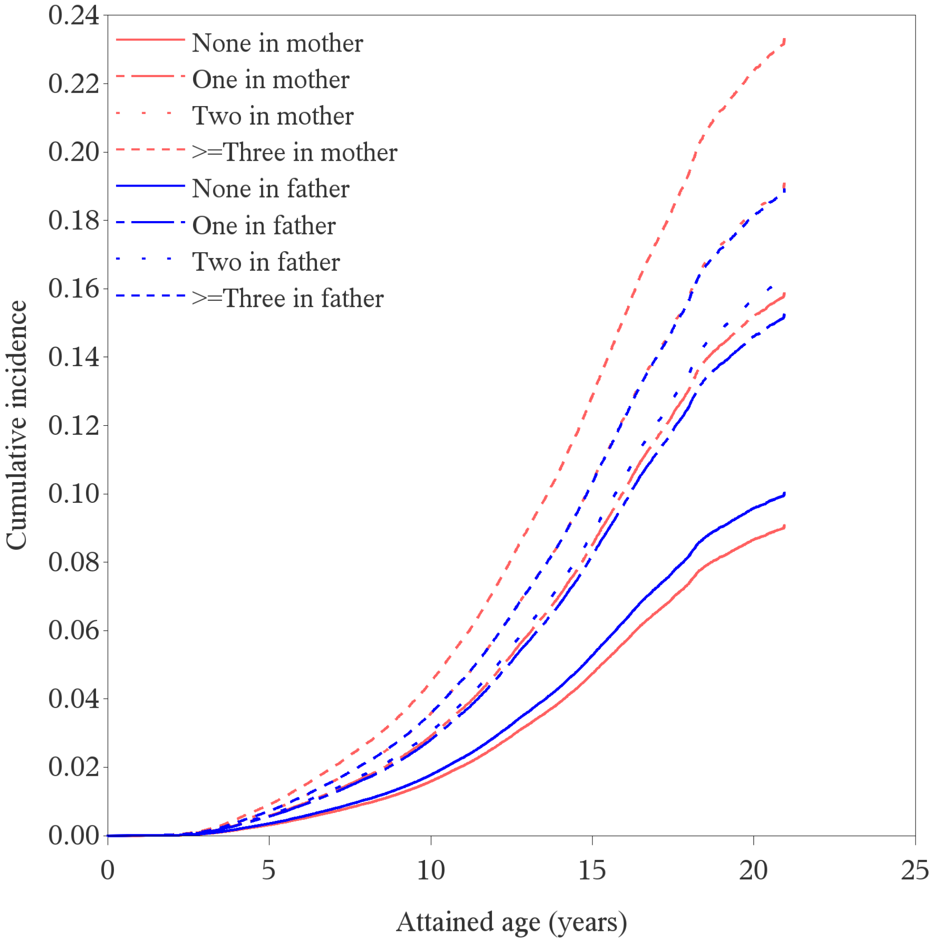

Abbreviations: ASD: autism spectrum disorder.

Note: Inverse Kaplan-Meier curves for cumulative incidence of ASD in the offspring, comparing mothers and fathers with any psychiatric disorder and without psychiatric disorder, by the co-occurring number of psychiatric disorders in different six major categories (Neurodevelopmental disorders, emotional and behavioral disorders of childhood origin and intellectual disability; Schizophrenia and other non-mood psychotic disorders; Mood disorders; Neurotic/behavioral disorders; Psychoactive substance use; Other/unspecific psychiatric disorders), adjusting for birth year (5-year intervals), maternal and paternal age (<20 years, 20-40, >40 years), maternal and paternal education (<10 years, 10-12years, >12 years of schooling), and psychiatric history of the other parent (yes/no).

eFigure 4 Scaled Schoenfeld residual plot to assess proportional hazards assumption

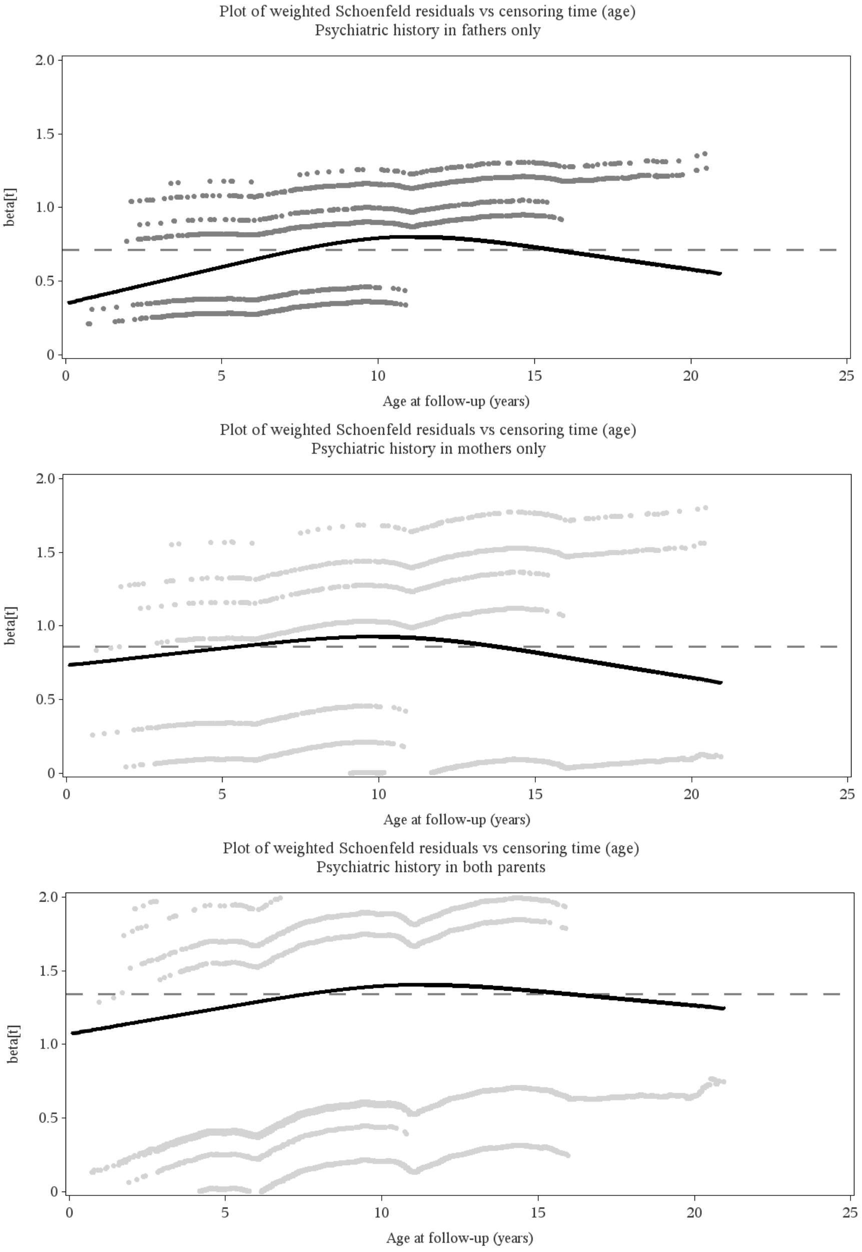

Note: The proportional hazards assumption of the Cox regression was visually examined by Schoenfeld residuals, where a lack of systematic patterns or trends over time indicates adherence to the assumption. The figure displays age-specific log hazard ratios for the risk of ASD. The age-specific hazard ratios are estimated by the weighted Schoenfeld residuals on the y-axis and x-axis, respectively. The graph displays a smoother that represents the average expected risk at each age.

eFigure 5 Inverse Kaplan-Meier curves for ASD by co-occurring psychiatric disorders in parents in Finland

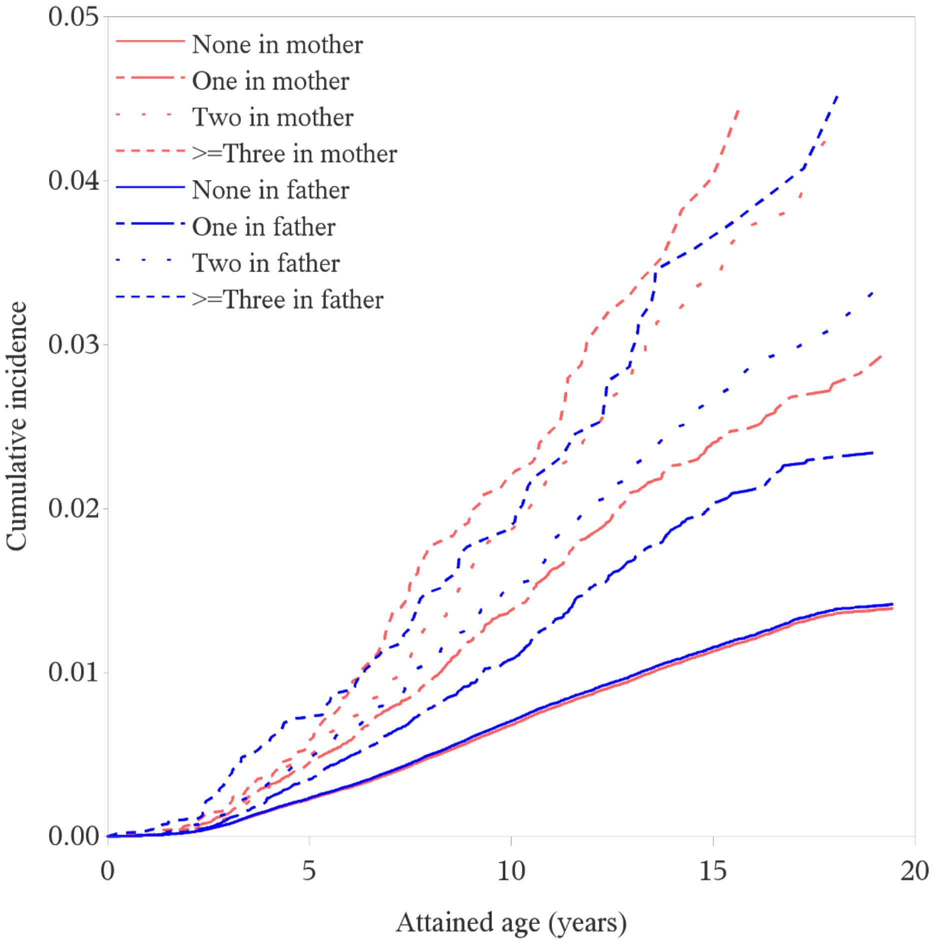

Abbreviations: ASD: Autism Spectrum Disorder.

Note: Inverse Kaplan-Meier curves for cumulative incidence of ASD in the offspring, comparing mothers and fathers with any psychiatric disorder and without psychiatric disorder, by the co-occurring number of psychiatric disorders in different six major categories (Neurodevelopmental disorders, emotional and behavioral disorders of childhood origin and intellectual disability; Schizophrenia and other non-mood psychotic disorders; Mood disorders; Neurotic/behavioral disorders; Psychoactive substance use; Other/unspecific psychiatric disorders).
