# Supplementary material for: Association between parental psychiatric disorders and risk of offspring autism spectrum disorder: a Swedish and Finnish population-based cohort study
Source: Lancet Reg Health Eur. 2024 Apr 23;40:100902. doi: 10.1016/j.lanepe.2024.100902 (PMC11059471; doi:10.1016/j.lanepe.2024.100902)
Supplement: SAP [file mmc2.pdf]

Parental psychiatric diagnoses at the time of delivery, preterm birth and risk of autism in the offspring

Background

Autism Spectrum Disorders (ASD), is a chronic neurodevelopmental disorder that affects 1-2% of children worldwide[1-3]. ASD have been considered highly heritable [4,5], however environmental factors also account for some of the variance to develop ASD [6].

Previous clinical studies suggest that psychiatric disorders are more common among relatives of children with ASD. This has led to the assumption that ASD and several other psychiatric disorders may share common genetic/familial factors (Daniels et al., 2008). Several population-based study have examined the association between parental psychiatric disorders and offspring ASD, however, with methodological limitations, e.g., did not separate maternal and paternal psychiatric disease [7-9], had no subgroup analysis of different psychiatric disorders [10,11], conditioned on parental psychiatric diagnoses in the future (e.g., after offspring ASD) [8,9,11-13]. Besides, studies on the ASD risk in relation to the most of which onset of parental psychiatric diagnosis are few, most of which did not use an appropriate statistical method to address the immortal bias, i.e. a time-varying model [12,14].

Preterm birth, as a well-established risk factor for ASD[27], has also been reported more common in mothers with psychiatric disorders [15]. Previous studies on maternal psychiatric disorders and risk of preterm delivery mostly focus on mood disorders and PTSD[17-20], since disruptions in homeostasis have been thought to increase the likelihood of preterm birth [16]. There are a few studies on overall psychiatric history, however, estimates of specific risk for psychiatric subtypes are lacking[21-24]. To date, studies on psychiatric history in fathers and risk of preterm birth in the offspring are sparse.

On top of it, the role of preterm birth, in the association between parental psychiatric history and offspring ASD risk has not been thoroughly examined. To our best knowledge, only one cohort study analyzed the role of preterm birth in the association, however not cover the overall psychiatric disorders and did not use an up-to-date statistical method for mediation analysis [25].

In this study, we aim to examine the association between psychiatric history in parents and risk of ASD in offspring, particularly the role of preterm birth in the association. We opted to take a prevention-driven perspective, in order to generate evidence on high-risk groups for ASD, which might inform early detection and intervention efforts in the further.

1 Objective

To estimate the association between (1) maternal and paternal psychiatric history and risk of preterm birth; (2) maternal and paternal psychiatric history and risk of offspring ASD; (3) to examine the role of preterm birth in the association.

1.1 Proposed authors in order

Weiyao Yin, Kari Risnes, Eero Kajantie, Ulrika Åden, Martina Persson, Michael Silverman, Sven Sandin

1.2 Target publication

JAMA

1.3 Time plan

- Final analysis plan 7th Feb.
- Preliminary analysis and draft 30th April
- First submission 15th Jun

2 Methods

2.1 Study population

All children born alive in Sweden between January 1, 1995 and December 31, 2015. Data is derived from Swedish national registers, including the Swedish Medical Birth Register, the Swedish Multi-generation Register, the National Patient Register, the Education Register, and the Statistics Sweden Total Population Register.

2.1.1 Exclusion criteria for study population

The following subjects will be excluded:

Children born outside Sweden

Missing link to mother and father

Children born to parents born outside the Nordic countries (i.e., Sweden, Denmark, Norway and Finland).

Children died, emigrated or had ASD before age 2.

2.2 DATA MANAGEMENT

Exposure

The primary exposure is any incident psychiatric diagnosis in mothers and fathers, at birth of the child. We identify the first psychiatric diagnosis registered in the NPR.

The secondary exposures include psychiatric subtypes: six categories: psychoactive substance use, schizophrenia spectrum, mood disorders, neurotic, stress-related, behavioral and personality disorders, disorders usually occurred in childhood and adolescence, and other psychiatric disorders except for the mentioned above; certain subtypes under each category. Individual with several psychiatric diagnoses can contribute to different diagnostic categories.

	ICD-10	ICD-9	ICD-8
Overall mental illness	F10-F99	295, 296,297,298,299,300,301, 302, 303,304,305 306,307, 308, 309, 311,312,313,314,315, 316, 317,318,319	295,296,297,298,299,300,301,302, 303,304, 305, 306, 307, 308,310,311,312,313,314,315
**Psychoactive substance use**	F10-F19	303,304,305	303,304
**Schizophrenia and other paranoid psychoses**	F20–29	295,297,298C-X	295, 297, 298.2, 298.3, 298.9, 299
**Mood disorders**	F30-39	296, 298A, 298B, 300E, 311	296, 298.0, 298.1, 300.4
Depression	F32 F33 F341 F348 F349	296B, 300E, 311	296.0, 298.0, 300.4
Bipolar	'F30,'F31,'F340	296CDE	296.1, 296.2, 296.3, 296.8
**Neurotic, stress-related, behavioral and personality disorders**	F40-F48, F50-59, F60-F69	300–302 (excluding 300E), 306, 307, 308, 309	300–302 (excl. 300.4), 305, 306, 307
Anxiety	F40 F41	300A, 300C	300.0,300.2
OCD	F42	300D	300.3
Stress-related disorder	F43	308,309	
Somatoform disorder	F45	306	305, 306 (excl 306.5)
Eating disorder	F50	307F	306.5
Personality disorder	F60	301	301
**Disorders usually occured in childhood and adolescence**	F70-F79, F80-F89, F90-98	299, 312-315, 317-319	308, 310-315
Intellectual disability	F70-F79	317, 318, 319	310, 311, 312, 313, 314, 315
Disorders of psychological and behavioural development			
ASD	F840, F841, F845, F848, F849	299A	
ADHD	F90	314	
**Other psychiatric disroders**	F99	316	

ASD

The primary outcome is any ASD diagnosis and ASD subtypes registered in the National Patient Register.

	ICD-10
**Autism diagnoses**	
ASD [1]	F840, F841, F843, F845, F848, F849
AD	F840
Asperger's syndrome	F845

The secondary outcome is ASD comorbid with ID registered in the NPR.

ID Type	IQ Range	ICD 10
Any ID		F70-79
Mild intellectual disability	52-69	F70
Moderate intellectual disability	36-51	F71
Severe intellectual disability	20-35	F72
Profound intellectual disability	≤ 19	F73
Other intellectual disability		F78
Unspecified intellectual disability		F79

Preterm birth

Gestational age in weeks (as a continuous variable) is derived from the Medical Birth Register. We categorize gestational age into preterm (<37 weeks; very preterm <32 and moderate to late preterm 32-36 weeks) and term (≥37 weeks) birth.

**Preterm**	< 37 weeks
Very preterm	<32
Moderate to late preterm	32 - 36
**Term**	≥37

If possible

Preterm	< 37 weeks
Extremely preterm	< 28 weeks
Very preterm	28 - 31
Moderate to late preterm	32 - 36
Term	37-41
Post-term	>41

Firstly, we will examine the association between parental psychiatric history and risk of preterm birth. Next, we will examine the role of preterm birth in the association between parental psychiatric history and offspring ASD, as a mediator or risk modifier.

Covariates

<u>Potential confounding factors</u>

Continuous variables: birth year, maternal and paternal income (SEK), maternal and paternal age (Hultman et al., 2011, Sandin et al., 2012). Categorical variable: maternal and paternal educational level (completed years in school, 0-9, 10-12, >12 years).

<u>Potential mediators or modifiers</u>

Categorical covariates: smoking during pregnancy (yes, no) (Hultman et al., 2002, Maughan et al., 2004; Larsson et al., 2009), maternal body mass index (BMI; kg/m^2) at first antenatal visit (underweight <18.5 kg/m^2, normal weight 18.5-24.9 kg/m^2, overweight 25-29.9 kg/m^2, obese >30 kg/m^2) (Bennedsen et al., 1999; Hultman et al., 2002; Lampi et al., 2012).

2.3 Plans for statistical analyzes

2.3.1 Statistical models and statistical analyzes

Individuals missing data in gestational age and parental educational attainment will be further excluded.

We quantified the association between parental psychiatric diagnosis before delivery and offspring ASD risk by incidence rate ratios (RR) and associated two-sided 95% confidence interval (CI), from Poison regression models, together with ASD incidence rate (cases per 100,000 person years). Each child was followed from age two until the first ASD diagnosis, emigration, death, or the 31st December 2017, whichever came first.

In primary analysis, we examine overall psychiatric diagnosis and by subtypes in mothers and fathers, separately, before birth of the child, by fitting models with increasing adjustment for potential confounding. Continuous variables were modeled as natural cubic splines [26], with five degrees of freedom to allow for non-linear relations. In the "crude" model, we adjusted for age at follow-up and birth year. Next, we adjusted for maternal and paternal income, educational level and age at delivery. Finally, we additionally adjusted for any paternal psychiatric history before delivery when maternal P.hist is the exposure and vice versa.. Inverse Kaplan-Meier (KM) curves were used to depict the cumulative incidence of ASD.

Next, we quantified the association between parental psychiatric history before delivery and preterm delivery (yes/no) by odds ratio (OR) and associated two-sided 95% confidence intervals (CI), in the crude model (adjusted for birth year) and the adjusted model (additionally adjusted for parental education).

Finally, the role of gestational age at birth in the association between parental psychiatric disorders and offspring ASD will be further examined. We will repeat the analyses in subgroups of children born term (≥37weeks) and preterm (<37 weeks), very preterm (<32) and moderate to late preterm (32-36 weeks). Next, we performed a mediation analysis of preterm vs term born by approximating our Poison models with logistic regression, and fitting Natural Effects Models [1, 2]. We used the SAS software Proc Causalmed SAS/Stat 15.2. These analyses are using two logistic regression models, one outcome-model including parental psychiatric disorders as a predictor for offspring ASD risk and one mediation-model including parental psychiatric disorders as a predictor for preterm birth. We calculated percentile based 95% bootstrap confidence intervals [3].

All statistical tests were performed on the two-sided 5% level of significance, corresponding to a two-sided 95% CI. Robust standard errors ("sandwich estimator") adjust for potential correlations between siblings [27].

Secondary analysis

(1) To examine ASD risk in relation to onset parental psychiatric diagnosis, we included maternal and paternal psychiatric diagnosis as a time-varying exposure in Poisson regression model. Here, we compared the ASD incidence in offspring of parents with first psychiatric diagnosis before conception (the date of delivery - gestational length), during pregnancy, in postpartum period (the first year after delivery) and from second year after delivery and onwards, compared to offspring of parents without any psychiatric disorder. (2) We

performed the similar mediation analysis for SGA vs non-SGA. Small for gestational age (SGA, ≤10th percentile of population), appropriate for gestational age (AGA, 11-89th percentile population), and large for gestational age (LGA, ≥90th percentile of population)[21, 22]. (3) By comorbid psychiatric diseases, only one, two or three and more psychiatric categories. (4) By ASD subtypes, i.e. AD and Asperger's syndrome. (5) By ASD comorbid with ID and without ID. (6) Separately by offspring sex. (7) Separate analysis by spontaneous and medical preterm birth. (8) We will further include other potential modifiers in the model, including maternal smoking during pregnancy (yes/no) and maternal BMI at first prenatal visit. If the results are similar, it means that maternal BMI and smoking are not on the casual pathway. If it has certain impact, we need to extimate that appropriately, e.g. using marginal structure depending on weights.
Statistical analyses were performed using SAS software version 9.4 (SAS institute Inc, Cary, NC, USA). The present study was approved by Regional Ethical Review Board in Stockholm, Sweden.

References

1. Buxbaum JD HP. The Neuroscience of Autism Spectrum Disorders. Academic Press. Accessed September 12, 2013. (http://www.sciencedirect.com/science/book/9780123919243)
2. Baxter AJ, Brugha TS, Erskine HE, Scheurer RW, Vos T, Scott JG. The epidemiology and global burden of autism spectrum disorders. Psychological medicine. Feb 2015;45(3):601-13. doi:10.1017/s003329171400172x
3. Elsabbagh M, Divan G, Koh YJ, et al. Global prevalence of autism and other pervasive developmental disorders. Autism research : official journal of the International Society for Autism Research. Jun 2012;5(3):160-79. doi:10.1002/aur.239
4. Sandin S, Lichtenstein P, Kuja-Halkola R, Larsson H, Hultman CM, Reichenberg A. The familial risk of autism. Jama. May 7 2014;311(17):1770-7. doi:10.1001/jama.2014.4144
5. Bai D, Yip BHK, Windham GC, et al. Association of Genetic and Environmental Factors With Autism in a 5-Country Cohort. JAMA psychiatry. Oct 1 2019;76(10):1035-1043. doi:10.1001/jamapsychiatry.2019.1411
6. Yip BHK, Bai D, Mahjani B, et al. Heritable Variation, With Little or No Maternal Effect, Accounts for Recurrence Risk to Autism Spectrum Disorder in Sweden. Biological psychiatry. Apr 1 2018;83(7):589-597. doi:10.1016/j.biopsych.2017.09.007
7. Larsson HJ, Eaton WW, Madsen KM, et al. Risk factors for autism: perinatal factors, parental psychiatric history, and socioeconomic status. Am J Epidemiol. May 15 2005;161(10):916-25; discussion 926-8. doi:10.1093/aje/kwi123
8. Xie S, Karlsson H, Dalman C, et al. Family History of Mental and Neurological Disorders and Risk of Autism. JAMA Netw Open. Mar 1 2019;2(3):e190154. doi:10.1001/jamanetworkopen.2019.0154
9. Sullivan PF, Magnusson C, Reichenberg A, et al. Family history of schizophrenia and bipolar disorder as risk factors for autism. Arch Gen Psychiatry. Nov 2012;69(11):1099-1103. doi:10.1001/archgenpsychiatry.2012.730
10. Lauritsen MB, Pedersen CB, Mortensen PB. Effects of familial risk factors and place of birth on the risk of autism: a nationwide register-based study. J Child Psychol Psychiatry. Sep 2005;46(9):963-71. doi:10.1111/j.1469-7610.2004.00391.x
11. Daniels JL, Forssen U, Hultman CM, et al. Parental psychiatric disorders associated with autism spectrum disorders in the offspring. Pediatrics. May 2008;121(5):e1357-62. doi:10.1542/peds.2007-2296
12. Chen LC, Chen MH, Hsu JW, et al. Association of parental depression with offspring attention deficit hyperactivity disorder and autism spectrum disorder: A nationwide birth cohort study. J Affect Disord. Dec 1 2020;277:109-114. doi:10.1016/j.jad.2020.07.059
13. Liang CS, Bai YM, Hsu JW, et al. Associations of parental mental disorders and age with childhood mental disorders: a population-based cohort study with four million offspring. Eur Child Adolesc Psychiatry. Nov 21 2021;doi:10.1007/s00787-021-01914-3
14. Chen MH, Pan TL, Bai YM, et al. Postpartum Depression and Psychosis and Subsequent Severe Mental Illnesses in Mothers and Neurodevelopmental Disorders in Children: A Nationwide Study. J Clin Psychiatry. Jul 27 2021;82(4)doi:10.4088/JCP.20m13735

15. Männistö T, Mendola P, Kiely M, et al. Maternal psychiatric disorders and risk of preterm birth. Ann Epidemiol. Jan 2016;26(1):14-20. doi:10.1016/j.annepidem.2015.09.009
16. Traylor CS, Johnson JD, Kimmel MC, Manuck TA. Effects of psychological stress on adverse pregnancy outcomes and nonpharmacologic approaches for reduction: an expert review. Am J Obstet Gynecol MFM. Nov 2020;2(4):100229. doi:10.1016/j.ajogmf.2020.100229
17. Gavin AR, Holzman C, Siefert K, Tian Y. Maternal depressive symptoms, depression, and psychiatric medication use in relation to risk of preterm delivery. Womens Health Issues. Sep-Oct 2009;19(5):325-34. doi:10.1016/j.whi.2009.05.004
18. Malm H, Sourander A, Gissler M, et al. Pregnancy Complications Following Prenatal Exposure to SSRIs or Maternal Psychiatric Disorders: Results From Population-Based National Register Data. Am J Psychiatry. Dec 2015;172(12):1224-32. doi:10.1176/appi.ajp.2015.14121575
19. Yonkers KA, Smith MV, Forray A, et al. Pregnant women with posttraumatic stress disorder and risk of preterm birth. JAMA Psychiatry. Aug 2014;71(8):897-904. doi:10.1001/jamapsychiatry.2014.558
20. Shaw JG, Asch SM, Kimerling R, Frayne SM, Shaw KA, Phibbs CS. Posttraumatic stress disorder and risk of spontaneous preterm birth. Obstet Gynecol. Dec 2014;124(6):1111-1119. doi:10.1097/aog.0000000000000542
21. Mongan D, Lynch J, Hanna D, et al. Prevalence of self-reported mental disorders in pregnancy and associations with adverse neonatal outcomes: a population-based cross-sectional study. BMC Pregnancy Childbirth. Nov 8 2019;19(1):412. doi:10.1186/s12884-019-2572-4
22. Kelly RH, Russo J, Holt VL, et al. Psychiatric and substance use disorders as risk factors for low birth weight and preterm delivery. Obstet Gynecol. Aug 2002;100(2):297-304. doi:10.1016/s0029-7844(02)02014-8
23. Kang-Yi CD, Kornfield SL, Epperson CN, Mandell DS. Relationship Between Pregnancy Complications and Psychiatric Disorders: A Population-Based Study With a Matched Control Group. Psychiatr Serv. Mar 1 2018;69(3):300-307. doi:10.1176/appi.ps.201700097
24. Atkinson KD, Nobles CJ, Kanner J, Männistö T, Mendola P. Does maternal race or ethnicity modify the association between maternal psychiatric disorders and preterm birth? Ann Epidemiol. Apr 2021;56:34-39.e2. doi:10.1016/j.annepidem.2020.10.009
25. McCoy BM, Rickert ME, Class QA, Larsson H, Lichtenstein P, D'Onofrio BM. Mediators of the association between parental severe mental illness and offspring neurodevelopmental problems. Ann Epidemiol. Sep 2014;24(9):629-34, 634.e1. doi:10.1016/j.annepidem.2014.05.010
26. Benedetti A, Abrahamowicz M. Using generalized additive models to reduce residual confounding. Stat Med. Dec 30 2004;23(24):3781-801. doi:10.1002/sim.2073
27. Lin DY, Wei L-J. The robust inference for the Cox proportional hazards model. Journal of the American statistical Association. 1989;84(408):1074-1078.

Tables

Table 1 Cohort characteristics

	No parental history	Maternal history	Paternal history
	Number of children (%)	Number of children (%)	Number of children (%)
Number of individuals			
Follow-up years (median, Q1-Q3)			
Offspring sex (male, %)			
Offspring ASD (yes, %)			
Birth year			
1995-1999			
2000-2004			
2005-2009			
2010-2015			
Gestational week			
<37 week			
<32 week			
32-36 week			
37-41 week			
>41 weeks			
Maternal age at delivery, years (median, Q1-Q3)			
Paternal age at delivery, years (median, Q1-Q3)			
Maternal yearly income (median, Q1-Q3)			
Paternal yearly income (median, Q1-Q3)			
Maternal education, (years in school)			
0-9 years			
10-12 years			
>12 years			
Paternal education (years in school)			
0-9 years			
10-12 years			
>12 years			

Note: Abbreviations. Q1: 1st quartile (25th percentile), Q3: 3rd quartile (75th percentile)

Table 2 Relative risk of preterm birth in offspring of mothers and fathers with psychiatric disorders before delivery

	Maternal P.Hist				Paternal P.Hist			
P.Hist before delivery	PTB (%)	Total subjects	Crude	Adjusted	PTB (%)	Total subjects	Crude	Adjusted
Any P.shit								
No P.Hist								
Psychiatric diagnoses in categories								
Psychoactive substance use								
Schizophrenia and other paranoid psychoses								
Mood disorders								
Depression								
Bipolar								
Neurotic, stress-related, behaviural and personality disorders								
Anxiety								
OCD								
Stress-related disorder								
Somatoform disorder								
Eating disorder								
Personality disorder								
Disorders usually occured in childhood and adolescence								
Intellectual disability								
Disorders of psychological and behavioural development								
ASD								
ADHD								
Other psychiatric disroders								

Note: Abbreviations. PTB: Preterm Birth; P.Hist: Psychiatric History. Odds ratios (OR) with 95% confidence interval was calculated using Logistic regression models. Crude: Adjusted for birth year by cubic natural splines with 5 knots; Adjusted: Additionally adjusted for maternal and paternal education (0-9, 10-12, >12 years),income (SEK) and age (years) at delivery.

Table 3 Relative risk of ASD in offspring of mothers and fathers with psychiatric disorders before delivery

	Maternal P.Hist XX children				Paternal P.Hist XX children			
P.Hist before delivery	ASD (rate[1])	Person years	Crude	Adjusted	ASD (rate)	Person years	Crude	Adjusted
Overall								
No P.Hist								
Psychiatric diagnoses in categories								
Psychoactive substance use								
Schizophrenia and other paranoid psychoses								
Mood disorders								
Depression								
Bipolar								
Neurotic, stress-related, behaviural and personality disorders								
Anxiety								
OCD								
Stress-related disorder								
Somatoform disorder								
Eating disorder								
Personality disorder								
Disorders usually occured in childhood and adolescence								
Intellectual disability								
Disorders of psychological and behavioural development								
ASD								
ADHD								
Other psychiatric disroders								

Note: Abbreviations. ASD: Autism Spectrum Disorders; P.Hist: Psychiatric History. Relative risk (rate ratio RR) with 95% confidence interval was calculated using Poisson regression models with robust standard errors. Crude: Adjusted for birth year by cubic natural splines with 5 knots; Adjusted: Additionally adjusted for maternal and paternal education (0-9, 10-12, >12 years),income (SEK) and age (years) at delivery.. [1]Incidence rate of ASD per 100,000 person years.

Table 4 Subgroup and Mediation analysis by preterm birth

**Subgroup analysis**	ASD (rate [1])	Person years	Crude RR (95% CI)	Adjusted RR (95% CI)
Preterm (<37 weeks)				
Maternal P.Hist				
No maternal P.Hist				
Very preterm (<32 weeks)				
Maternal P.Hist				
No maternal P.Hist				
Moderate to late preterm (32-36 weeks)				
Maternal P.Hist				
No maternal P.Hist				
Term (≥37 weeks)				
Maternal P.Hist				
No maternal P.Hist				
**Mediation analysis**	Crude	Adjusted		
Total Effect, OR (95%)				
Controlled Direct Effect, OR (95%)				
Natural Indirect Effect, OR (95%)				
Proportion Mediation, % (95%)				

Note: Abbreviations. ASD: Autism Spectrum Disorders; P.Hist: Psychiatric History; RR: rate ratio; OR: odds ratio; CI: confidence interval. For subgroup analysis, incidence rate ratio with 95% confidence interval was calculated using Poisson regression models with robust standard errors. For mediation analysis, odds ratio with 95% confidence interval was calculated by approximating our poisson models with logistic regression, and fitting Natural Effects Models [1, 2]. These analyses are using two logistic regression models, one outcome-model including maternal P.Hist as a predictor for offspring ASD risk and one mediation-model including maternal P.Hist as a predictor for preterm birth. "Total Effect" is the OR of ASD comparing preterm born offspring of mothers with P.Hist to term born offspring of mothers without P.Hist, i.e. the OR comparing assumed highest risk group to lowest risk group. "Controlled Direct Effect" is the

OR of ASD comparing offspring of mothers with P.Hist to offspring of mothers without P.His, when the preterm covariates are assigned the same value, e.g. term. "Natural Indirect Effect" is the OR of ASD comparing offspring born preterm to offspring born term assuming all are born to mothers diagnosed with P.Hist. We calculated percentile based 95% bootstrap confidence intervals [3]. Crude: Adjusted for birth year by cubic natural splines with 5 knots; Adjusted: Additionally adjusted for maternal and paternal education (categorized), maternal and paternal age at delivery (by cubic natural splines), maternal and paternal income (by natural cubic splines). [1]Incidence rate of ASD per 100,000 person years.

Figure 1 Inverse Kaplan-Meier curves for offspring of parents with and without psychiatric disorders before delivery, by ASD subtypes and comorbidity with ID

Maternal

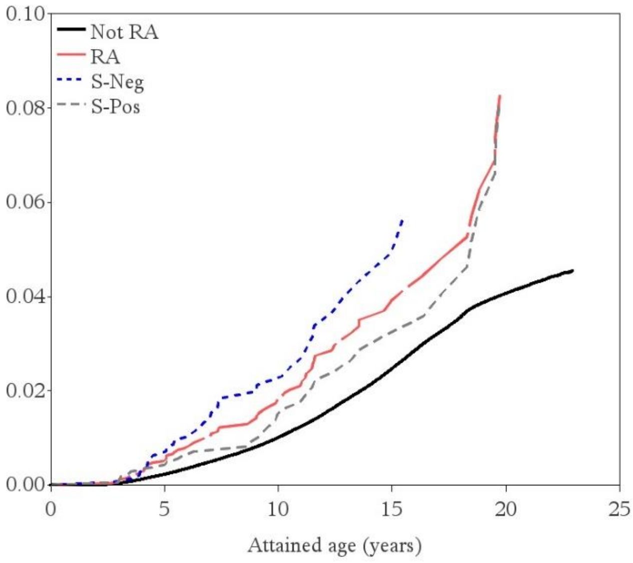

Paternal

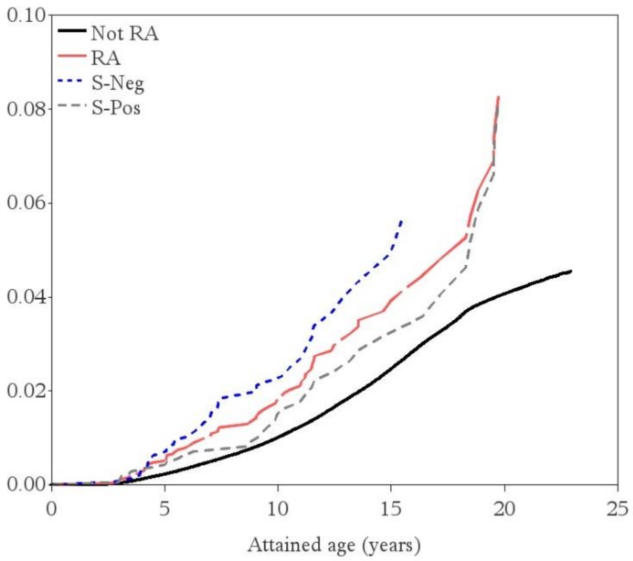

Note: ASD: Autism Spectrum Disorder; AD: autistic disorders. Inverse Kaplan-Meier curves for the cumulative incidence of ASD in the offspring, comparing mothers and fathers with any psychiatric disorder and without psychiatric disorder, by overall ASD, AD, Asperger's syndrome, ASD comorbid with ID and ASD comorbid without ID.

Supplementary tables

Table 1 Risk of ASD in offspring in relation to onset of parental psychiatric diagnosis

	Maternal P.Hist				Paternal P.Hist			
Analysis group	ASD (rate[1])	Person years	Crude	Ad-justed	ASD (rate)	Person years	Crude	Adjusted
**Onset of psychiatric disorders**								
Before conception								
During pregnancy								
Postpartum (1st year after de-livery)								
From 2nd year after delivery and onwards								
No P.Hist								

Note: Abbreviations. ASD: Autism Spectrum Disorders; P.Hist: Psychiatric History. To examine the onset of P.Hist in relation to risk of ASD, psychiatric diagnosis is included as a time-varying exposure in the model. Crude: Adjusted for birth year by cubic natural splines with 5 knots; Adjusted: Additionally adjusted for maternal and paternal education (0-9, 10-12, >12 years) and paternal income (SEK). [1]Incidence rate of ASD per 100,000 person years.

Table 2 Risk of ASD in offspring in relation to numbers of comorbid psychiatric disorders in parents

	Maternal P.Hist				Paternal P.Hist			
Analysis group	ASD (rate[1])	Person years	Crude	Adjusted	ASD (rate)	Person years	Crude	Adjusted
**Numbers of comorbid psychiatric disorders before delivery**								
One sub-category								
Two sub-categories								
Three and more sub-categories								
No P.Hist before delivery								
P.Hist in both parents [2]								
No P.Hist in any parent								

Note: Abbreviations. ASD: Autism Spectrum Disorders; P.Hist: Psychiatric History. Psychiatric sub-categories include (a) psychoactive substance use, (b) schizophrenia spectrum, (c) mood disorders, (d) neurotic, stress-related, behaviural and personality disorders, (e) disorders usually occurred in childhood and adolescence, and (f) other psychiatric disorders except for the mentioned above. Relative risk (rate ratio RR) with 95% confidence interval was calculated using Poisson regression models with robust standard errors. Crude: Adjusted for birth year by cubic natural splines with 5 knots; Adjusted: Additionally adjusted for maternal and paternal education (0-9, 10-12, >12 years) and maternal and paternal income (by natural cubic splines). [1]Incidence rate of ASD per 100,000 person years. [2] The estimated risk is for offspring of both parents with psychiatric disorders before delivery, compared to offspring of parents without psychiatric disorders before delivery.
